# Multiomics uncovers the epigenomic and transcriptomic response to viral and bacterial stimulation in turbot

**DOI:** 10.1093/gigascience/giaf077

**Published:** 2025-07-15

**Authors:** Oscar Aramburu, Belén Gómez-Pardo, Paula Rodríguez-Villamayor, Andrés Blanco-Hortas, Jesús Lamas, Pooran Dewari, Diego Perojil-Morata, Pierre Boudinot, Daniel J Macqueen, Carmen Bouza, Paulino Martínez

**Affiliations:** Department of Zoology, Genetics and Physicial Anthropology, University of Santiago de Compostela, 27002, Lugo, Spain; Department of Zoology, Genetics and Physicial Anthropology, University of Santiago de Compostela, 27002, Lugo, Spain; Department of Zoology, Genetics and Physicial Anthropology, University of Santiago de Compostela, 27002, Lugo, Spain; Department of Zoology, Genetics and Physicial Anthropology, University of Santiago de Compostela, 27002, Lugo, Spain; Department of Zoology, Genetics and Physicial Anthropology, University of Santiago de Compostela, 27002, Lugo, Spain; The Roslin Institute and Royal (Dick) School of Veterinary Studies, Division of Translational Bioscience, University of Edinburgh, Easter Bush Campus, EH25 9RG, Edinburgh, UK; The Roslin Institute and Royal (Dick) School of Veterinary Studies, Division of Translational Bioscience, University of Edinburgh, Easter Bush Campus, EH25 9RG, Edinburgh, UK; VIM for Virologie et Immunologie Moléculaires, Université Paris-Saclay, INRAE, UVSQ, VIM, 78350, Jouy-en-Josas, France; The Roslin Institute and Royal (Dick) School of Veterinary Studies, Division of Translational Bioscience, University of Edinburgh, Easter Bush Campus, EH25 9RG, Edinburgh, UK; Department of Zoology, Genetics and Physicial Anthropology, University of Santiago de Compostela, 27002, Lugo, Spain; Department of Zoology, Genetics and Physicial Anthropology, University of Santiago de Compostela, 27002, Lugo, Spain

**Keywords:** turbot, immune response, epigenomics, chromatin state, transcription factor

## Abstract

**Background:**

Uncovering the epigenomic regulation of immune response is essential for a comprehensive understanding of host defense mechanisms, though it remains poorly investigated in farmed fish.

**Results:**

We report the first annotation of the response of turbot (*Scophthalmus maximus*) immune cells to viral (poly I:C) and bacterial (inactive *Vibrio anguillarum*) mimics, integrating RNA sequencing with assay for transposase-accessible chromatin (ATAC) sequencing (ATAC-seq) and chromatin immunoprecipitation sequencing (ChIP-seq) (H3K4me3, H3K27ac, and H3K27me3) data from head kidney (*in vivo*) and primary leukocyte cultures (*in vitro*) 24 hours after stimulation. Among the 8,797 differentially expressed genes (DEGs), we observed enrichment of transcriptional activation pathways in response to *Vibrio* and immune pathways—including interferon-stimulated genes—for poly I:C. We identified notable differences in chromatin accessibility (20,617 *in vitro*, 59,892 *in vivo*) and H3K4me3-bound regions (11,454 *in vitro*, 10,275 *in vivo*) between stimulations and controls. Overlap of DEGs with promoters showing differential accessibility or histone mark binding revealed significant coupling of the transcriptome and chromatin state. DEGs with activation marks in their promoters were enriched for similar functions to the global DEG set but not always, suggesting key regulatory genes being in a poised state. Active promoters and putative enhancers were enriched in specific transcription factor binding motifs, many common to viral and bacterial responses. An in-depth analysis of chromatin state surrounding key DEGs encoding transcription factors was also performed to understand turbot immune response.

**Conclusions:**

This multiomics investigation provides an improved understanding of the epigenomic basis of turbot immune response to mimics of viral and bacterial stimuli, offering novel functional genomic information that provides a valuable resource for exploring immune regulation in flatfish.

## Background

The functional annotation of farm animal genomes is important for understanding traits with complex genetic architecture, such as disease resistance, growth, feed efficiency, or reproduction [1–4]. Until recently, functional annotation mainly focused on protein-coding genes using transcriptomics. Transcriptome annotation is now consolidated with robust pipelines [5], and throughout the years, transcriptome annotations for human [6], model species [7, 8], terrestrial livestock [9–11], and some aquaculture species [12, 13] have been published.

Noncoding regulatory elements, including promoters, enhancers, silencers, and insulators, have been studied in several livestock species but are mostly unexplored in aquaculture species. These elements play essential roles in regulating gene expression, and their state can change depending on tissue, cell type, sex, age, and health status [14]. Thus, annotation of regulatory elements in different contexts aids to address not only basic questions related to morphology and physiology [15, 16] but also functional genomic responses to environmental variation [17–19]. Genetic variation at noncoding elements also underpins phenotypic variation [20, 21] and can thus be leveraged to improve our ability to predict polygenic traits using genomic data [22]. In this respect, more than 90% of phenotype-associated single-nucleotide polymorphisms (SNPs) identified in human genome-wide association studies (GWAS) are located in noncoding regions [23], with similar results reported for livestock [24].

In the past decade, human and livestock functional annotation initiatives have investigated epigenetic mechanisms involved in gene regulation through the study of chromatin state modifications across the genome [25]. Chromatin can switch dynamically between active and inactive states in minutes to hours, leaving epigenetic footprints that can be transmitted vertically following DNA replication [26, 27]. Many different sequencing assays have been developed to infer chromatin epigenetic status, including chromatin accessibility (assay for transposase-accessible chromatin sequencing [ATAC-seq]) [28], protein–DNA interactions (chromatin immunoprecipitation sequencing [ChIP-seq]) [29] and long-range chromatin interactions (Hi-C) [30]. These and other assays are being applied by the FAANG Consortium [4, 31–33]. Current annotations of chromatin state and regulatory elements remain limited to a few terrestrial farm animal species [9, 16, 34, 35]. However, a catalog of regulatory elements is being generated for several important fish species used as model species [36] and in global aquaculture [13], currently the fastest-growing animal production sector [37, 38].

Turbot (*Scophthalmus maximus*) is a valuable farmed fish in Europe and Asia (more than 100,000 tons), with the highest production in China [39], followed by Spain [40]. Turbot is in its sixth generation of selective breeding and infectious disease outbreaks constitute one of the main challenges this young industry faces [39, 41]. This is a broader trend shared by global aquaculture, where infectious diseases cause losses totaling more than 5,000 M€ per year [42]. Functional annotation of the turbot transcriptome has been performed against high-quality reference genomes [41, 43–45], including for immune-organs stimulated with viruses [46], bacteria [47, 48], and parasites [49–52]. Candidate genes for disease resistance have been further explored by mapping differentially expressed genes (DEGs) within QTL regions [53–55]. However, limited attention has been given to noncoding regulatory elements, beyond a recent analysis of chromatin accessibility focused on early development [56]. How chromatin state and noncoding regulatory elements are regulated during immune responses remains undefined in turbot and scarcely explored in other farmed finfish.

The head kidney has been targeted in all previous functional genomics studies in turbot investigating pathogen responses [46–51, 55] due to its central role in fish immunity [57]. Head kidney is a key endocrine and lymphoid organ in most marine fishes and, analogous to the mammalian bone marrow, responsible for the production of multiple types of leukocytes, including B-lymphocytes, early-stage T-lymphocytes, and myeloid cells such as granulocytes and monocytes/macrophages [58–60]. Head kidney is a key site of adaptive immune responses and a niche for long-lived antibody-producing cells. It also contains many cells that play a central role in innate immune responses, following binding of pathogen-associated molecular patterns (PAMPs) to germline pattern recognition receptors (PRRs). These responses activate various effector cellular functions targeting pathogen destruction and clearance. Head kidney was therefore selected to characterize responses to pathogen mimics for its diversity of cell types and immune pathways.

## Data Description

The aim of this study was to generate the first comprehensive functional annotation of the innate immune response of turbot using a chromosome-level reference genome sequence (ASM1334776v1) [41]. Live fish (18 individuals) and primary immune cell cultures (18 cultures) were stimulated using mimics of viral (poly I:C) and bacterial (killed *Vibrio anguillarum*) infections and compared to controls to capture changes in the transcriptome alongside chromatin accessibility and epigenetic state by integrating RNA-seq, ATAC-seq, and ChIP-seq data. The experimental design and assays followed the protocols established in the European Commission Horizon 2020 AQUA-FAANG project (Grant Agreement 817923). We aimed to generate comparable datasets in response to the same bacterial and viral mimics in 6 commercially important farmed fish species: European seabass (*Dicentrarchus labrax*), gilthead seabream (*Sparus aurata*), rainbow trout (*Oncorhynchus mykiss*), Atlantic salmon (*Salmo salar*), common carp (*Cyprinus carpio*), and turbot (*Scophthalmus maximus*). Our results provide a deeper understanding of the epigenomic basis for innate immunity in turbot and a novel resource to prioritize genetic variation associated with noncoding elements regulating immune responses.

## Methods

### Animals

Thirty 8-month-old immature turbot specimens provided by Stolt Sea Farm SA (Ribeira, Spain) were housed in indoor tanks with recirculating seawater at the facilities of the Aquarium of the University of Santiago de Compostela (Spain) for a period of acclimation of 15 days at 16°C (Supplementary Table S1). All fish were fasted for 24 hours before stimulations were performed. Eighteen fish were stimulated *in vivo* by intraperitoneal injection, while the other 12 were used for leukocyte isolation for *in vitro* stimulation (see following sections). Fish were anesthetized by bath (MS-222; 100 mg/L) and then euthanized by anaesthetic overdose (MS-222; 150 mg/L) before tissue sampling. All animal procedures were approved by the Bioethics Committee of the University of Santiago de Compostela (body authorized according to R.D. 53/2013) and with the authorization of the Xunta de Galicia regional government.

### Protocols

Detailed protocols for the *in vivo* and *in vitro* stimulations, RNA isolation, ATAC-seq, and ChIP-seq (including library preparation) followed for turbot are available in the FAANG repository (data.faang.org; URLs for protocols in Supplementary Tables S1 and S2).

### *In vivo* immunostimulation

**Figure 1:**
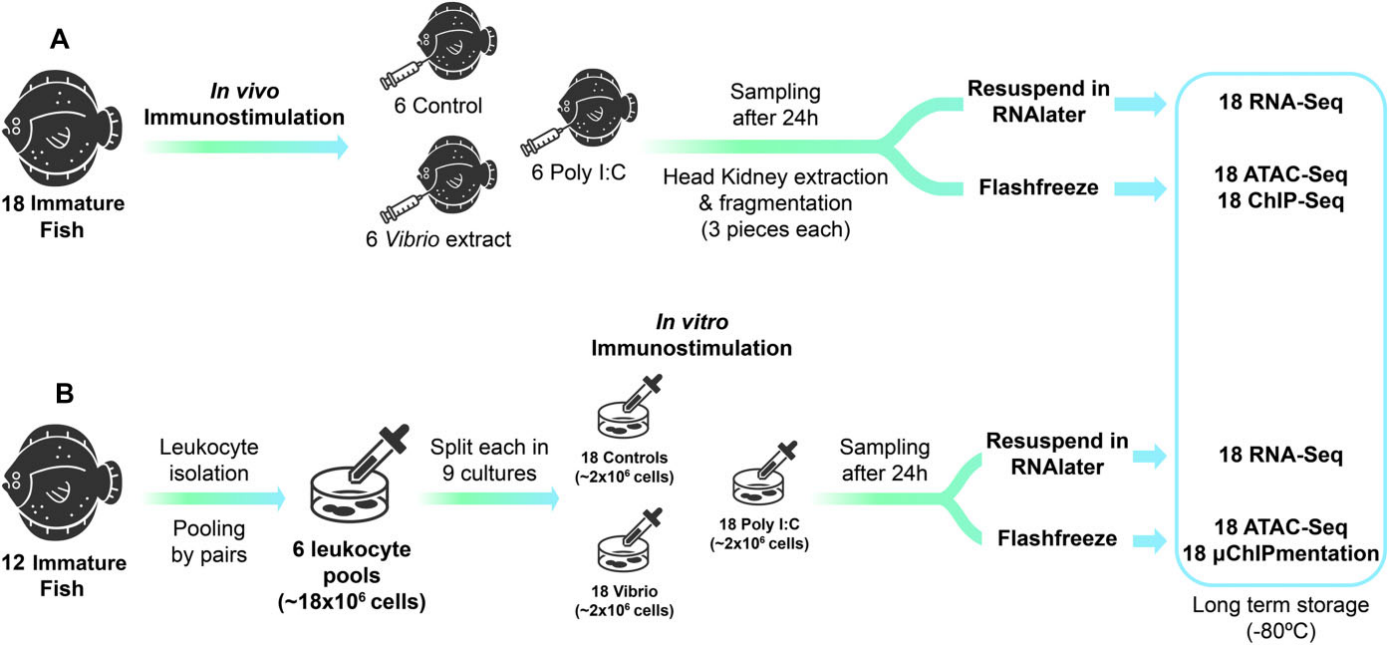
Experimental design followed for turbot immunostimulations: (A) *in vivo* immunostimulation of immature fish with subsequent sampling of head kidney and (B) *in vitro* stimulation of head kidney leukocytes of immature fish; 18 samples were used for RNA-seq, 18 for ATAC-seq, and 18 for ChIP-seq/µChIPmentation with antibodies to H3K4me3, H3K27ac, and H3K27me3 histone marks.

Six fish were used per experimental condition for the *in vivo* stimulations: (i) poly I:C for viral mimic immunostimulation, (ii) killed *V. anguillarum* for bacterial immunostimulation, and (iii) phosphate-buffered saline (PBS) for control. For poly I:C (Sigma P1530), we prepared a working stock at 5 mg/mL in PBS, preheated to 55°C (15 minutes) and cooled at room temperature (20 minutes) before use. Fish were then injected intraperitoneally with 5 µg per g fish weight. For bacterial immunostimulation, an extract of *V. anguillarum* (strain P0382; INRA) was used. Bacteria were cultured in a tryptic soy broth medium to an OD600 (optical density at 600 nm) of 1.5. The bacterial pellet (derived from 100 mL of full-grown culture) was washed in an isotonic solution of NaCl (9 g/L) 4 times and resuspended in 1 mL of the same solution. Bacteria were killed by incubation for 30 seconds at 100°C, allowed to cool at room temperature, and stored at −80°C. The bacteria extract was inoculated in each specimen and diluted in PBS (1:10) for a final volume of 100 µL. Control fish were injected with 100 µL PBS. After 24 hours, head kidney samples were extracted, washed with PBS, and cut into at least 3 pieces (> 20 mg each); 2 were flash frozen on dry ice for ATAC-seq and ChIP-seq, and the other was immersed in RNAlater (Thermofisher Scientific) for RNA extraction and RNA-seq. All samples were then stored at −80°C (Fig. 1A, Supplementary Table S2).

### *In vitro* stimulation: leukocyte isolation and culture

Leukocytes were isolated from 12 fish. The entire head kidney was aseptically isolated and placed in a Petri dish with 40 mL of cell isolation media (500 mL of Leibovitz L-15 medium [L-15], 10 mL fetal bovine serum [FBS, 2%], 0.02% EDTA). Samples were then cut into small pieces and passed through a 100-µm nylon mesh with constant flow of cell isolation media. Leukocytes were separated by centrifugation of 40 mL of the cell suspension gently layered in a 50-mL tube containing 51% Percoll (400 × *g*, 30 minutes, 4°C, no brakes). The interface layer was collected by centrifugation (400 × *g*, 10 minutes, 4°C) and washed 3 times with L-15 medium containing 0.1 FBS, keeping the pelleted leukocytes (Fig. 1B).

To ensure enough cells were available, samples were pooled by pairs after cell counting, and cell viability was evaluated by the trypan blue exclusion test, totaling 6 pools (each 18 × 10^6^ cells; Supplementary Table S1). Then, each pool was divided into 9 aliquots of 2 × 10^6^ cells (in 2 mL) that were dispensed into wells, for a total of 54 wells (6 pools × 9 aliquots): 18 stimulated with 20 µL poly I:C solution, 18 with 20 µL inactivated *V. anguillarum*, and the remaining 18 wells used as controls (Fig. 1B). All leukocyte cultures were incubated for 24 hours at 16°C. Cells were then collected in 2-mL Eppendorf tubes and pelleted at 500 × *g* for 5 minutes at room temperature. Eighteen pellets were resuspended in RNAlater and stored at −80°C for RNA-seq, while the other 36 were flash frozen in dry ice and stored at −80°C for ATAC-seq and µChIPmentation, respectively (Supplementary Table S1).

### Genome reference

The genome (ASM1334776V1) used as reference was assembled at the chromosome level and published by Martínez et al. [41]. It consists of 145 contigs (contig N50: 20.4 Mb) and 127 scaffolds (scaffold N50: 22.9 Mb), capturing 22 chromosomes representing 98.6% of the genome.

### RNA isolation and sequencing

After sample thawing, total RNA was extracted and purified using the miRNeasy Kit (QIAGEN) with specific modifications for (i) frozen head kidney (>20 mg), following a protocol for “Total RNA extraction for tissues,” and (ii) frozen leukocytes (2 × 10^6^ cells), following a protocol for “Total RNA extraction for frozen cells” (Supplementary Table S2). RNA integrity and quantity were evaluated in a Bioanalyzer (Bonsai Technologies) and in a NanoDrop ND-1000 spectrophotometer (NanoDrop Technologies). RNA integrity number (RIN) averaged 8.3 across all samples, always above 7.3. RNA samples were delivered to Novogene for library preparation using NEBNext Ultra Directional RNA Library Prep Kits for Illumina and sequenced using an Illumina NovaSeq S4 platform to generate 150-bp paired-end reads.

### RNA-seq data processing

RNA-seq data were processed using nf-core/rnaseq 3.10.1 [61] run with default parameters, using the turbot Ensembl genome ASM1334776v1 [41] as reference. In brief, the pipeline evaluated quality of raw reads using FASTQC (RRID:SCR_014583) [62] and trimmed adapters and low-quality bases using Trim Galore (RRID:SCR_011847) [63]. Reads were then mapped using STAR (RRID:SCR_004463) [64]. Normalized transcript read counts were obtained using RSEM (RRID:SCR_000262) [65]. After nf-core processing, the resulting count tables were filtered to remove genes with expression below 5 transcripts per million (TPM < 5) and represented in only 1 sample across all conditions.

### Differential gene expression and Gene Ontology analysis

DEGs between stimulated and control samples were identified using the R/Bioconductor package DESeq2 v1.38.1 (RRID:SCR_015687) [66]. Genes with false discovery rate (FDR)–adjusted *P* < 0.05 were considered DEGs. Functional enrichment of the DEG lists was performed using ShinyGO v0.77 (RRID:SCR_019213) [67]. Gene Ontology (GO) terms for Biological Process of each list were ranked by statistical significance (FDR-adjusted *P* < 0.05). All expressed genes across conditions were used as the background for GO analyses.

### ATAC-seq: Library preparation and sequencing

Following a standard protocol for “Nuclei isolation for ATAC-seq procedures” (Supplementary Table S2), the frozen head kidney fragments (>20 mg; *in vivo* assay) and cell pellets (2 × 10^6^ cells; *in vitro* assay) were thawed and resuspended in 1 mL TST buffer. Each tissue fragment was cut into smaller pieces with a scalpel, mashed with the rubber back of a syringe, and filtered through a 40-µm cell strainer, while cell pellets were resuspended by gentle pipetting. The number and integrity of nuclei were assessed with a hemocytometer (minimum of ∼50,000 nuclei in a 16.5-µL suspension; ∼3,000 nuclei/µL) before carrying out the Tn5 transposase reaction with Illumina Tagment DNA TDE1 enzyme (37°C, 30 minutes, 1,000 rpm; Illumina) following the standard “OmniATAC protocol” (Supplementary Table S2) [68]. The resulting DNA was purified with a MinElute PCR purification kit (Qiagen), and DNA concentration was assessed with a Qubit device using the dsDNA HS kit (ThermoFisher Scientific). Library amplification (10–12 PCR cycles) was carried out using the NEBNext Ultra II DNA Library Prep Kit (New England Biolabs), with IDT for Illumina UD Indexes (96×, Plate A, Set 1; Illumina). Library size selection was performed to remove fragments below 180 bp and above 700 bp using AMPure XP beads (Beckman Coulter). Finally, DNA fragment size distribution was assessed with the Bioanalyzer High Sensitivity DNA Assay kit (Agilent Technologies). ATAC-seq libraries were delivered to Novogene to be sequenced on an Illumina NovaSeq S4 platform generating 150-bp paired-end reads.

### ChIP-seq and µChIPmentation: Library preparation and sequencing

The frozen head kidney fragments (>20 mg) and leukocyte pellets (2 × 10^6^ cells) were thawed on ice. Following a standard “ChIP-seq” protocol (Supplementary Table S2), the tissue fragments were transferred into a Dounce homogenizer containing a protease inhibitor cocktail (PIC, Roche; 1 tablet in 50 mL PBS) solution immersed in ice and homogenized using pestles A and B (from less to more plunger adjustment). The leukocyte pellets were resuspended in the PBS and PIC solution. The amount and quality of nuclei were assessed with a hemocytometer and trypan blue staining (>10 million cells for head kidney; >100,000 for leukocyte cultures). Due to the low number of nuclei recovered from the leukocyte cultures, a µChIPmentation protocol (Diagenode) was used, following a modified protocol (Supplementary Table S2).

In both cases, chromatin crosslinking was done using a 1% formaldehyde solution followed by quenching with glycine (0.125 M). Nuclei were pelleted and resuspended in complete sonication buffer, while leukocyte nuclei were resuspended in Hanks’ Balanced Salt Solution (HBSS; Thermo Fisher Scientific)—tL1 buffer (Supplementary Table S2). Chromatin was sheared using a Covaris S2 focused ultrasonicator with the following parameters: 2% duty cycle, intensity 3, with 200 cycles per burst, at 4°C for 8 minutes and 6 minutes for head kidney tissue and leukocytes, respectively.

The immunoprecipitation was performed using Diagenode antibodies for 3 marks: H3K4me3 (marking active promoter regions; cat. C15410003; 1.3 µg/µL), H3K27ac (marking active enhancer and promoter regions; cat. C15410196; 2.8 µg/µL), and H3K27me3 (marking Polycomb repressed regions; cat. C15410195; 1.1 µg/µL). For head kidney samples, antibodies were coupled with prewashed protein A and protein G beads and the tubes left under rotation overnight (∼16 hours) at 4°C, following the standard ChIP-seq protocol. After washing the beads and decrosslinking, the samples were purified using the MinElute PCR purification kit (Qiagen) and later quantified with DNA HS Qubit (ThermoFisher Scientific). Immunoprecipitated chromatin was stored at −20°C until library preparation, using a Microplex v3 kit (Diagenode). For the leukocyte samples, the µChipmentation kit for histones (Diagenode) was used for chromatin immunoprecipitation and ChIP-seq library preparation (Supplementary Table S2).

Before sequencing, the quantity and quality of purified libraries were assessed using the Qubit DNA HS kit (ThermoFisher Scientific) and the High Sensitivity DNA Assay kit (Agilent Technologies), respectively. A minimum of 60% of the chromatin was required to have a size distribution between 200 and 700 bp (centered around 350–400 bp). ChIP-seq libraries were delivered to Novogene for sequencing on an Illumina NovaSeq S4 platform generating 150-bp paired-end reads.

### ATAC-seq and ChIP-seq data processing

ATAC-seq and ChIP-seq data were processed using the nf-core/atacseq v1.2.2 and nf-core/chipseq v1.2.2 pipelines [68], respectively, run with the narrow_peak option for ATAC-seq and H3K4me3 and H3K27ac ChIP-seq datasets, and with the broad_peak option for H3K27me3. The other parameters were kept by default. Quality assessment of the reads was carried out with FASTQC (RRID:SCR_014583) [62] and adapters and low-quality bases trimmed with Trim Galore (RRID:SCR_011847) [63]. Reads were mapped to the turbot genome using BWA (RRID:SCR_010910) [69]. Further filtering was done with SAMtools (RRID:SCR_002105) [70], BAMtools (RRID:SCR_015987) [71], and pysam (RRID:SCR_021017) [70]. Genome-wide immunoprecipitation (IP) enrichment relative to controls was done with deepTools (RRID:SCR_016366) [72], and broad/narrow peaks were called using MACS2 (RRID:SCR_013291) [73]. Once the nf-core run was finished, suboptimal replicates with very low peak numbers were excluded after visualizing their bigwig files on the Integrative Genomics Viewer (IGV; RRID:SCR_011793) [74] (Supplementary Table S3B).

### ChIP-seq and µChIPmentation blacklist

To improve the signal-to-noise ratio of the ChIP-seq and µChIPmentation data, a blacklist consisting of high signal and low mappability regions was constructed using ChIP-seq and µChIPmentation inputs, including 21 control ChIPseq turbot samples (ENA accession PRJEB57784), following a publicly available pipeline [75]. The mappability of the turbot genome for read lengths of 100 bp and 150 bp (*k*-mers 100 and 150) was quantified using the umap software package (RRID:SCR_018217) [76]. The generated mappability files were fed into the ENCODE blacklist software [77] to generate the blacklist. Alignment and peak files derived from the nf-core pipeline were filtered with BAMtools to remove reads and peaks located in the blacklist regions.

### Differential histone modification regions and differentially accessible regions

Significant differential histone modification regions (DHMRs) and differentially accessible regions (DARs) (adjusted *P* < 0.05) between stimulated samples and controls were identified using DiffBind (RRID:SCR_012918) [78] with default settings.

### Integration of ChIP-seq and ATAC-seq data with RNA-seq data

For each condition tested, the promoters of DEGs overlapping with regions tagged as DARs and/or DHMRs were identified. When applicable, a hypergeometric test was performed to check the significance of overlapping between each pair (*P* < 0.05, Bonferroni correction).

### Chromatin state inferences

Genome-wide chromatin states for each condition were predicted using ChromHMM (RRID:SCR_018141) [79] integrating the ChIP-seq data (µChIPmentation for *in vitro* samples) for the 3 histone marks (H3K4me3, H3K27ac, and H3K27me3) and ATAC-seq data. Chromatin state prediction was performed by testing ChromHMM models, including from 8 to 15 states, keeping the one that returned the most biologically relevant chromatin states for head kidney and leukocyte data separately [34, 36, 80]. The genome-wide distribution of resulting chromatin states was visualized on IGV (RRID:SCR_011793) [74]. Regions annotated as enhancer-related states by ChromHMM were retrieved, and each stimulation dataset was compared against its respective control to identify potential enhancer-related regions. Then, potential enhancers annotated as “intergenic” or “intron” were kept as differential enhancer-state regions for further analysis.

### Transcription factor motif analysis

Enriched transcription factor binding motifs (TFBMs) included in the HOMER software (RRID:SCR_010881) [81] were identified using the *findMotifsGenome.pl* function (settings: -size given -mask -mset vertebrates) in the different lists of promoter-associated DHMRs/DARs and putative enhancers for each condition. Random genomic regions with GC-content matching each input genomic list were used as the background for automatic motif analysis by HOMER. Following the recommended guidelines for the HOMER program, only TFBMs with *P* < 0.05 (after Bonferroni correction) and percentage of target sequences >10% were considered enriched.

GO analysis of the transcription factors (TFs) predicted to bind enriched TFBMs was performed with Metascape (RRID:SCR_016620) [82] using zebrafish *Danio rerio* as the reference. Finally, a selection of TF genes that were DEGs and contained promoter regions overlapping DARs or DHMRs were explored with IGV (RRID:SCR_011793) [74].

## Results

### Raw sequencing data and sample metadata

A total of 186 multiomic datasets were produced in this study, including RNA-seq (36), ATAC-seq (36) and ChIP-seq (108; 36 per histone mark, plus 6 ChIP-seq input controls). Full information on samples and metadata is shared in Supplementary Tables S1 and S2.

### RNA-seq

**Figure 2:**
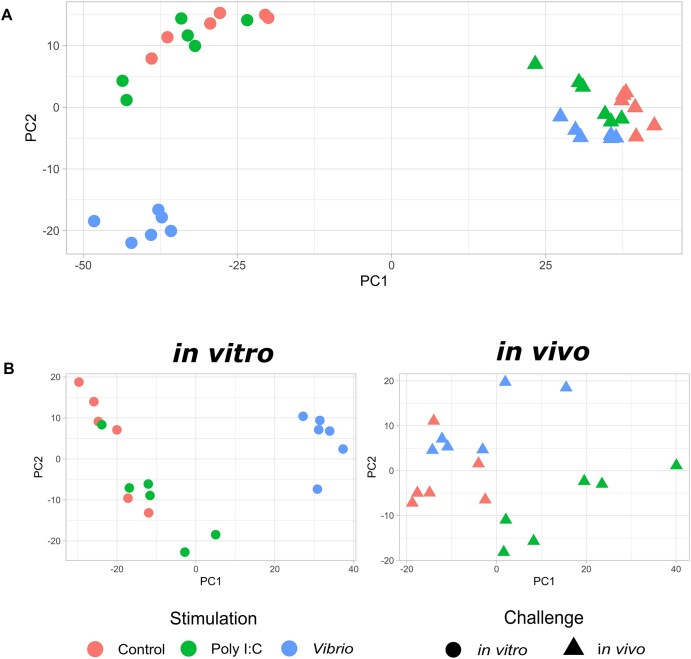
PCA of the *in vivo* and *in vitro* transcriptomic response to stimulation with poly I:C and *Vibrio*. (A) PCA of the whole transcriptomic dataset. (B) Independent PCAs for the *in vitro* and *in vivo* transcriptomic assays.

On average, 69,580,246 raw reads per library were produced across the 36 RNA-seq samples, with 97.2% mapping to the turbot genome (Supplementary Table S3A). Principal component analysis (PCA) showed that 77% of the transcriptome variance was explained by PC1, separating the *in vivo* and *in vitro* stimulations (Fig. 2A). Despite all 3 *in vivo* conditions (head kidney samples) grouping together, a suggestive spatial segregation was observed mainly across PC1, with the control at one end and the *Vibrio* stimulation at the other. For the *in vitro* stimulations (primary cell cultures of kidney-isolated leukocytes), PC2 clearly separated the *Vibrio* stimulation from poly I:C and control samples, the last 2 showing some overlap (Fig. 2B).

Differential expression analysis was performed by comparing each stimulated condition to the respective controls, both for the *in vitro* and *in vivo* stimulations. In total, 8,797 DEGs were identified across all comparisons (Table 1, Supplementary Table S4). For the *in vitro* stimulations, a stronger response was observed for *Vibrio* than poly I:C stimulation, both for up- and downregulated genes. Meanwhile, poly I:C showed a higher number of DEGs than *Vibrio* for the *in vivo* stimulations both for up- and downregulated genes (Table 1, Supplementary Table S4). Overall, more DEGs were detected in the *in vitro* than in the *in vivo* stimulations (7,940 vs. 5,758, respectively).

**Table 1:** Number of differentially expressed genes (DEGs) for the *in vitro* and *in vivo* stimulations with *Vibrio* and poly I:C

Stimulant	Stimulation	Downregulated DEGs	Upregulated DEGs	Total DEGs
*Vibrio*	*In vitro*	3,217	3,321	6,538
	*In vitro*	929	910	1,839
Poly I:C	*In vitro*	544	858	1,402
	*In vitro*	1,918	2,001	3,919

### Functional enrichment among DEGs

GO analysis identified enriched biological processes for up- and downregulated DEGs in all conditions (Supplementary Table S5). Among the downregulated DEGs, metabolism, cell cycle, and cytoskeleton organization terms were enriched for the *in vitro* stimulations, while a limited number of terms were detected for *in vivo* stimulations (Supplementary Table S5).

More abundant and specific enriched GO terms were detected among the upregulated DEGs. Although RNA metabolism was enriched for most stimulations, poly I:C stimulation was linked to a more specific activation of key immune functions (particularly *in vitro*), such as interferon-stimulated genes and cytokine pathways, and regulation of Toll-like receptors, besides more general immune terms. DEGs for the *Vibrio in vitro* stimulation were enriched for immune-related terms associated with cytokine and several transport pathways, whereas *Vibrio in vivo* upregulated genes displayed strong enrichment of terms related to cytoskeleton organization, tissue development, and syncytium formation (Supplementary Table S5).

**Figure 3:**
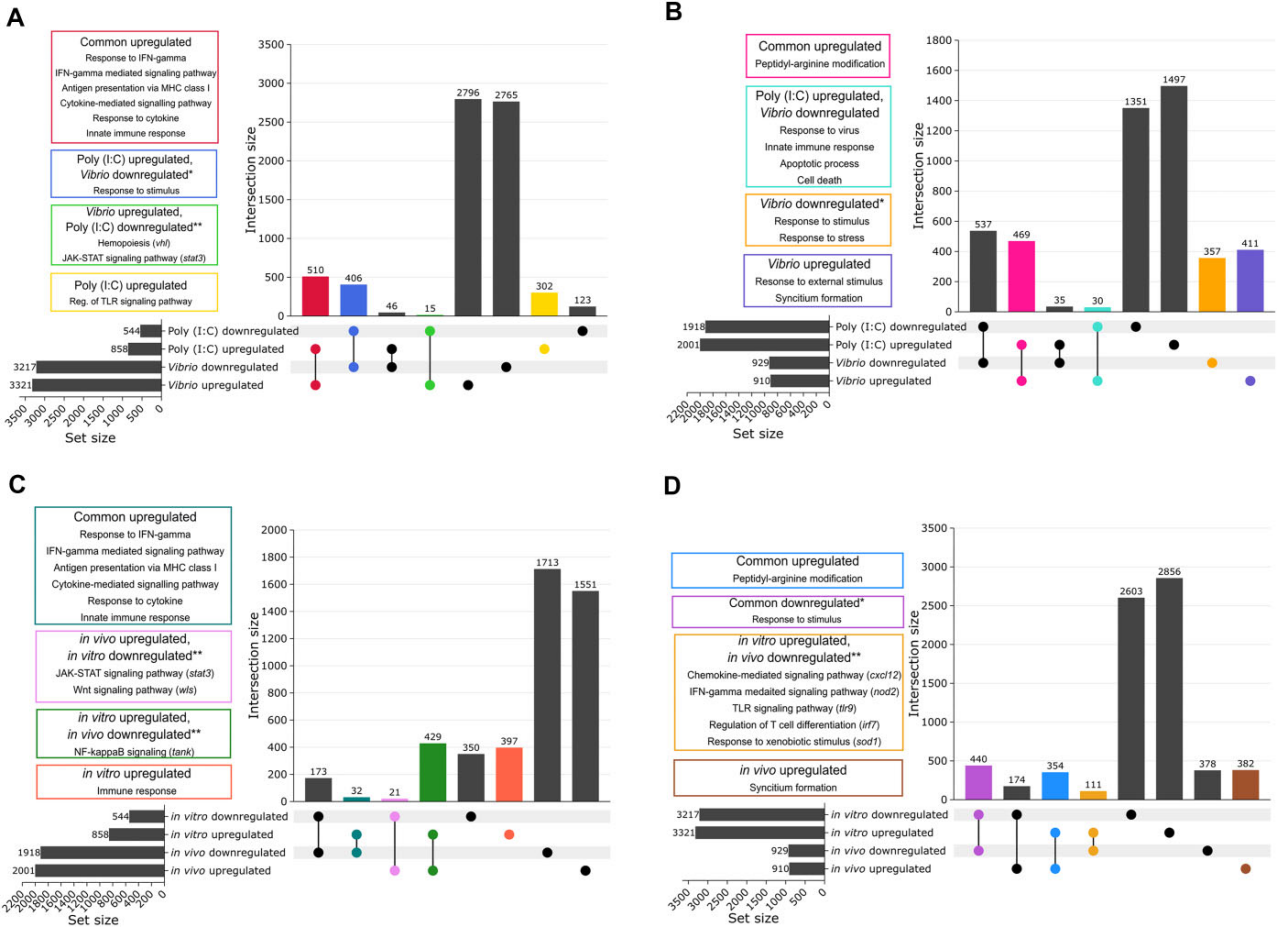
UpSet plots illustrating overlap of DEGs for (A) *in vitro* poly I:C vs. *Vibrio*, (B) *in vivo* poly I:C vs. *Vibrio*, (C) Poly I:C *in vitro* vs. *in vivo*, and (D) *Vibrio in vitro* vs. *in vivo*. The colored boxes show enriched GO terms related to the immune system found in the subsets with the same color. Asterisks (*) highlight cases where suggestive immune-related GO terms were identified, despite not being significantly enriched. Double asterisks (**) highlight specific genes (in parentheses) associated with relevant immune functions.

Comparing upregulated DEGs between conditions, important immune-related GO terms were commonly enriched between both poly I:C and *Vibrio in vitro* stimulations, as well as the poly I:C *in vitro* and *in vivo* stimulations (Fig. 3, Supplementary Table S6, Supplementary Table S7). These included interferon type I stimulated genes (*socs1a, socs1b, nod2, nmi*), cytokine signaling (the same genes plus *traf2* and *il15ra*), and MHC-I pathways (*erap1b, tapbpl, tapbp*2). Activation of transcription, protein localization, and a small number of terms associated with “peptidyl-arginine modification” (*prmt1, prmt3, prmt5, prmt7*), critical to maintain self-antigenic integrity, were commonly overrepresented in poly I:C and *Vibrio in vivo*, as well as between the *in vitro* and *in vivo Vibrio* stimulations (Fig. 3, Supplementary Table S6, Supplementary Table S7).

We detected several DEGs regulated in opposite directions for poly I:C and *Vibrio* stimulations (Table 2, Fig. 3, Supplementary Table S6), suggestive of divergent immune response to bacteria and virus. Among the *in vivo* upregulated DEGs for *Vibrio* and downregulated for poly I:C, we observed enrichment of general immune and inflammatory functions, including genes such as *il-1b, traf4a*, and *f2r*. Conversely, immune-related genes, including *nod2, sting1, irf1b*, and *apaf1*, were downregulated for *Vibrio* and upregulated for poly I:C *in vivo*. Interestingly, many orthologs of human and mouse genes involved in the type-I IFN response [83, 84] were upregulated in the poly I:C stimulations but downregulated with *Vibrio* extracts, especially in the *in vivo* stimulation (Table 2).

**Table 2:** Immune-related DEGs showing opposite responses following *Vibrio* and poly I:C stimulations

Comparison	Differentially expressed genes (DEGs)
*In vitro*—upregulated by poly I:C, downregulated by *Vibrio*	***aldh2-like, arhgap22*** *, arrdc2, asb9-like* ***ascc3, atxn7l1****, bpifcl, c1qa*, ***cep170b****, chaf1b, cnnm4b*, ***dync1h1, ece2b, ermp1-like, gna14, gpr155a, has1,ip6k2-like, mapk8a, mycbp2****, nfkb1*, ***notch2****, pld4-like, psda, rilp, serinc1-like, sgk2b, sp100.1*, ***spsb4a, sptbn1****, stim2b, synpr*, ***tank****, tent4b*, ***tlr8****, tmem269-like, tnk2b, tpm4b*, ***trim25-like, trim35-14, usp1****, vcanb*
*In vitro*—upregulated by *Vibrio*, downregulated by poly I:C	*bcat-like, ctns, gid8b-like, pcxb*, ***psat1****, rab11b-like, rtf2, sfxn2*, ***slc25a43, stat3****, thoc7, tollip, vhl, vps26c, vps41*
*In vivo*—upregulated by poly I:C, downregulated by *Vibrio*	*adora4a*, ***ankrd10b, apaf1****, arhgap12b, atg9a*, ***atxn7l1****, bpifcl, camkk1b*,***casp1a-like****, cylda, emilin1*, ***fem1c****, gfral*, ***gna14****, gnptab, il22ra2*, ***irf1b****, nlrc3-like*, ***nod2****, nsmce4a, otulina*, ***parp15-like, pstpip1b, pstpip2, rasgef1b****, rnf146*, ***rnf170, sting1, themis2, txk****, ubp15, ucp2-like*, ***xcr1-like, xkr8-like***
*In vivo*—upregulated by *Vibrio*, downregulated by poly I:C	*aebp1, alpl, anxa13l, anxa1a, cald1a, capn2b, chchd6a, col17a1-like, f2r, ggh-like, icn-like, il-1b, il8-like, il11-like, kdm2aa, lims2, map1lc3b, mfsd10, msto1, olfm5-like, osbpl10, plpp1like, plxnb2b, slc4a4-like, tlr13-like, traf4a, vat1, zan-like*

Turbot orthologs of human/mouse type I IFN stimulated genes (ISGs) are highlighted (in bold upregulated and underlined downregulated), based on the database Interferome [83, 84]. Note that most of these genes are upregulated in response to poly I:C stimulation but downregulated in response to *Vibrio* stimulation.

Considering condition-specific DEGs (Fig. 3, Supplementary Table S6), those upregulated by poly I:C *in vitro* were enriched for terms associated with the Toll-like receptor signaling pathway (*usp4, tasl, irak3*), while transcriptional activation terms were found among upregulated DEGs for *Vibrio in vitro*. For the *in vivo* stimulations, transcriptional activation GO terms were enriched for upregulated DEGs after poly I:C stimulation, whereas response to stimulus (explained by *tlr3, hamp, tnip1, fxc1a*, and *ccr12a*, among other genes) and syncytium formation (explained by *kirrel3l, jam2a*, and *plekho1b*) were enriched for *Vibrio*.

### ATAC-seq and ChIP-seq

**Figure 4:**
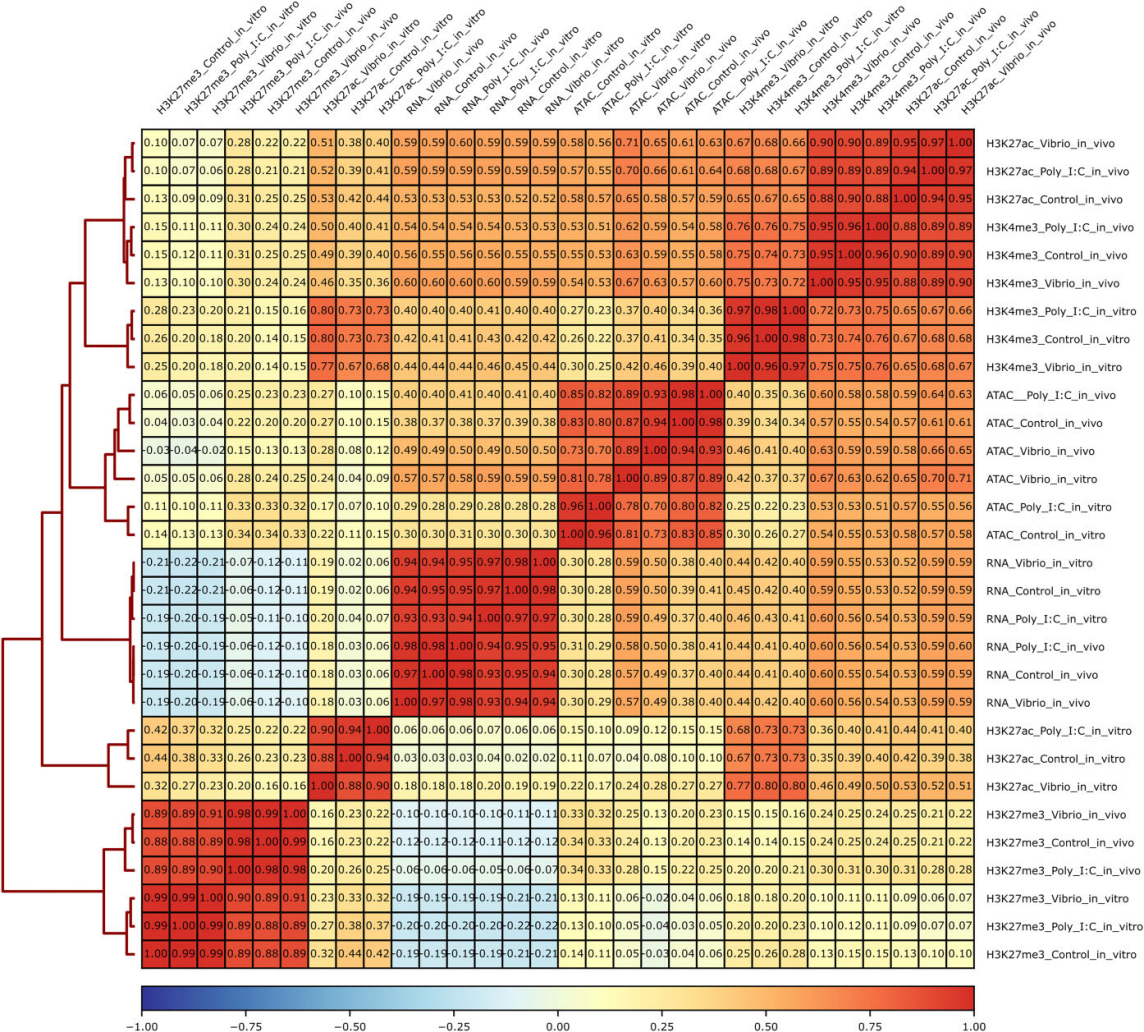
Heatmap of the hierarchical clustering (Spearman correlation) of the *in vivo and in vitro* transcriptomic and epigenomic response to stimulation with poly I:C and *Vibrio* for the RNA-seq, open chromatin ATAC-seq, and histone ChIP-seq (H3K4me3, H3K27ac, H3K27me3) samples.

Most libraries showed >95% read mapping to the turbot genome, except four ATAC-seq *Vibrio in vitro* libraries with lower mapping rates due to the presence of the bacterial DNA in the cell culture (average 54%) (Supplementary Table S3B). On average, we identified 24,251 (*in vitro*) and 62,013 (*in vivo*) peaks per sample for ATAC-seq; 26,199 (H3K4me3), 13,461 (H3K27ac), and 27,765 (H3K27me3) peaks for the *in vivo* ChIP-seq data; and 15,870 (H3K4me3), 5,363 (H3K27ac), and 38,784 (H3K27me3) peaks for the *in vitro* ChIP-seq data. Hierarchical clustering of the samples clustered them by technique and condition (Fig. 4), excluding the H3K27ac mark, where *in vivo* and *in vitro* clusters for poly I:C were separated. This is concordant with the behavior observed for regulatory elements for this experimental condition, as discussed in future sections. In addition, the marks associated with repression (H3K27me3) and activation (H3K4me3) of regulatory elements showed, respectively, the expected negative and positive correlation with RNA-seq data. A similar pattern was observed in the PCA plot, where techniques were segregated mostly across PC1, unless for the repression (H3K27me3), mostly explained by PC2, also responsible for differentiation across conditions (Supplementary Fig. S1). *Vibrio* showed the greatest differentiation among the stimulations, while poly I:C and controls were mostly intermingled, particularly for ATAC-seq (*in vitro* and *in vivo*) and H3K4me3–ChIP-seq (*in vitro*).

### ChIP-seq blacklist

Certain genomic regions obscure epigenetic analyses because of anomalous, unstructured, or high signal due to particular genomic features [77]. Using the 21 ChIP-seq and µChIPmentation input controls (ENA project PRJEB57784), we constructed a blacklist of low-confidence genomic regions for turbot ChIP assays (Supplementary Table S8). On average, 6.98% (∼39 Mb) of the turbot genome was included in the blacklist, consisting of high input signal (5.58%) and low mappability (1.40%) regions (Supplementary Fig. S2).

### Chromatin state annotation

**Figure 5:**
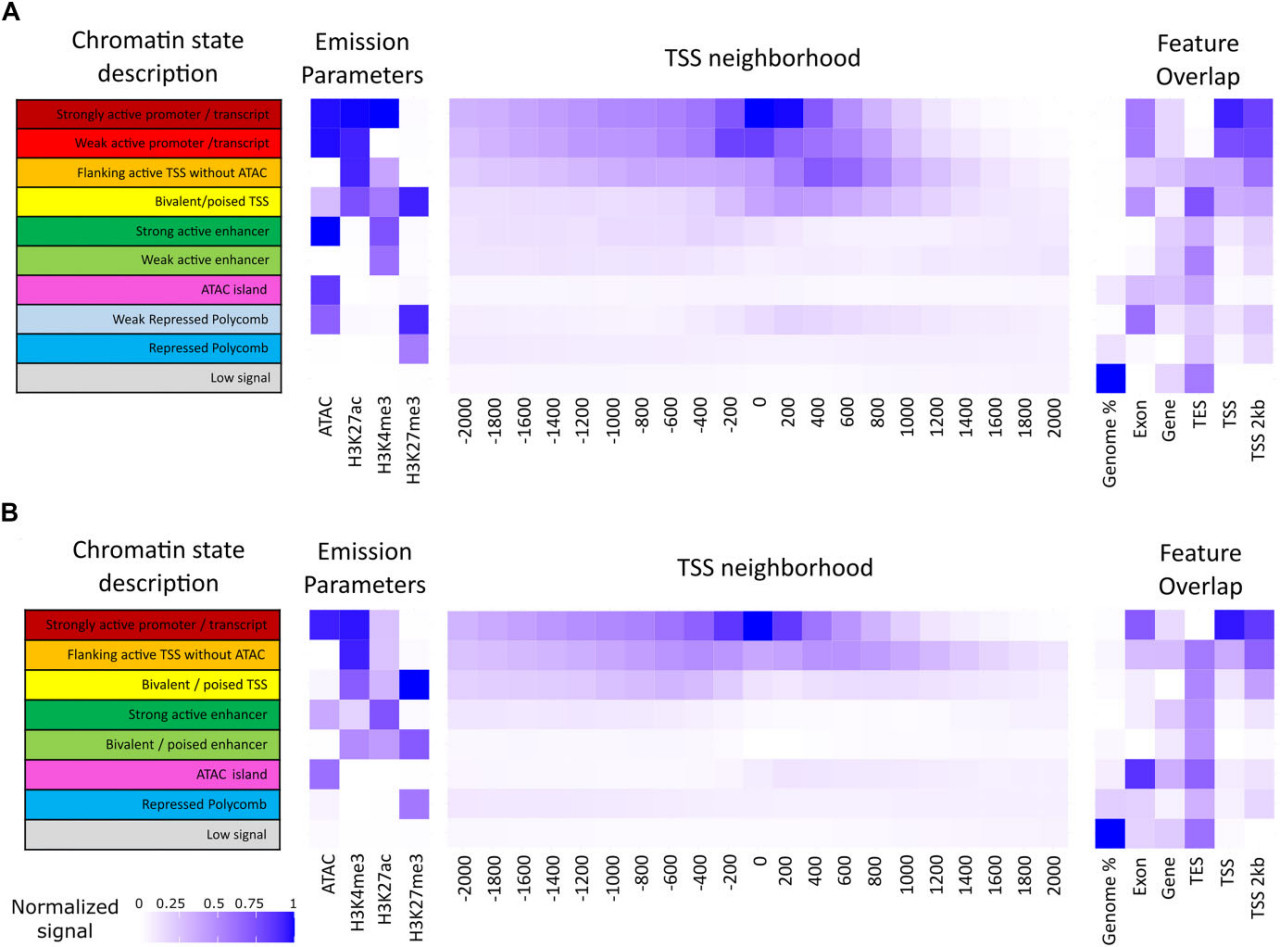
Chromatin state models of the turbot genome: (A) 10-state model based on *in vivo* samples and (B) 8-state model based on *in vitro* samples. Heatmaps of the emission parameters for each chromatin state are shown for ChIP-seq data with 3 histone marks and ATAC-seq (left), the neighborhood around the TSS (middle), and the emission parameters of each chromatin state considered alongside features within the turbot genome (right). TES: transcription end site; TSS: transcription start site.

Genome-wide chromatin state predictions were produced using ChromHMM employing the ChIP-seq and ATAC-seq data from stimulated and control samples from head kidney tissue (*in vivo*) and head kidney–derived leukocytes (*in vitro*) (Fig. 4, Supplementary Table S9). For the *in vivo* samples (Fig. 5A), a 10-state model was chosen, including promoters/transcription start sites (TSSs; states 1, 2, 3 and 4), potential enhancer regions (states 5 and 6), ATAC islands (state 7; i.e., ATAC peaks lacking histone marks), repressed regions (states 8 and 9) and low signal regions (state 10). For the *in vitro* leukocytes (Fig. 5B), an 8-state chromatin model was chosen, including promoters/TSSs (states 1, 2, and 3), potential enhancer regions (states 4 and 5), ATAC islands (state 6), repressed regions (state 7), and low signal regions (state 8). For each chromatin state map, ±2-kb regions around the TSS showed signal specifically for chromatin states 1, 2, 3, and 4 for head kidney (*in vivo*) and states 1, 2, and 3 for leukocytes (*in vitro*), mostly corresponding to promoter regions and/or transcriptionally active regions.

### DARs and DHMRs following stimulation

We next aimed to identify regulatory regions in the turbot genome affected by the immune stimulation. Significant DARs and DHMRs (FDR-adjusted *P* < 0.05) comparing *Vibrio* and poly I:C stimulations to control samples were identified (Table 3, Supplementary Table S10). Globally, a higher number of DARs and DHMRs were detected for up- than for downregulated regions, more *in vivo* than *in vitro*, and more for *Vibrio* than poly I:C comparisons. Additionally, histone marks H3K27ac and H3K27me3 showed much lower DHMRs than H3K3me3 or ATAC-Seq DARs.

**Table 3:** DARs and DHMRs for the *in vitro* and *in vivo* immune stimulations with viral and bacterial PAMPs

PAMP	Stimulation	Assay	Downregulated DAR/DHMR	Upregulated DAR/DHMR	All DAR/DHMR
*Vibrio*	*In vitro*	ATAC	2,739	17,878	20,617
		H3K4me3	699	10,755	11,454
		H3K27ac	2	623	625
		H3K27me3	6	1,211	1,217
	*In vivo*	ATAC	43,159	16,733	59,892
		H3K4me3	287	9,988	10,275
		H3K27ac	0	3	3
		H3K27me3	4	0	4
Poly I:C	*In vivo*	ATAC	0	0	0
		H3K4me3	0	0	0
		H3K27ac	0	20	20
		H3K27me3	0	0	0
	*In vivo*	ATAC	25	15	40
		H3K4me3	0	1,036	1,036
		H3K27ac	0	38	38
		H3K27me3	126	0	126

### Association of ATAC-seq and ChIP-seq data with RNA-seq data

We tested if DARs and DHMRs for H3K4me3 and H3K27ac marks annotated as promoter/TSS regions (up to −1 kb upstream of TSSs) corresponded to DEGs under the same experimental conditions (hypergeometric distribution test, *P* < 0.05). DARs and DHMRs were much more overrepresented at the promoter regions of up- rather than downregulated DEGs (Table 4, Supplementary Table S11), suggesting changes in chromatin state associated with the activation of genes. We performed GO enrichment analyses of those upregulated DEGs within each experimental condition (*P* < 0.05, Table 4). Significant enrichment (FDR < 0.05) included several metabolic activities and particular immune functions, including antigen processing/presentation and apoptotic/cell death pathways (Supplementary Table S12). Specifically, enriched terms were RNA processing—particularly transfer RNA, ribosomal RNA, and noncoding RNA—as well as ribosome biogenesis and translation for the *Vibrio in vitro* stimulation. In contrast, the terms associated with the *Vibrio in vivo* stimulation were linked to protein localization to the nucleus and organelles, DNA replication, and carbohydrate metabolism. The term “peptidyl-arginine modification” was also enriched, involving *prmt1, prmt3, prmt5*, and *prmt7* genes, as outlined before for the whole DEG analysis (RNA-seq). The poly I:C *in vivo* stimulation showed enrichment in immune-related processes, including “antigen processing and presentation via MHC-I” (explained by *erap1b, tapbpl*) and “regulation of programmed cell death” (explained by *apaf1, bida, casp8* and *10, socs3a, grinab, bcl2l10*; Supplementary Table S12). No correlation was found between chromatin accessibility in promoter regions and differential expression among selected genes related with T-cell costimulation, pro- and anti-inflammatory cytokines, and class IV TRIM genes typically involved in antiviral defense (Supplementary Fig. S3).

**Table 4:** Overlap of promoter DARs and DHMRs with DEG promoters (hypergeometric test, *P* < 0.05). Significant results are underlined. Refer to Supplementary Table S11 for further details

	DARs/DHMRs at promoter regions		Integration of DARs/DHMRs at promoter regions of DEGs			
			Down		Up	
Experimental condition	Down	Up	DEG	DAR/DHMR + DEG	DEG	DAR/DHMR + DEG
ATAC poly I:C *in vitro*	-	-	544	-	858	-
ATAC *Vibrio in vitro*	55	4,922	3,217	1	3,321	1,259
ATAC poly I:C *in vivo*	4	1	1,918	-	2,001	-
ATAC *Vibrio in vivo*	1,350	6,115	929	34	910	395
H3K4me3 poly I:C *in vitro*	-	-	544	-	858	-
H3K4me3 *Vibrio in vitro*	53	4,289	3,217	14	3,321	1,164
H3K4me3 poly I:C *in vivo*	-	368	1,918	-	2,001	151
H3K4me3 *Vibrio in vivo*	22	4,024	929	3	910	242
H3K27ac poly I:C *in vitro*	-	-	544	-	858	-
H3K27ac *Vibrio in vitro*	-	72	3,217	-	3,321	49
H3K27ac poly I:C *in vivo*	-	4	1,918	-	2,001	3
H3K27ac *Vibrio in vivo*	-	1	929	-	910	1
Active prom poly I:C *in vitro*	-	-	544	-	858	-
Active prom *Vibrio in vitro*	106	6,744	3,217	15	3,321	1,782
Active prom poly I:C *in vivo*	4	370	1,918	-	2,001	151
Active prom *Vibrio in vivo*	1,263	7,082	929	37	910	463

prom: promoter.

### TFBM analysis

To establish TFs potentially associated with chromatin state regulation following immune stimulation, the enrichment of TFBMs within DARs/DHMRs annotated as promoter or enhancer regions was examined. Significant enrichment was detected for all stimulations. On average, across stimulations, 56.5 (range: 0–109) significantly enriched TFBMs were detected for upregulated promoters, with only 1 significantly enriched TFBM detected in downregulated promoters for *Vibrio in vivo*. Meanwhile, 5 (range: 1–10) and 35.75 (range: 1–61) respective TFBMs were enriched for downregulated and upregulated enhancers (Supplementary Table S13).

**Figure 6:**
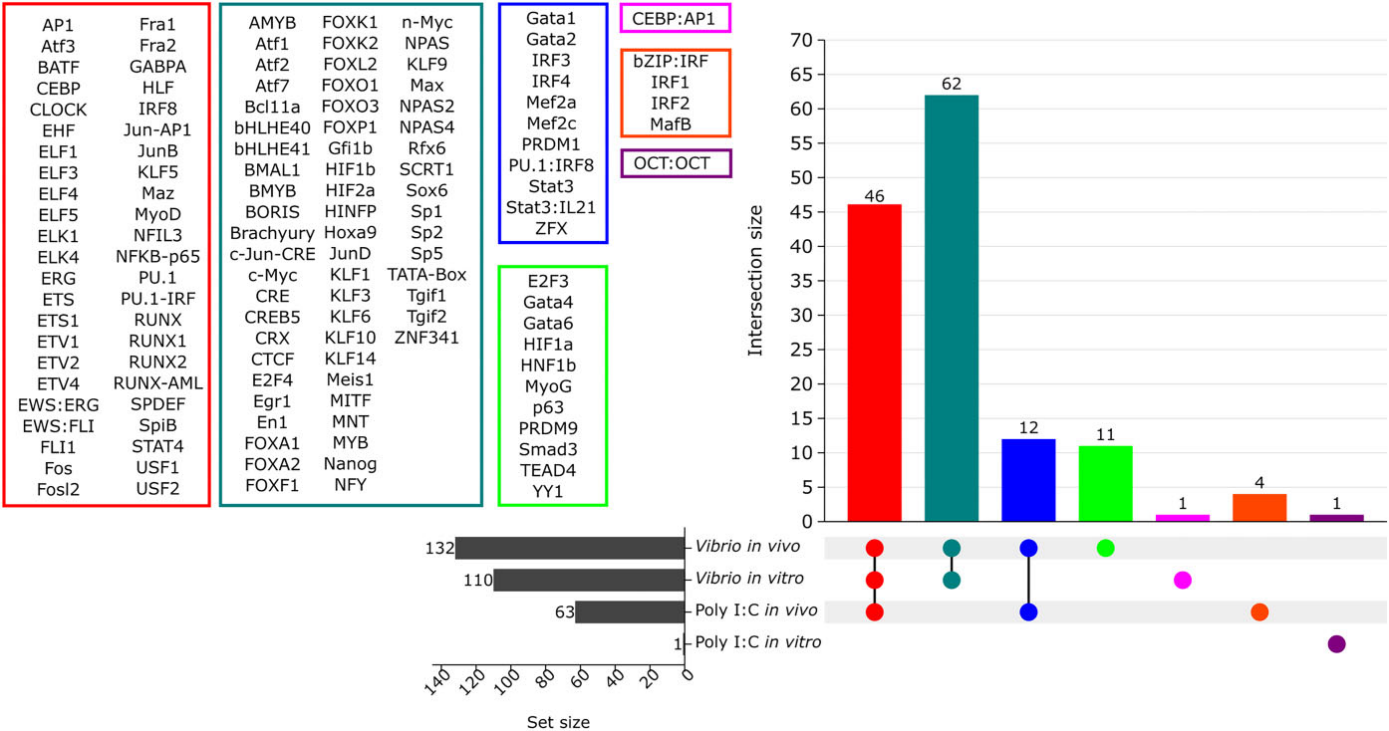
UpSet plot diagram illustrating overlap of enriched TFBMs in upregulated promoter and enhancer regions in response to the different stimulants. Each colored box shows the TFBM found.

Most TFBMs and associated TFs predicted for active promoters and enhancers were enriched in multiple stimulation conditions: 46 TFs were shared by both *Vibrio* stimulations and *in vivo* poly I:C, 62 TFs were shared for both *Vibrio* stimulations, and 12 TFs were shared for both *in vivo* stimulations (Fig. 6; Supplementary Table S13).

To further investigate the functions of TFs with enriched motifs, we used the Molecular Complex Detection algorithm (MCODE) and protein–protein interaction (PPI) data to identify connected functions among differentially expressed TFs, as well as among TFs with DARs or DHMRs on their promoters, with a special focus on immune-related functions (Supplementary Fig. S4, Supplementary Table S14).

For the *Vibrio in vitro* stimulation, interconnected clusters of TFs associated with hemopoiesis and immune functions, particularly the FGF signaling pathway, MAPK signaling pathway, lymphocyte activation, lymph vessel development, and oxidative stress response, were identified. For the *Vibrio in vivo* stimulation, we detected clusters of TFs associated with similar functions: hemopoiesis, MAPK signaling pathway, and myeloid leukocyte differentiation and cellular senescence (Supplementary Fig. S4). Finally, for the poly I:C *in vivo* stimulation, hemopoiesis, FGF signaling pathway, MAPK signaling pathway, myeloid cell development and differentiation, and regulation of cell differentiation showed connected TF subsets.

**Figure 7:**
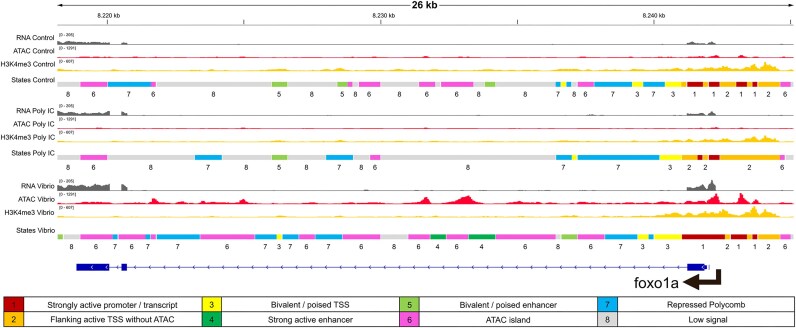
Chromatin state predictions and RNA/ATAC/ChIP-seq tracks in the transcription factor coding gene *foxo1a* (ENSSMAG00000033089; C03:8,218,876–8,241,954), which was upregulated and showed DAR around the promoter for *Vibrio in vitro* response. Transcription factor binding motifs for FOXO1A were also enriched in the promoter DARs of DEGs upregulated in response to *Vibrio in vitro*.

Using the Integrative Genomics Viewer (IGV), we visualized variation in chromatin state around the promoters of 9 TF-DEGs selected from Supplementary Table S14, as well as the Toll-like receptor *tlr3* gene, to show differences following activation by the different stimulants (Fig. 7, Supplementary Figs. 5 and 6).

## Discussion

This study represents the first epigenomic analysis of the turbot head kidney, the primary hematopoietic and lymphoid organ of teleosts, in response to viral (poly I:C) and bacterial (inactivated *V. anguillarum*) mimics. While head kidney transcriptomic responses to bacteria, virus, and parasites have been extensively investigated in turbot [46, 48, 50–52, 85–87], the epigenetic regulation of chromatin states following immune stimulation has not been researched before. The head kidney response was explored through intraperitoneal injection, reflecting the response in the whole body, including interactions among immune organs, and through *in vitro* stimulation of primary leukocyte cultures, reflecting their direct interaction with stimulants. The use of the same viral and bacterial mimics will enable further comparative evaluation among the 6 fish species included in the AQUA-FAANG project pertaining to 5 teleost orders (Salmoniformes, Cyprinidontiformes, Spariformes, Perciformes, and Pleuronectiformes). We first discuss the transcriptional response to immune stimulations, which provide context to the chromatin assays performed to understand how transcription is regulated from transcription factors up to immune genes in the turbot.

### Transcriptomic response to immune stimulation

Upregulated genes with key immune roles, such as interferon and cytokine pathways and Toll-like receptor regulation, were found in both poly I:C stimulations and in *Vibrio in vitro* stimulation. Interferons (IFNs) are a subset of class II cytokines with crucial roles in antimicrobial defense, especially against viruses, but also intracellular bacteria [88]. Interestingly, *tlr3*, encoding the Toll-like receptor 3, which induces IFN production after interaction with poly I:C [89], was upregulated in the *Vibrio in vivo* stimulation but not differentially expressed in poly I:C *in vivo* and downregulated after both *in vitro* stimulations. We also checked the chromatin state distribution along the *tlr3* gene (Supplementary Fig. S6) in both *in vitro* and *in vivo* conditions. Overall, no significant changes were observed. Most of the chromatin states found within the promoter and the gene body were annotated as strongly active promoters/transcripts, flanking active TSSs without ATAC and ATAC islands (shared by both chromatin state models). Additionally, we observed a weak active promoter/transcript state annotated only in the *in vivo* condition. Overall, the lack of chromatin regulation suggests that promoter accessibility is not alone sufficient for *tlr3* expression. The asynchronism between chromatin accessibility of regulatory elements and gene expression has been reported previously, with promoter peaks preceding gene expression, and distal regulatory elements such as enhancers appearing accessible when gene expression starts [90–92]. In fact, we observed an active enhancer signal (both weak and strong states) close to the distal promoter region of *tlr3* in the *Vibrio in vivo* stimulation only, which may be linked to the expression of this or other genes in that condition.

Regardless, signal exhaustion 24 hours postinjection (hpi) might explain *tlr3* downregulation *in vitro*, and this would not be expected for the *in vivo* poly I:C stimulation, where the peak of the *in vivo* poly I:C response for this gene has been reported to occur later in turbot (3 days postinjection) [93]. Long-term activation of *tlr3* by poly I:C has been reported to cause long-term physiological impairment in different mouse tissues [94, 95]. The tissue expression profile of *tlr3* can, however, vary between tissues and cell types in different species; although similar expression was observed between zebrafish and rainbow trout tissues [96], this gene’s expression was limited to liver and digestive organs in *Fugu* [97], while in human vs. mouse, *tlr3* was expressed in different myeloid cell types [98].

We cannot discard alternative hypotheses related to changes or diversification of the TLR3 signaling pathway in flatfish or specifically in turbot, as suggested here by *tlr3* activation in response to *Vibrio in vivo* stimulation and to the parasite *Philasterides dicentrarchi* by Figueras et al. [43]. In fact, we identified other interferon-stimulated DEGs in poly I:C both *in vitro* and *in vivo*, including *socs1a* and *b* (suppressor of cytokine signaling 1), *nod2* (nucleotide binding oligomerization domain containing 2), and *nmi* (N-Myc interactor), typically participating in type I IFN responses [99, 100]. *socs1* is a conserved, inducible negative regulator of IFN [99], which has been described before in other finfish species as being responsive to viral [101, 102] and bacterial stimulation [103]. This gene is involved in tissue homeostasis following the IFN response, marking a critical checkpoint in immune homeostasis as an expected player to be found in extended immune responses [104].

On the other hand, *nod2*, which encodes for a highly conserved intracellular receptor triggering innate antibacterial and antiviral signaling pathways in fish, including IFN [105–108], and *nmi*, another highly conserved ISG, which increases STAT-mediated transcription in response to IFN-gamma in mammals and fish [109–112], were also activated after poly I:C stimulation. The activation of conserved genes involved in positive and negative regulation of IFN signaling suggests a fine adjustment to an intense IFN response to recover tissue homeostasis [113, 114]. Also, the upregulation of these 4 key genes after *Vibrio in vitro* stimulation is consistent with past reports that place IFN regulation as a key point of the immune response [115, 116]. In this regard, comparative analysis in the AQUAFAANG project will enlighten conserved and specific mechanisms of immune response regulation across different farmed fish lineages. Future studies in turbot should explore shorter periods following *in vitro* stimulation to provide further understanding of the head kidney response to viral and bacterial mimics, as done in other species [117, 118].

Upregulated DEGs shared by both *in vivo* stimulations were involved in the activation of transcription and translation and protein modification/localization. This included 4 members of the conserved immune-related protein arginine methyltransferase family (*prmt1, prmt3, prmt5*, and *prmt7*), enriched under the term “peptidyl-arginine modification.” *prmt* family members regulate transcription and translation and are also involved in signal transduction during inflammation and responses against poly I:C and bacterial lipopolysaccharide (LPS), in both mammals and finfish species [119–122]. These 4 genes were also activated in both *Vibrio* stimulations, suggesting an important role in the response to *Vibrio*.

We also verified if immune genes could be regulated in opposite directions after stimulation with viral and bacterial mimics, which could reflect antagonistic immune responses. Increased susceptibility to bacterial superinfections induced by innate antiviral responses has been reported in several models in mammals [123] in type I and type II IFN responses [124, 125]. Such mechanisms are likely present in teleosts due to the high conservation of these pathways, and in fact, different patterns of resistance to viral and bacterial infections were observed among isogenic rainbow trout lines [126]. However, no negative correlations for resistance to bacteria and viruses have been observed in the few studies addressing this issue in relation to selective breeding [127–129]. Indeed, understanding the cellular basis of antagonistic responses to viruses and bacteria in fish will require more research, but here we identified examples of opposite gene expression responses, including genes from the core type I IFN response, conserved between teleosts and humans [84, 110], such as *sting1* (stimulator of interferon response CGAMP interactor 1) [92, 130], *irf1b* (interferon regulatory factor 1) [131, 132], and *nod2* (mentioned above), downregulated by *Vibrio* and upregulated by poly I:C *in vivo*. Reciprocally, the critical proinflammatory cytokine *il1b* (interleukin 1 beta) [133, 134] and the regulator of inflammation *traf4a* (TNF receptor associated factor 4) [135, 136] were upregulated by *Vibrio* but downregulated by poly I:C. Interestingly, most genes induced by poly I:C and repressed by *Vibrio* extracts had human/mouse ISG orthologs (22/42 *in vitro*, 18/34 *in vivo*); in contrast, many genes induced by *Vibrio* extracts and repressed by poly I:C, especially *in vivo*, had human/mouse orthologs downregulated by type I IFN. Overall, these observations indicate that a significant part of these contrasted responses is mediated by genes functionally conserved between fish and mammals.

### Epigenetic assays and their association with transcriptomic response

Activation and binding of TFs to regulatory regions lies at the top of specific transcriptome cascade responses [137, 138]. In our study, the exploration of promoters of differentially expressed TFs, also involving DARs and DHMRs, barely showed changes in the chromatin state distribution between treatments (Supplementary Fig. S5). This was the case for *egr1* (early growth response 1), which plays a key role in cell survival, macrophage proliferation, and cell death in teleost and mammals [139, 140] and regulates the expression of *il1b* and *cxcl2* (CXC motif chemokine ligand 2). The same was observed for *meis1* (myeloid ecotropic viral integration site homeobox 1), related to hematopoiesis in mammals and teleost [141, 142]. In all these examples, the TF genes showed bivalent/poised promoters in the 3 *in vivo* samples (control, *Vibrio*, and poly I:C). A similar situation was found *in vitro*, where promoters of genes, such as *mitf* (melanocyte inducing transcription factor), showed activation signals regardless of the experimental condition. In previous studies, these bivalent/poised states have been interpreted as a mechanism allowing rapid responses, for example, for genes induced during the proinflammatory response [118, 143] or during mammal embryonic development and in germ cells [144, 145]. Thus, the expression of primary response genes, such as the TF genes exemplified above, may be allowed by permissive chromatin states.

The presence of differential activation signals at promoter regions, either in the form of open chromatin regions or the histone marks H3K4me3 and H3K27ac, is associated with differential gene expression [146]. H3K27ac is widely accepted as one of the most dynamic activation marks in eukaryotes [147]. Interestingly, we did not find important changes between experimental conditions in our study, where H3K4me3 was the most dynamic among the ChIP-seq marks. We suspect that the increase in H3K4me3 could also be an effect of sampling being conducted 24 hpi, especially for the *in vivo* stimulations. Previous studies have reported that H3K4me3 may not have an active role in activating transcription, an effect that should be detectable early after stimulation, but instead of marking transcriptional activity itself, this suggests that H3K4me3 modification may have a role in maintaining transcriptional consistency or memory of previous states [148].

DEG promoters overlapped with DARs/DHMRs for upregulated genes in most conditions (Table 4). GO enrichment of DEGs with activation signals at promoters (Supplementary Table S12) was similar to that observed with the whole DEG dataset (Supplementary Table S5). *Vibrio* stimulations were particularly enriched in terms associated with transcription activation but also with some immune functions, while poly I:C (especially *in vivo*) showed a more specific response, enriched in immune-related processes (Supplementary Table S6). However, DEGs associated with some key immune functions in the transcriptomic analysis, such as response to cytokine stimulus, did not show DARs/DHMRs, suggesting that these gene promoters could be already accessible before stimulation. Cytokines are one of the core initiators of the inflammatory response, and thus a bivalent state, ready for activation or inactivation of expression, might explain this observation (Fig. 6).

Regardless of predicted chromatin states around gene promoters, we observed an increased expression of immune-related genes following poly I:C and *Vibrio* stimulation, suggesting further chromatin unpacking (Table 3), even if chromatin state is not strongly affected. In fact, many differential regions were detected between stimulations, particularly DARs (i.e., ATAC-seq) and DHMRs (for H3K4me3) for *Vibrio*. In comparison, fewer DARs and DHMRs were detected for the poly I:C stimulations. This could be interpreted as a return, especially *in vitro*, to the native chromatin state after 24 hpi, which may suggest the initial response at this point is exhausted, as suggested by the transcriptomic data.

The intersection between DEGs and DARs was specifically inspected for a selection of immune genes related to T-cell activation (as a checkpoint for the transition to cellular adaptative immunity), pro- and anti-inflammatory cytokines (related to early and late immune response, respectively), and class IV TRIM genes (associated mainly with antiviral responses; Supplementary Fig. S3). Again, no clear correlation was found between differential promoter accessibility and differential gene expression in any of the conditions. In fact, most promoters for the targeted genes were not even differentially accessible, adding to the suggestion that poised states are a common feature among immune-related genes. Both *cd28* (coactivator) and *ctla4* (coinhibitor), conserved mediators in vertebrate T-cell activation [149, 150], did not show significant changes in chromatin accessibility between comparisons, except for *Vibrio in vitro*, where the promoter was differentially accessible. The same was true at the level of gene expression, though, as both *Vibrio* assays showed significant downregulation of the *cd28* gene. Looking at anti-inflammatory cytokines, the conserved IL10 and a subunit of IL35 (encoded by *ebi3*) were significantly upregulated in *Vibrio in vitro* and poly I:C *in vivo* in the case of *il10*, as well as in both poly I:C assays for *il35* (*ebi3*). For proinflammatory cytokines, different sets of genes were regulated for *Vibrio in vitro* (*il17a*/*f1, il17a*/*f2*, upregulated), poly I:C *in vivo* (*il1b*, downregulated), and *Vibrio in vivo* (*il1b*, upregulated). All conditions showed upregulation of both anti- and proinflammatory cytokines, except poly I:C *in vivo*, which showed upregulation of all these genes. The mixture of anti- and proinflammation signals suggests an advanced response to the stimulants, while illustrating the complexity of their coordinated contributions to the regulation of inflammatory response. Finally, class IV TRIM genes, which participate in innate immunity [151], mostly showed significant upregulation in poly I:C stimulations, consistent with their conserved role in antiviral responses [152]. Interestingly, little significant differential expression was found in the *Vibrio* stimulations (none *in vivo*).

The signals detected for the 3 histone marks and open chromatin regions were integrated to define chromatin states in *in vivo* and *in vitro* assays. Two chromatin state models differing in the number of states for the *in vitro* (8) and the *in vivo* (10) assays were annotated based on the emission parameters of each epigenetic mark. Due to the high conservation of the assayed histone marks among eukaryotes [153, 154], we also compared our chromatin state models with other studies using the same histone marks [31, 118, 155, 156]. The rationale for running 2 models arose from the differences in biological material and different methodologies applied for the ChIP-seq assays (classic ChIP-seq in the *in vivo* assays vs. µChIPmentation in *in vitro* assays). In fact, the 9- and 10-state iterations of the *in vitro* assay resulted in 1 and 2 redundant chromatin states, respectively, reducing the quality of the models.

Indeed, even a 7-state model (strongly active promoter/transcript, flanking active TSSs without ATAC, bivalent/poised TSSs, strong active enhancer, ATAC island, repressed polycomb and low signal states) showed nearly similar emission parameters and distribution in the TSS neighborhood, suggesting a general conservation of chromatin states between the whole head kidney and the kidney leukocyte fraction. Only 1 *in vitro* exclusive chromatin state (bivalent/poised enhancer) and 3 *in vivo* exclusive chromatin states (weak active promoter/transcript, weak enhancer, and weak repressed polycomb) were inferred from the 2 independent models. Although the absence of these 3 weak states in the *in vitro* assay can be explained due to the limited resolution of an 8-state model compared to a 10-state one, we hypothesize that the presence of a bivalent enhancer state in the *in vitro* but not the *in vivo* assay can be the result of the enrichment of immune cells and lack of contribution of nonleukocytic cells missing in the *in vitro* assay, where bivalent/poised states have been suggested to be relevant in the fast regulation of immune pathways [143, 157, 158]. Altogether, the integration of all histone marks in chromatin states allowed us to identify the co-occurrence of different histone marks, uncovering different combinations of activator signals (H3K4me3 + H3K27ac) and with repressor signals (H3K27me3).

TFBMs enriched within DARs/DHMRs in the promoters and enhancer-state regions predicted by ChromHMM of upregulated DEGs provided a more detailed picture of regulatory elements changed by the immune stimulations (Fig. 6, Supplementary Table S13). Most of the enriched TFBMs were shared between at least 2 treatments, which supports their general regulatory function. Among those shared between most stimulations (excluding poly I:C *in vitro*), we found several TFBMs of the ETS family, which are involved in cell proliferation, apoptosis, and lymphocyte development [159]. We also found TFBM enrichment for PU.1, a master TF for the myeloid lineage, that promotes chromatin accessibility, also identified in a similar study in pig [118, 160, 161]. PU.1 is thought to promote binding of other TFs enriched in our study, including AP1. This protein is activated by TLRs during the immune response [162] and regulates gene expression in response to cytokines, stress, and bacterial or viral infections [163], as well as notably the IFN regulatory factors (IRFs, particularly IRF3, IRF4, and IRF8). The IRFs are key regulators of innate antiviral and antibacterial responses in vertebrates, including fish [164, 165]. The observed ontology enrichment clustering of these TFs reinforces the similarities between the conditions in our study (Supplementary Fig. S4).

To illustrate the functionality of our turbot epigenomic atlas, we explored the chromatin state changes in a selection of 9 TF genes (Fig. 7, Supplementary Fig. S5). These genes were differentially expressed and showed DARs/DHMRs in their promoters. Immune-responsive chromatin state remodeling was found, for instance, for *irf8* (interferon regulatory factor 8) in the *Vibrio in vitro* stimulation. Here, an extension of the “strongly active promoter” state around the TSS region was visible compared to the nonstimulated and poly I:C stimulated samples, alongside an overall expansion of the “strongly active transcript” and “ATAC island” states downstream. IRF8 is a key regulator of the NF-κB signaling pathway during inflammation, along with IRF3 [166]. Both IRF genes were differentially expressed in poly I:C and *Vibrio in vitro* stimulations (*irf8* also in poly I:C *in vivo*) and showed promoter DARs in *Vibrio* stimulations.

Another example was found in *Vibrio* and poly I:C *in vivo* stimulations for *bcl11a* (BCL11 transcription factor A), a key regulator of dendritic cell differentiation [167] and a negative regulator of p53 [168]. This gene was differentially expressed in both stimulations but only showed a DAR promoter in response to *Vibrio* stimulation. The promoter/TSS region was annotated by ChromHMM as “weak repressed polycomb” in the control, which changed in both the *Vibrio* and poly I:C stimulations, extending to a “bivalent/poised” state, and, in the case of *Vibrio*, showing an extension to a “strongly active promoter/transcript” state. Weak and strong signals of active enhancers were also detected in the poly I:C and *Vibrio* stimulations within the second intron of *bcl11a*, which were not present in the controls (Supplementary Fig. S5). A similar situation was found *in vitro* for *foxo1a* (Forkhead box O1), a TF-coding gene that participates in mucosal (innate) immune response, regulating the expression of antimicrobial peptides and promoting phagocytosis during bacterial and parasitic infections [169, 170]. This gene was differentially expressed in *Vibrio in vitro* and showed an extension of the “strongly active promoter” state compared to the control and especially the poly I:C stimulation, while conserving the “bivalent/poised TSS” stretches downstream of the promoter. This was followed by many “ATAC islands” along the gene that were also present, although more scarcely, in the control and almost absent in poly I:C. Finally, a “medium enhancer” state was detected within the first intron, present only in the *Vibrio* condition. These observations highlight the relevance of epigenetic chromatin marks at the first intron and other intronic regions associated with gene expression modulation [171, 172], as target regulatory annotations to explore functional variants for disease resistance and selective breeding.

## Conclusions

In summary, our study provides the first atlas of regulatory elements in turbot head kidney and leukocytes during the early response to viral and bacterial stimulation, as a contribution to the AQUA-FAANG project within the umbrella of the FAANG initiative. The integration of ATAC-seq and ChIP-seq data suggests that changes in chromatin state distribution were not as frequent between stimulations and controls as expected. However, the presence of DARs and DHMRs between the stimulations and the controls, which broadly overlapped with DEGs, provides clear evidence for gene regulation at the epigenetic level that underpins changes in gene expression driving immune functions.

## Potential Implications

Overall, this epigenomic atlas will help to decode the molecular mechanisms underlying turbot immune responses to viral and bacterial stimuli and offers a novel resource for developing selective breeding strategies for controlling diseases, one of the main concerns of the turbot industry. Future work will benefit from linking the regulatory annotations generated in this study with genetic variants defined by whole-genome resequencing [13], to help prioritize causal genetic variants for disease resistance traits underpinned by gene expression responses to pathogens.

## Additional Files

**Supplementary Fig. S1**. (A) Full and (B) separate PCAs for each assay and stimulation of the *in vivo* and *in vitro* epigenomic response to stimulation with poly I:C and *Vibrio* for the open chromatin ATAC-seq and histone ChIP-seq (H3K4me3, H3K27ac, H3K27me3) samples.

**Supplementary Fig. S2**. Genomic distribution of the ChIP-seq high signal regions (pink) and low mappability regions (blue) of the turbot genome included in the blacklist for each of the 22 chromosomes. The dotted lines represent the approximate centromere positions, based on the microsatellite analysis of Martínez et al., 2008.

**Supplementary Fig. S3**. (A) Promoter accessibility and gene expression (log_2_FC, ordinates) in the *in vitro* assays of key immune genes related to (i) regulation of activation of T-cell immune response (*cd28* and *ctla4*), (ii) anti-inflammatory (*il10, il35* subunit *ebi3*) and (iii) proinflammatory cytokines (*il1b, il17a/f1, il17a/f2, il17a/f3, il17c, il17d*, and *tnf*), and (iv) all class IV *trim* genes annotated in the turbot genome. (B) Promoter accessibility and gene expression (log_2_FC, ordinates) in the *in vivo* assays of key immune genes related to (i) regulation of activation of T-cell immune response (*cd28* and *ctla4*), (ii) anti-inflammatory (*il10, il35* subunit *ebi3*) and (iii) proinflammatory cytokines (*il1b, il17a/f1, il17a/f2, il17a/f3, il17c, il17d*, and *tnf*), and (iv) all class IV *trim* genes annotated in the turbot genome.

**Supplementary Fig. S4**. Functional interconnection of active TFs detected in response to the different stimulants. (A) Ontology enrichment clusters of upregulated TFs showing promoter DARs/DHMRs with TFBMs enriched in promoter or enhancer DARs/DHMRs of DEGs detected in *Vibrio in vivo* stimulation. (B) Protein–protein interaction MCODE network of upregulated TFs induced by *Vibrio in vivo* stimulation. Each cluster is colored with the most statistically significant term among terms that cluster together. The size of each term is given by −log_10_p, and the stronger the similarity among nearby terms, the thicker the edges between them. Functional interconnection of active TFs detected in response to the different stimulants. (C, D) Ontology enrichment clusters of upregulated TFs showing promoter DARs/DHMRs with TFBMs enriched in promoter or enhancer DARs/DHMRs of DEGs detected in *Vibrio in vitro* and Poly I:C *in vivo* stimulation, respectively. (E, F) Protein–protein interaction MCODE network of upregulated TFs induced by *Vibrio in vitro* and Poly I:C *in vivo* stimulation, respectively.

**Supplementary Fig. S5**. (A) IGV screenshots of the chromatin structure and RNA-seq, ATAC-seq, and ChIP-seq tracks of different transcription factor coding genes, selected due to them (i) being differentially expressed (DE) in at least one of the conditions, (ii) having differentially accessible regions (DARs) or differential histone modification regions (DHMRs) in at least one of the conditions, and (iii) being among the TFBMs enriched in response to at least one of the conditions. Each chromatin state model annotation is located in the table below the IGV screenshots. *arnt* was DE and showed DAR and H3K4me3-DAR in its promoter in *Vibrio in vitro*, as well as being among the TFBMs enriched in response to both *Vibrio in vitro* and *in vivo. irf8* was DE in both *Vibrio* and Poly IC *in vitro* but only showed DAR in its promoter in *Vibrio in vitro*, as well as being among the enriched TFBMs in response to *Vibrio in vitro, in vivo*, and Poly IC *in vivo*. (B) *bhlhe40a* and *bhlhe40b* were DE and showed DAR in its promoter in *Vibrio in vitro*, as well as being among the enriched TFBMs in response to *Vibrio in vitro, in vivo*, and Poly IC *in vivo*. (C) *mitf* was DE and showed DAR in its promoter in *Vibrio in vitro*, as well as being among the enriched TFBMs in response to *Vibrio in vitro, in vivo*, and Poly IC *in vivo*. (D) *egr1* was DE and showed DAR in its promoter in *Vibrio in vivo*, as well as being among the enriched TFBMs in response to both *Vibrio in vivo* and *in vitro. stat4* was DE in both *Vibrio* and Poly IC *in vivo* but showed DAR in its promoter only in *Vibrio in vivo*, as well as being among the enriched TFBMs in response to *Vibrio in vivo, in vitro*, and Poly IC *in vivo*.

**Supplementary Fig. S6**. IGV screenshots of the chromatin structure and RNA-seq, ATAC-seq, and ChIP-seq tracks of the *tlr3* gene in the (A) *in vitro* and (B) *in vivo* challenges.

**Supplementary Table S1**. Metadata of the 12 and 18 turbot specimens utilized for the *in vitro* (Fvt) and *in vivo* (Fvv) challenges.

**Supplementary Table S2**. ENA accession IDs to the studies where the raw sequencing files used for the RNA-seq, ATAC-seq, ChIP-seq, and µChIPmentation analysis are allocated.

**Supplementary Table S3**. Summary of the sequencing metrics of the ATAC-seq and ChIP-seq data, processed with the nf-core pipeline.

**Supplementary Table S4**. Differential gene expression results of the *in vitro* challenge stimulated with Poly (I:C) (*P* < 0.05). The symbol in column “log_2_FoldChange” denotes the direction of the differential expression, with negative values (−) referring to genes upregulated in the control condition and positive values referring to genes upregulated in the tested condition (either Poly I:C or *Vibrio*). Column A shows the stable gene IDs used for further analyses.

**Supplementary Table S5**. GO enrichment analysis of the downregulated differentially expressed genes for Poly (I:C) *in vitro* for Biological Process (*P* < 0.05).

**Supplementary Table S6**. Summary of the different GO analyses performed on the lists of common and exclusive differentially expressed genes (DEGs) for each comparison. GO enrichment on Biological Process was chosen as the default analysis (ShinyGO). If no significant enriched GO terms were identified, GO profiling on Biological Process was used (g:Profiler). If neither significant GO enrichment nor profile was found, GO terms for Biological Process for the DEGs in the list were obtained (ENSEMBL).

**Supplementary Table S7**. Immune-related upregulated DEGs showing common responses following *Vibrio* and poly I:C stimulations.

**Supplementary Table S8**. ChIP-seq and ChIPmentation turbot blacklist of low mappability and high input signal regions.

**Supplementary Table S9**. Genome-wide chromatin state distribution of the *in vivo Vibrio* samples.

**Supplementary Table S10**. Differentially accessible regions (DARs) with respect to the corresponding controls for the ATAC-seq assays (*P* < 0.05).

**Supplementary Table S11**. Differential accessibility regions (DARs) and differential histone modification regions (DHMRs) located in promoter regions and integrated with differentially expressed genes (DEGs) for the *in vivo* and *in vitro* challenges stimulated with Poly (I:C) and *Vibrio* (hypergeometric test, *P* < 0.05, Bonferroni correction).

**Supplementary Table S12**. GO enrichment analysis (Biological Process) for the upregulated differentially expressed genes (DEGs) with differential accessibility regions (DARs) and/or differential histone modification regions (DHMRs) in their promoters (*P* < 0.05).

**Supplementary Table S13**. Transcription factor (TF) motif enrichment results for the differential accessibility regions (DARs) and differential histone modification regions (DHMRs) in promoter regions. Only motifs with *P* < 0.05 (Bonferroni correction) and present in more than 10% of the target sequences were considered.

**Supplementary Table S14**. Upregulated TFs with binding motif enrichment predicted on DARs and DHMRs (H3K4me3 and H3K27ac) of active promoters and enhancer regions. The second column shows TFs with promoters overlapping with DARs/DHMRs, while the third column shows TFs that are differentially expressed genes (DEGs). TFs that are in both columns are underlined.

giaf077_Supplemental_Files

giaf077_Authors_Response_To_Reviewer_Comments_original_submission

giaf077_GIGA-D-25-00052_original_submission

giaf077_GIGA-D-25-00052_Revision_1

giaf077_Reviewer_1_Report_original_submission Laura Caquelin -- 3/12/2025

giaf077_Reviewer_2_Report_original_submission Elisabeth Busch-Nentwich -- 4/2/2025

giaf077_Reviewer_3_Report_original_submission Aijun Ma -- 4/3/2025

## Abbreviations

ATAC: assay for transposase-accessible chromatin; ChIP: chromatin immunoprecipitation; DARs: differentially accessible regions; DEGs: differentially expressed genes; DHMRs: differential histone modification regions; FAANG: functional annotation of animal genomes; FDR: false discovery rate; FGF: fibrinogen growth factor; GWAS: genome-wide association studies; GO: Gene Ontology; IFN: interferon; ISG: interferon stimulated genes; IGV: interactive genome browser; MHC: molecular histocompatibility complex; PAMPs: pathogen-associated molecular patterns; PC: principal component; Poly I:C: polyinosinic:polycytidylic acid; PRRs: pattern recognition receptors; SNPs: single-nucleotide polymorphisms; TF: transcription factor; TFBM: transcription factor binding motif; TLR: Toll-like receptor; TSS: transcription starting site.

## Data Availability

All raw RNA-seq, ATAC-seq, and ChIP-seq datasets can be accessed through the ENA repository under accession numbers PRJEB47933, PRJEB47934, and PRJEB57784, respectively. Detailed metadata for the samples and prepared libraries are available in Supplementary Tables S1 and S2, respectively. Detailed experimental protocols are publicly available in the FAANG repository (data.faang.org) and following the URLs facilitated in Supplementary Tables S1 and S2. All additional supporting data are available in the *GigaScience* repository, GigaDB [173].
