# Supplementary material for: Multiomics uncovers the epigenomic and transcriptomic response to viral and bacterial stimulation in turbot
Source: Gigascience. 2025 Jul 15;14:giaf077. doi: 10.1093/gigascience/giaf077 (PMC12263217; doi:10.1093/gigascience/giaf077)
Supplement: giaf077_Supplemental_Files [file giaf077_supplemental_files.zip › Supplementary Figures v2.pdf]

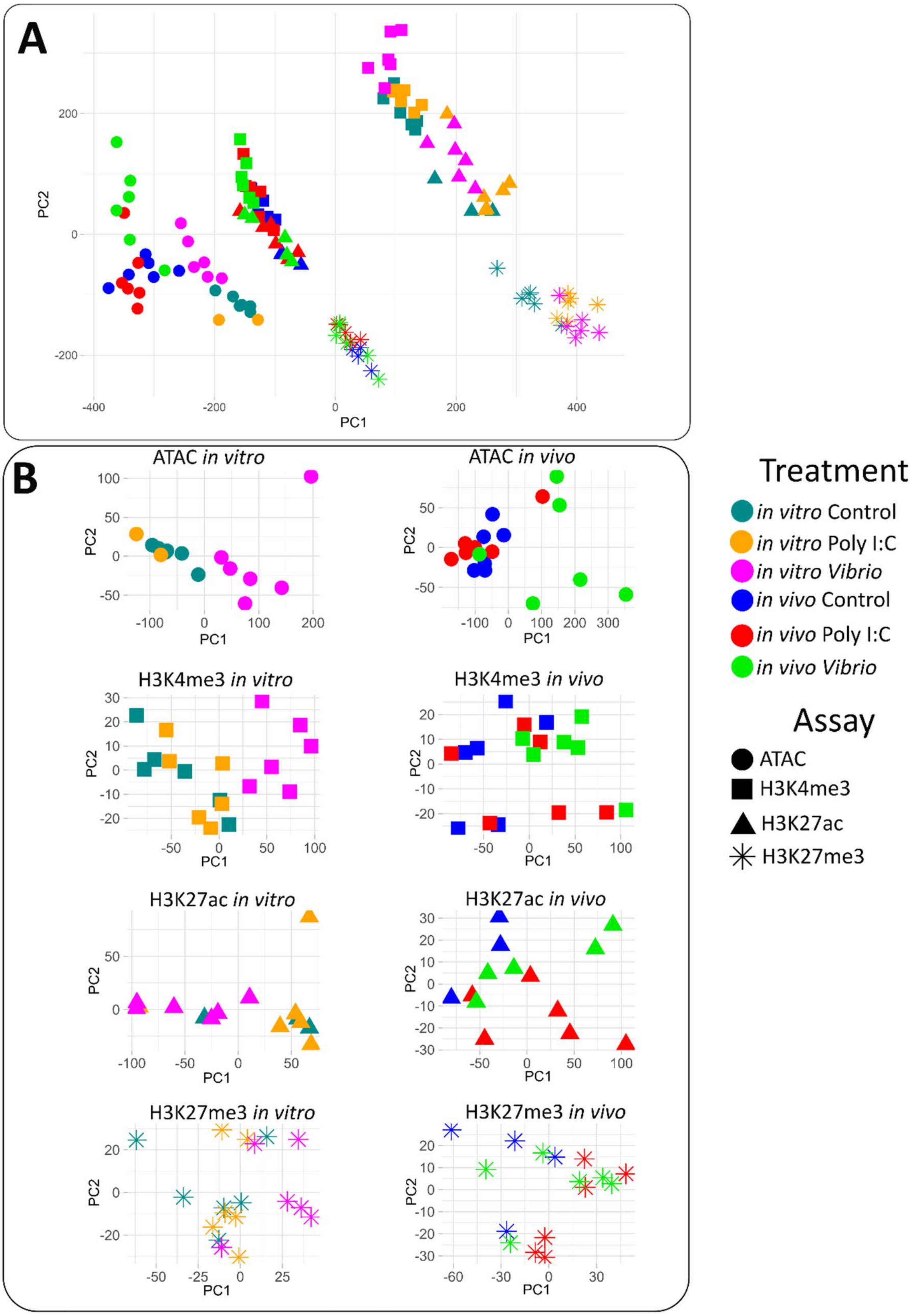

Supplementary Figure 1. A) Full and **B)** Separate PCAs for each assay and stimulation of the *in vivo* and *in vitro* epigenomic response to stimulation with poly I:C and *Vibrio* for the open chromatin ATAC-Seq and histone ChIP-Seq (H3K4me3, H3K27ac, H3K27me3) samples.

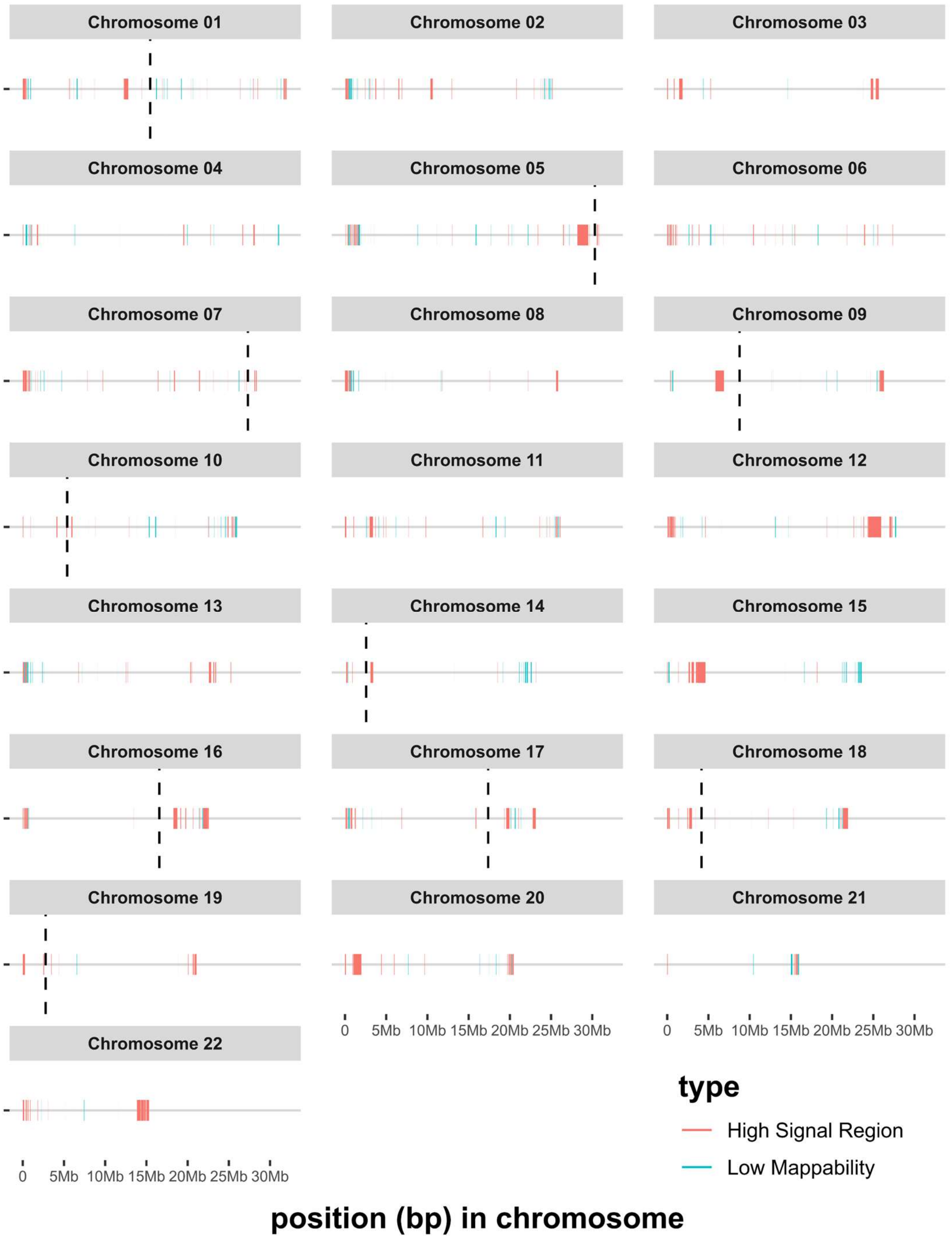

Supplementary Figure 2. Genomic distribution of the ChIP-Seq high signal regions (pink) and low mappability regions (blue) of the turbot genome included in the blacklist for each of the 22 chromosomes. The dotted lines represent the approximate centromere positions, based on the microsatellite analysis of Martínez et al., 2008.

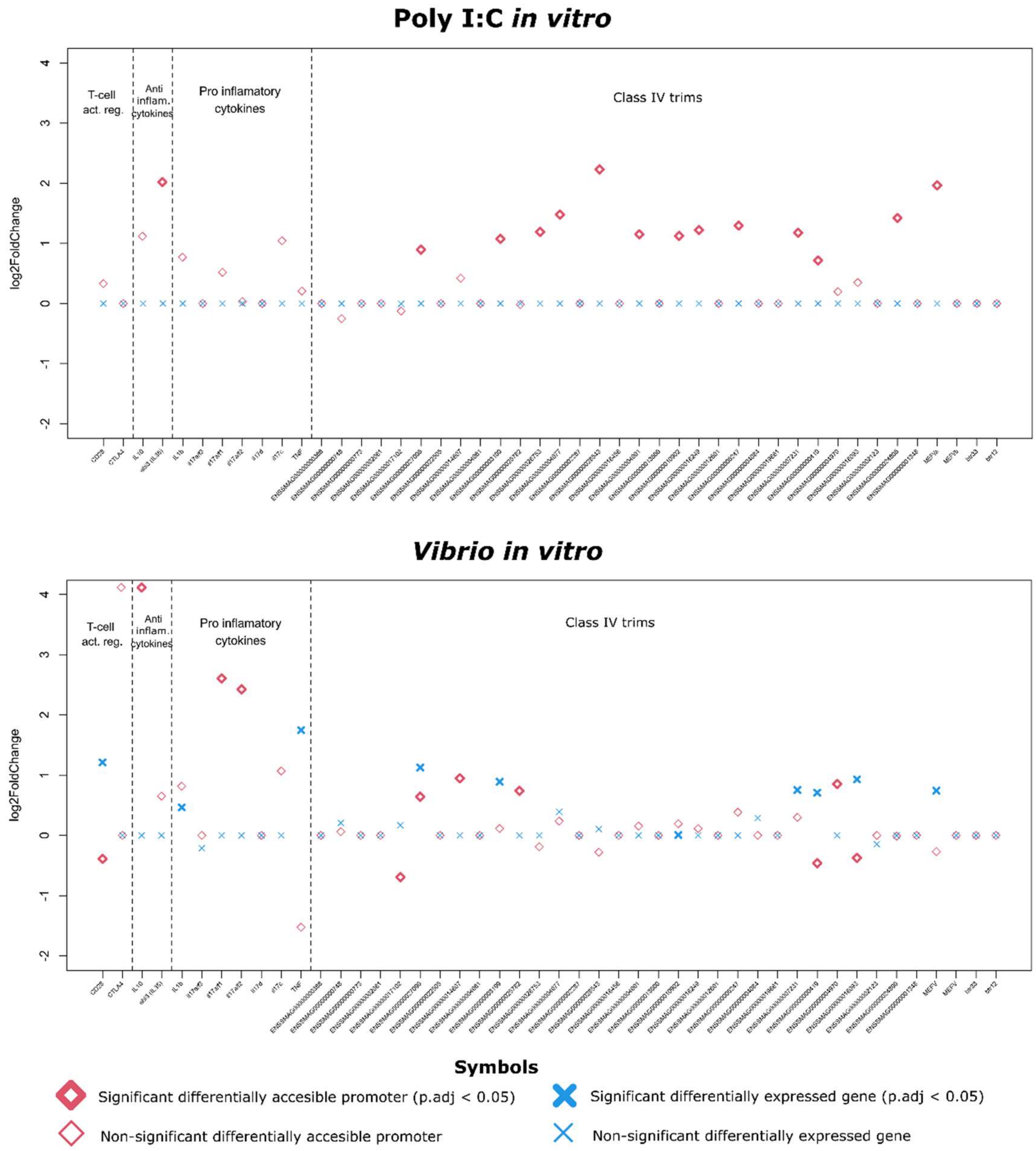

Supplementary figure 3A. Promoter accessibility and gene expression (log2FC, ordinates) in the *in vitro* assays of key immune genes related to i) regulation of activation of T-cell immune response (*cd28* and *ctla4*); ii) anti-inflammatory (*il10, il35 subunit ebi3*) and iii) pro-inflammatory cytokines (*il1b*, *il17a/f1*, *il17a/f2*, *il17a/f3*, *il17c*, *il17d* and *tnf*) and iv) all class IV *trim* genes annotated in the turbot genome.

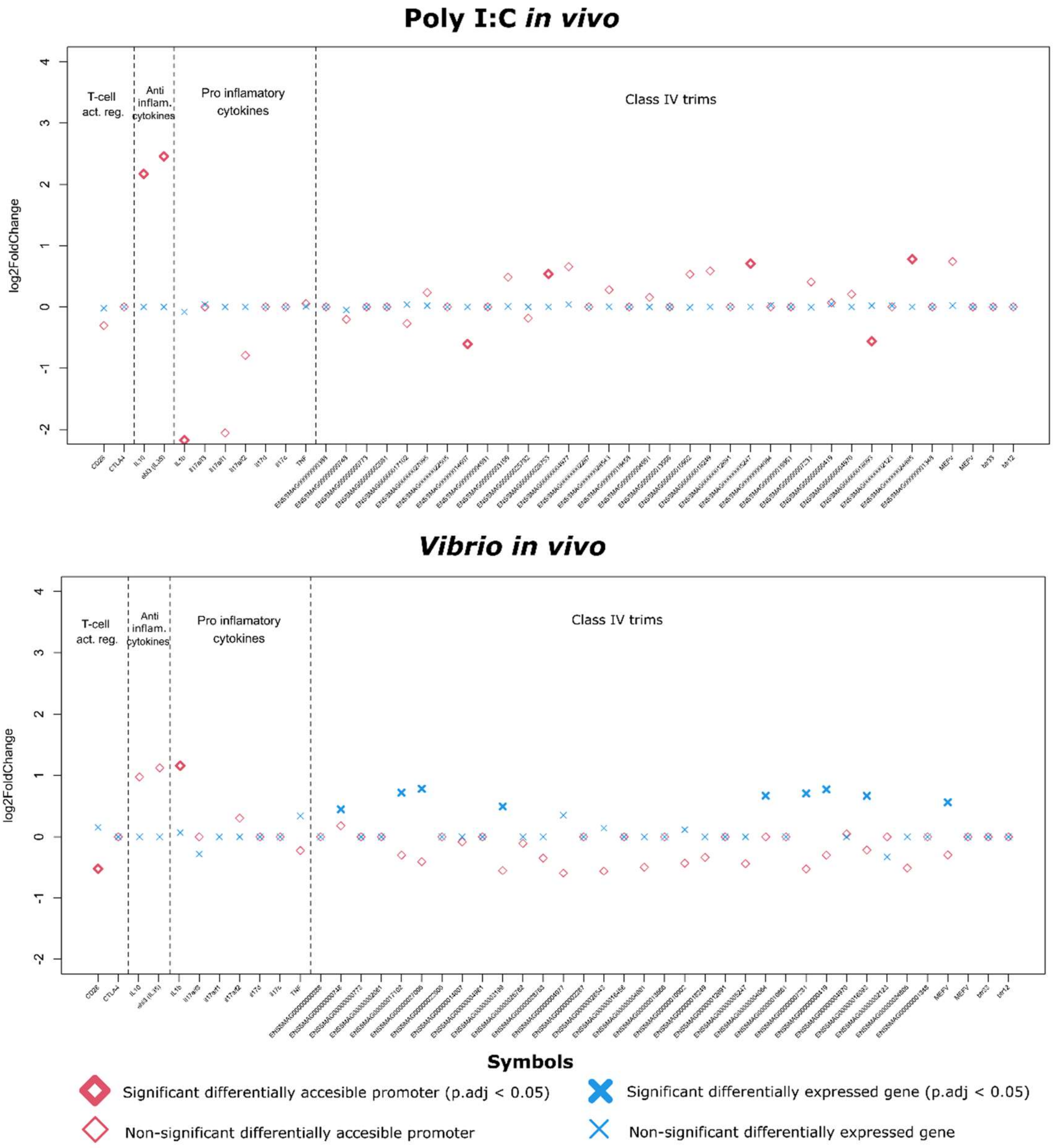

Supplementary figure 3B. Promoter accessibility and gene expression (log2FC, ordinates) in the *in vivo* assays of key immune genes related to i) regulation of activation of T-cell immune response (*cd28* and *ctla4*); ii) anti-inflammatory (*il10, il35 subunit ebi3*) and iii) pro-inflammatory cytokines (*il1b*, *il17a/f1*, *il17a/f2*, *il17a/f3*, *il17c*, *il17d* and *tnf*) and iv) all class IV *trim* genes annotated in the turbot genome.

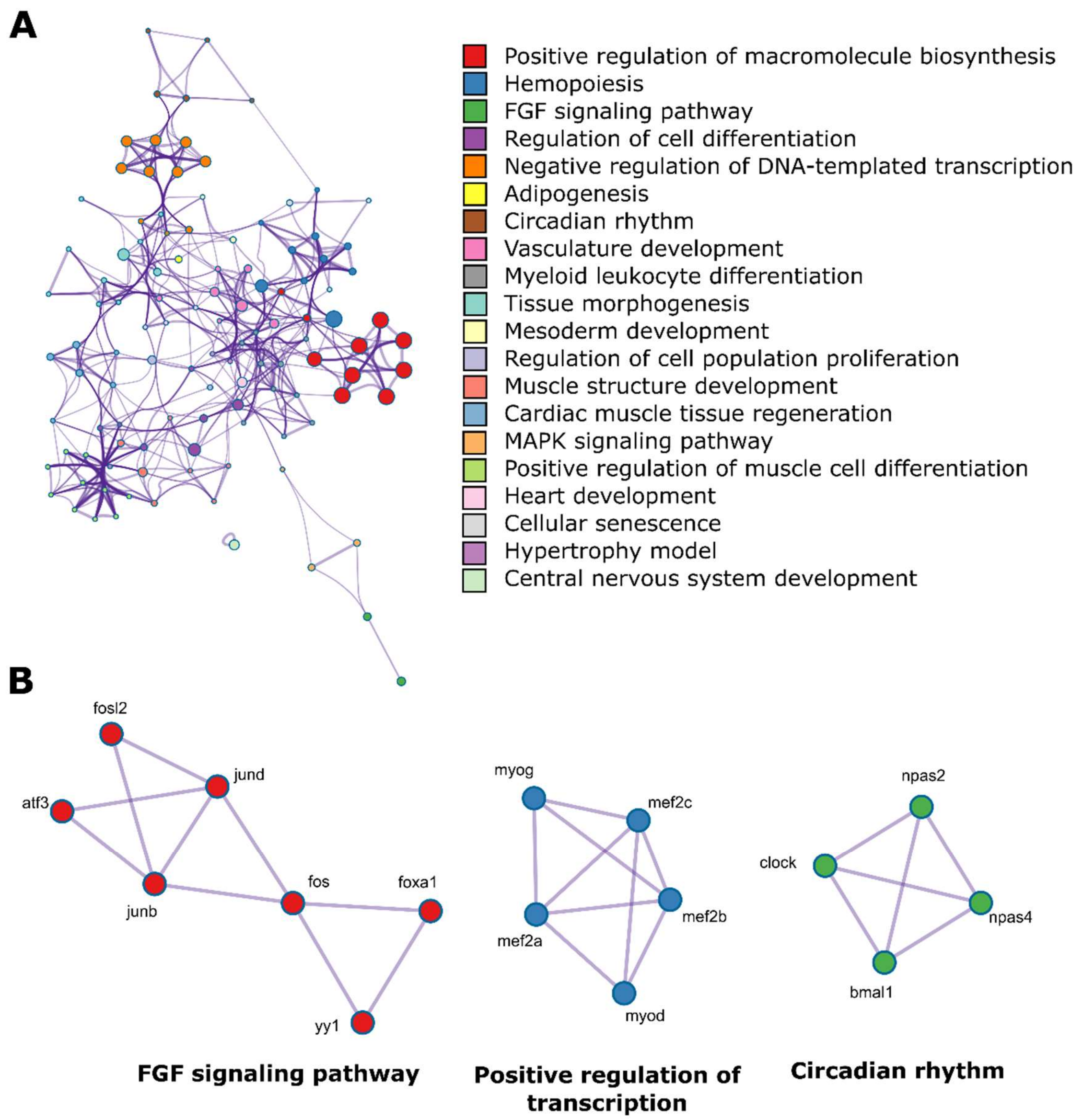

Supplementary figure 4. Functional interconnection of active TFs detected in response to the different stimulants. **A)** Ontology enrichment clusters of upregulated TFs showing promoter DARs / DHMRs with TFBMs enriched in promoter or enhancer DARs / DHMRs of DEGs detected in *Vibrio in vivo* stimulation. **B)** Protein-protein interaction MCODE network of upregulated TFs induced by *Vibrio in vivo* stimulation. Each cluster is coloured with the most statistically significant term among terms that cluster together. The size of each term is given by -log10p, and the stronger the similarity among nearby terms, the thicker the edges between them.

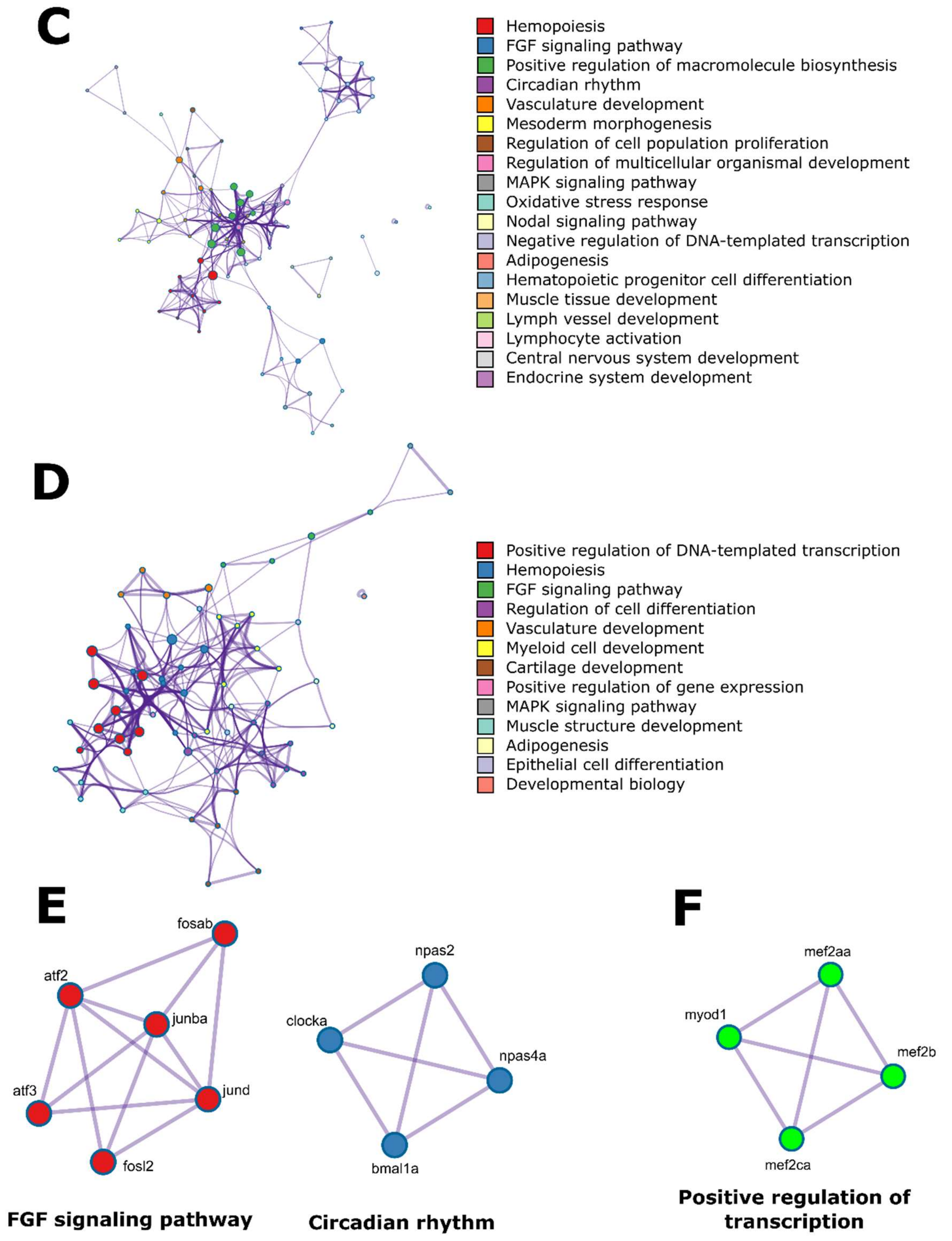

Supplementary figure 4. (cont) Functional interconnection of active TFs detected in response to the different stimulants. **C)** and **D)** Ontology enrichment clusters of upregulated TFs showing promoter DARs / DHMRs with TFBMs enriched in promoter or enhancer DARs / DHMRs of DEGs detected in *Vibrio in vitro* and Poly I:C *in vivo* stimulation, respectively. **E)** and **F)** Protein-protein interaction MCODE network of upregulated TFs induced by *Vibrio in vitro* and Poly I:C *in vivo* stimulation, respectively.

arnt (HIF1B)
Chromosome 1: 19,174,451-19,183,553

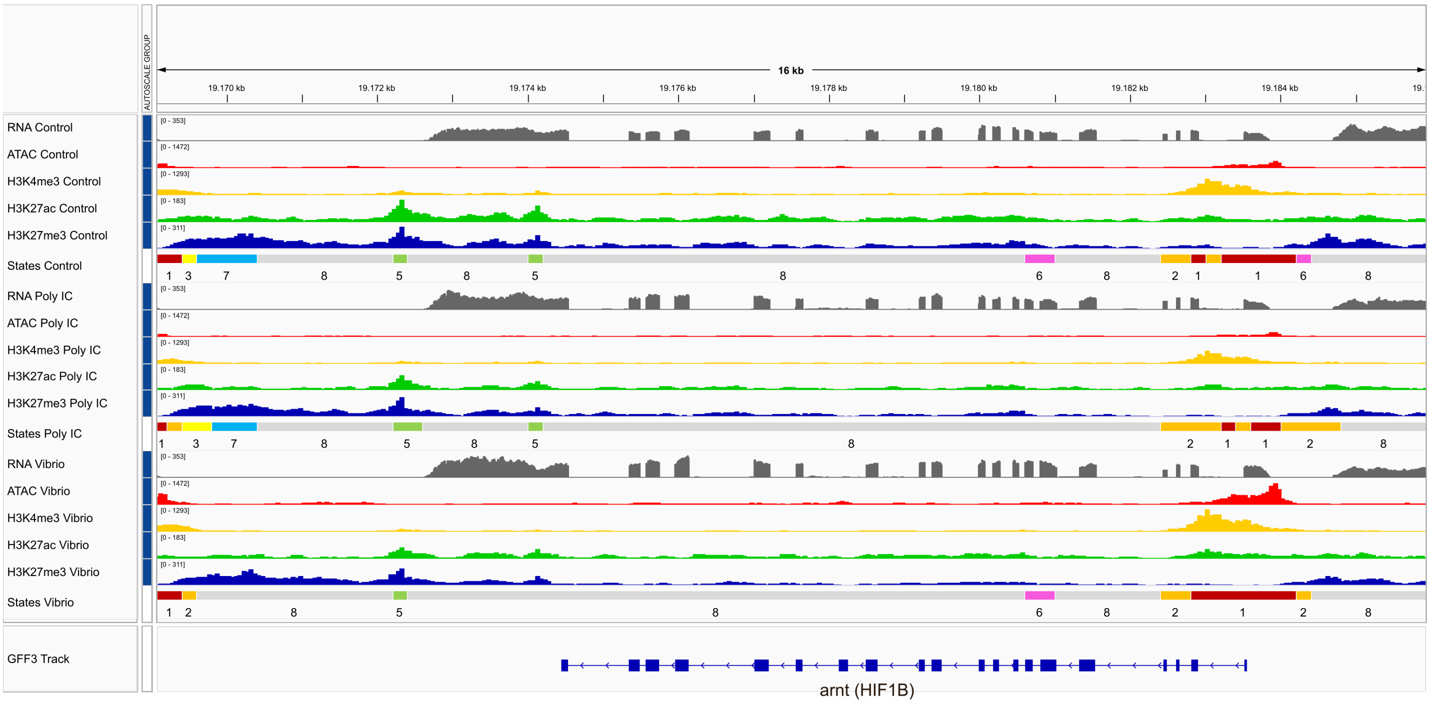

irf8
Chromosome 5: 14,893,961-14,898,170

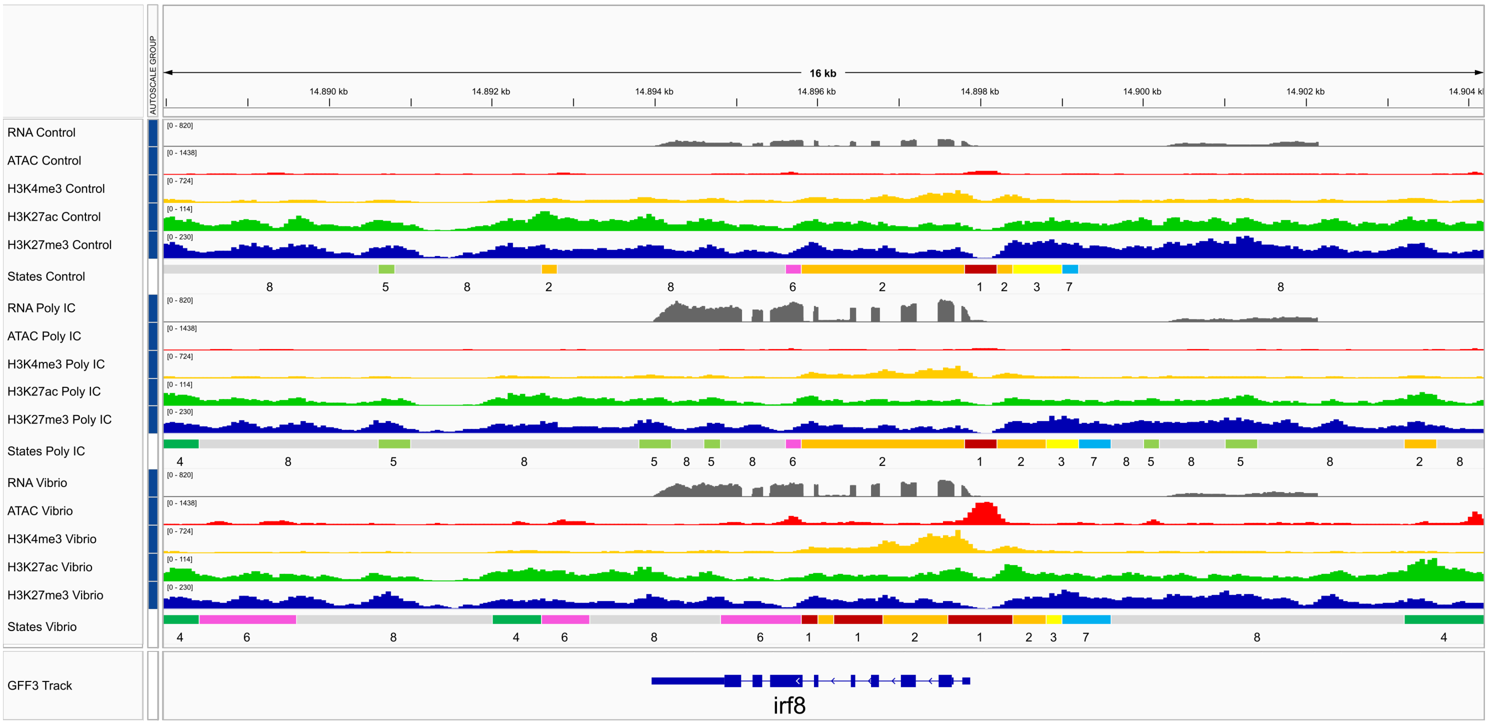

1	Strongly active promoter / transcript	3	Bivalent / poised TSS	5	Bivalent / poised enhancer	7	Repressed Polycomb
2	Flanking active TSS without ATAC	4	Strong active enhancer	6	ATAC island	8	Low signal

Supplementary Figure 5A. IGV screenshots of the chromatin structure and RNA-Seq, ATAC-Seq and ChIP-Seq tracks of different transcription factor coding genes, selected due to them i) being differentially expressed (DE) in at least one of the conditions; ii) having differentially accesible regions (DAR) or differential histone modification regions (DHMR) in at least one of the conditions and iii) being among the TFBM enriched in response to at least one of the conditions. Each chromatin state model annotation is located in the table bellow the IGV screenshots. *arnt* was DE and showed DAR and H3K4me3-DAR in its promoter in *Vibrio in vitro*, as well as being among the TFBM enriched in response to both *Vibrio in vitro* and *in vivo*. *irf8* was DE in both *Vibrio* and Poly IC *in vitro*, but only showed DAR in its promoter in *Vibrio in vitro*, as well as being among the enriched TFBM in response to *Vibrio in vitro*, *in vivo* and Poly IC *in vivo*.

bhlhe40a
Chromosome 6: 14,702,766-14,705,188

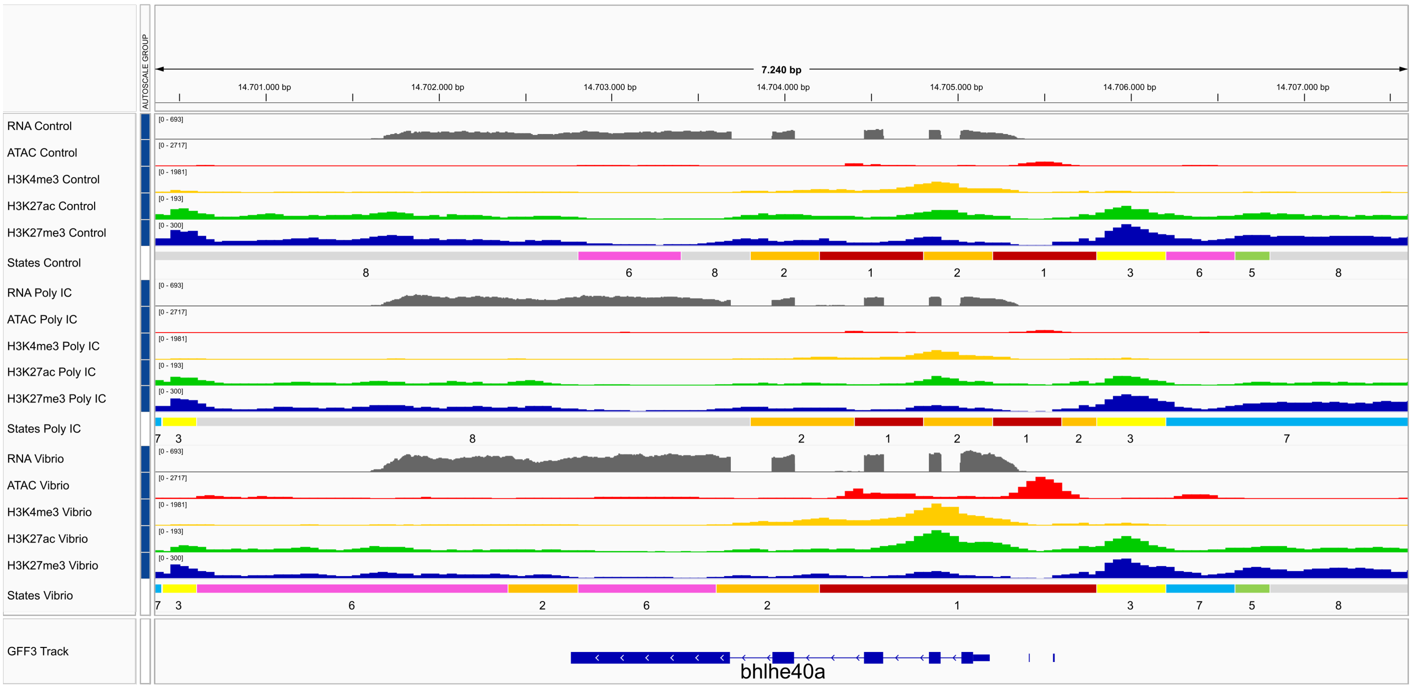

bhlhe40b
Chromosome 6: 21,238,776-21,240,855

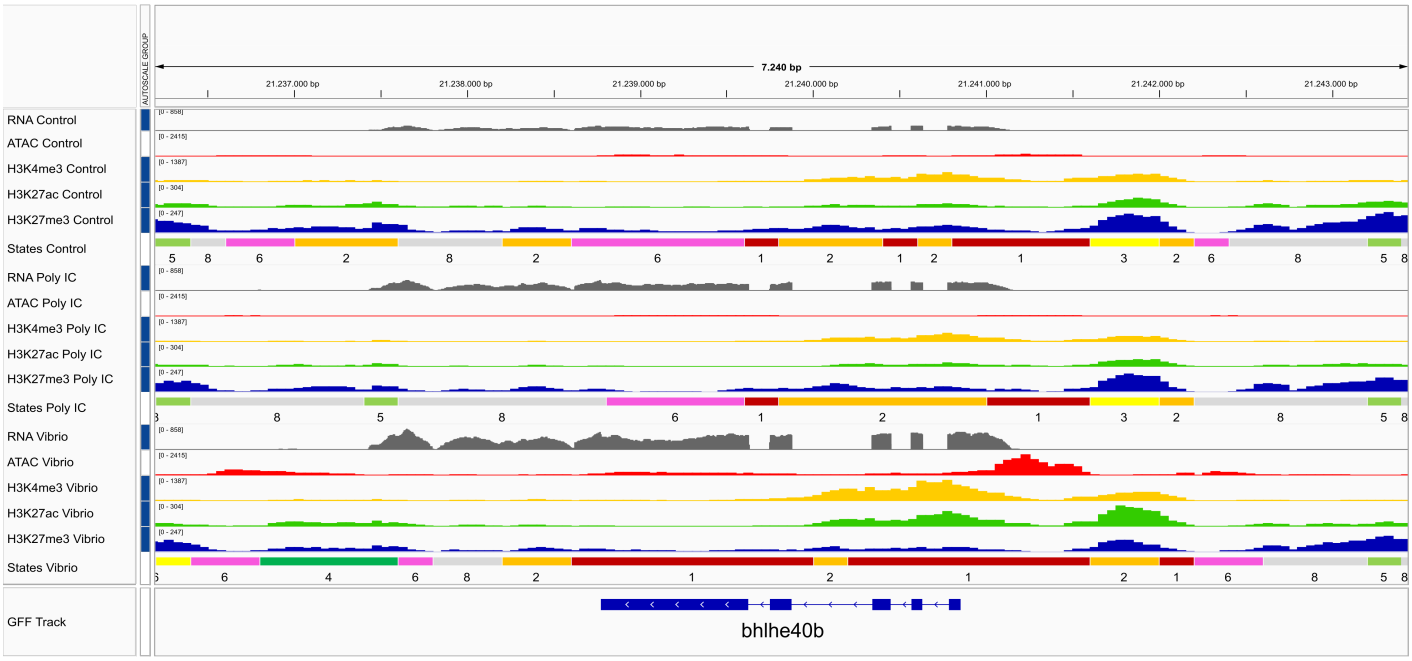

1	Strongly active promoter / transcript	3	Bivalent / poised TSS	5	Bivalent / poised enhancer	7	Repressed Polycomb
2	Flanking active TSS without ATAC	4	Strong active enhancer	6	ATAC island	8	Low signal

Supplementary Figure 5B. (Cont.) *bhlhe40a* and *bhlhe40b* were DE and showed DAR in its promoter in *Vibrio in vitro*, as well as being among the enriched TFBM in response to *Vibrio in vitro*, i*n vivo*, and Poly IC *in vivo*.

mitf
Chromosome 11: 24,592,124-24,619,150

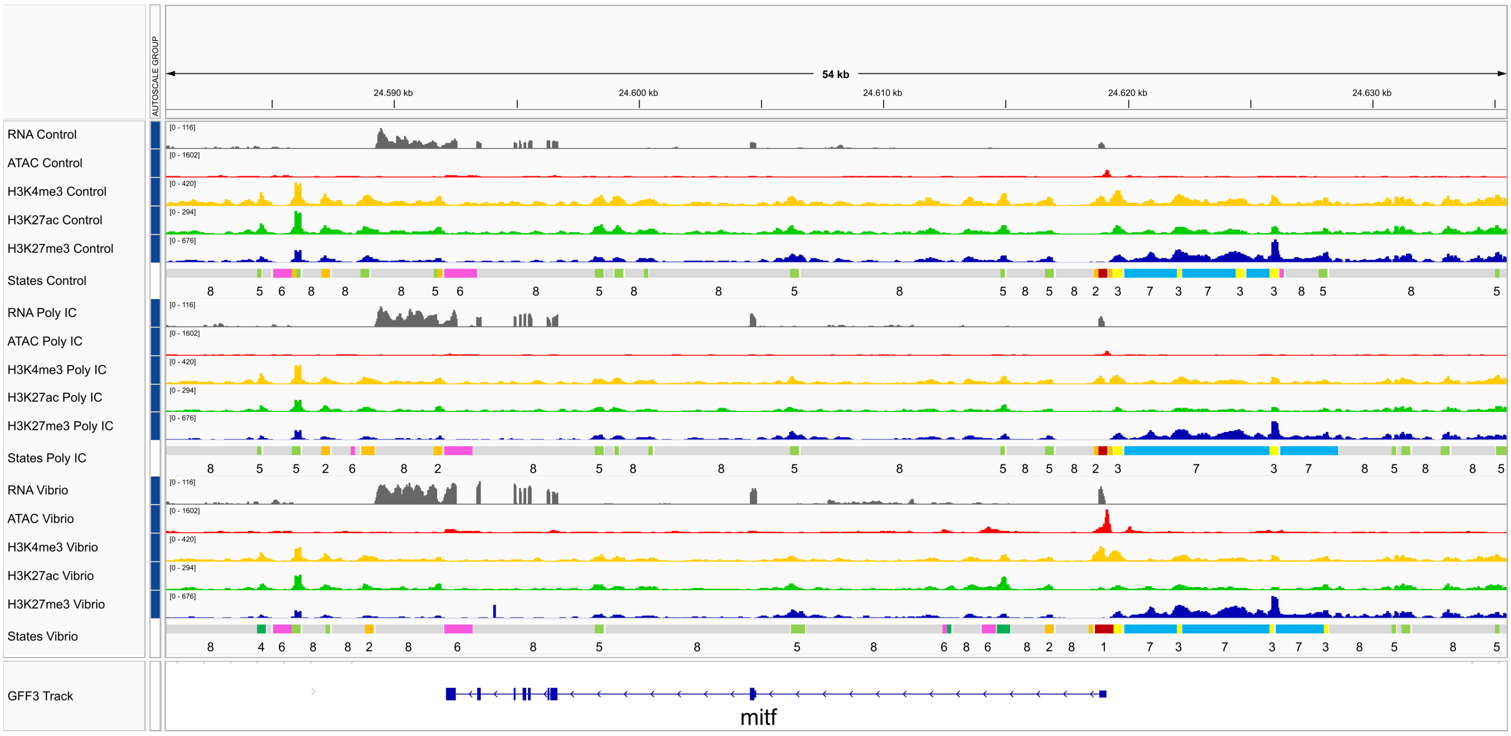

1	Strongly active promoter / transcript	3	Bivalent / poised TSS	5	Bivalent / poised enhancer	7	Repressed Polycomb
2	Flanking active TSS without ATAC	4	Strong active enhancer	6	ATAC island	8	Low signal

Supplementary Figure 5C. (Cont.) *mitf* was DE and showed DAR in its promoter in *Vibrio in vitro*, as well as being among the enriched TFBM in response to *Vibrio in vitro*, *in vivo*, and Poly IC *in vivo*.

egr1

Chromosome 13: 14,094,894-14,099,153

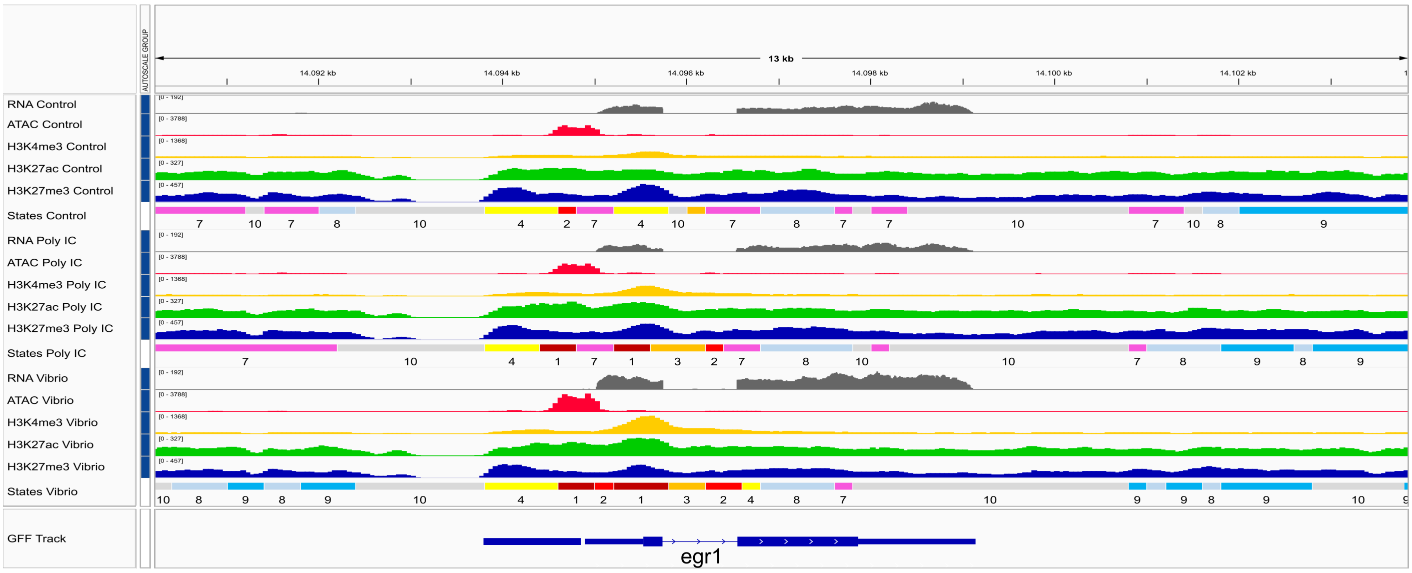

stat4

Chromosome 14: 389,657-402,701

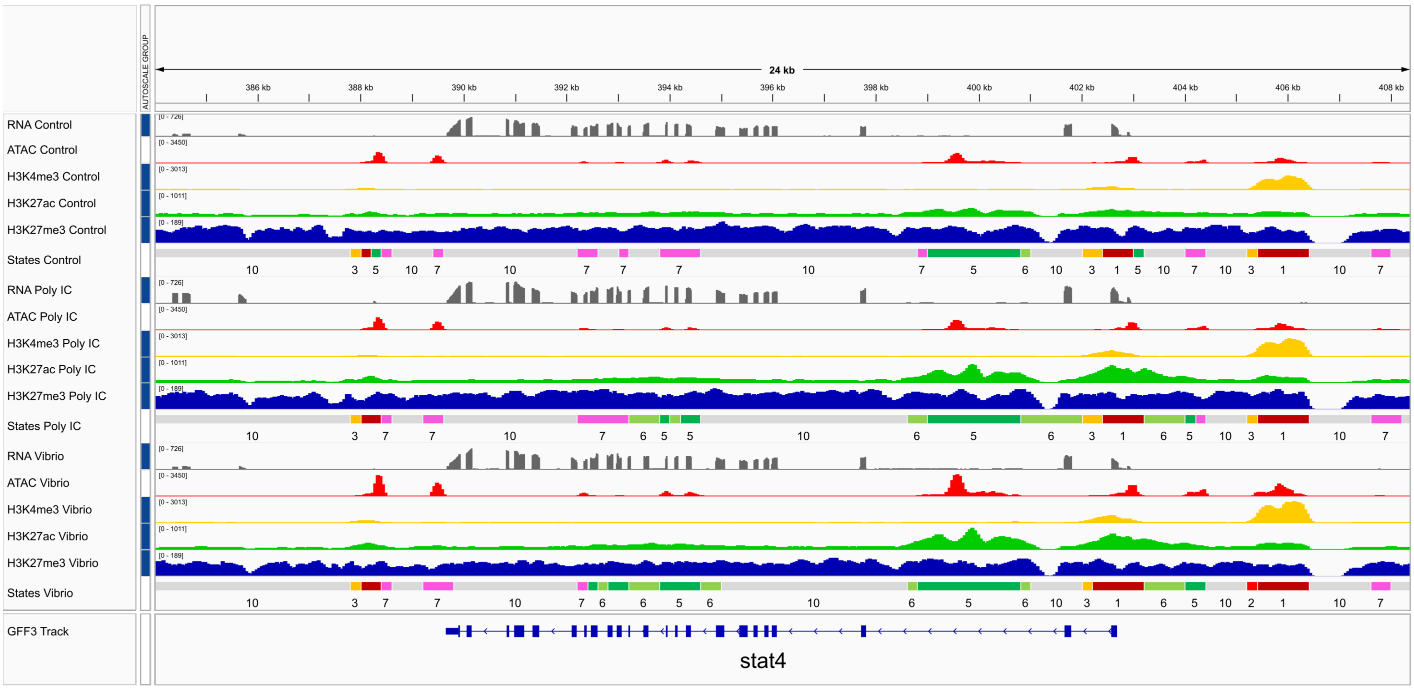

1	Strongly active promoter / transcript	3	Flanking active TSS without ATAC	5	Strong active enhancer	7	ATAC island	9	Repressed Polycomb
2	Weak active promoter / transcript	4	Bivalent/poised TSS	6	Weak active enhancer	8	Weak Repressed Polycomb	10	Low signal

Supplementary Figure 5D. (Cont.) *egr1* was DE and showed DAR in its promoter in *Vibrio in vivo*, as well as being among the enriched TFBMs in response to both *Vibrio in vivo* and i*n vitro*. *stat4* was DE in both *Vibrio* and Poly IC *in vivo*, but showed DAR in its promoter only in *Vibrio in vivo*, as well as being among the enriched TFBM in response to *Vibrio in vivo*, *in vitro*, and Poly IC *in vivo*.

bcl11a
Chromosome 15: 16,712,975-16,756,991

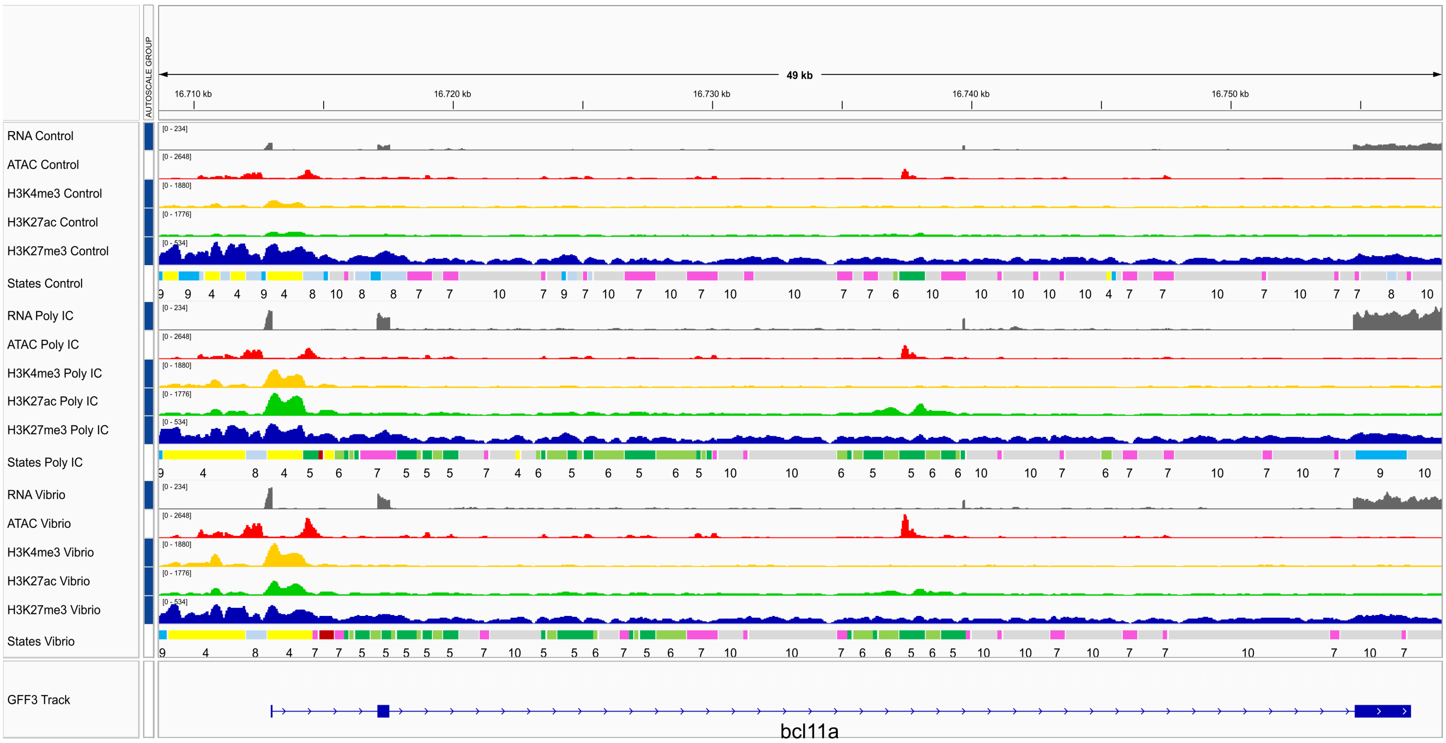

meis1b
Chromosome 15: 18,783,030-18,864,551

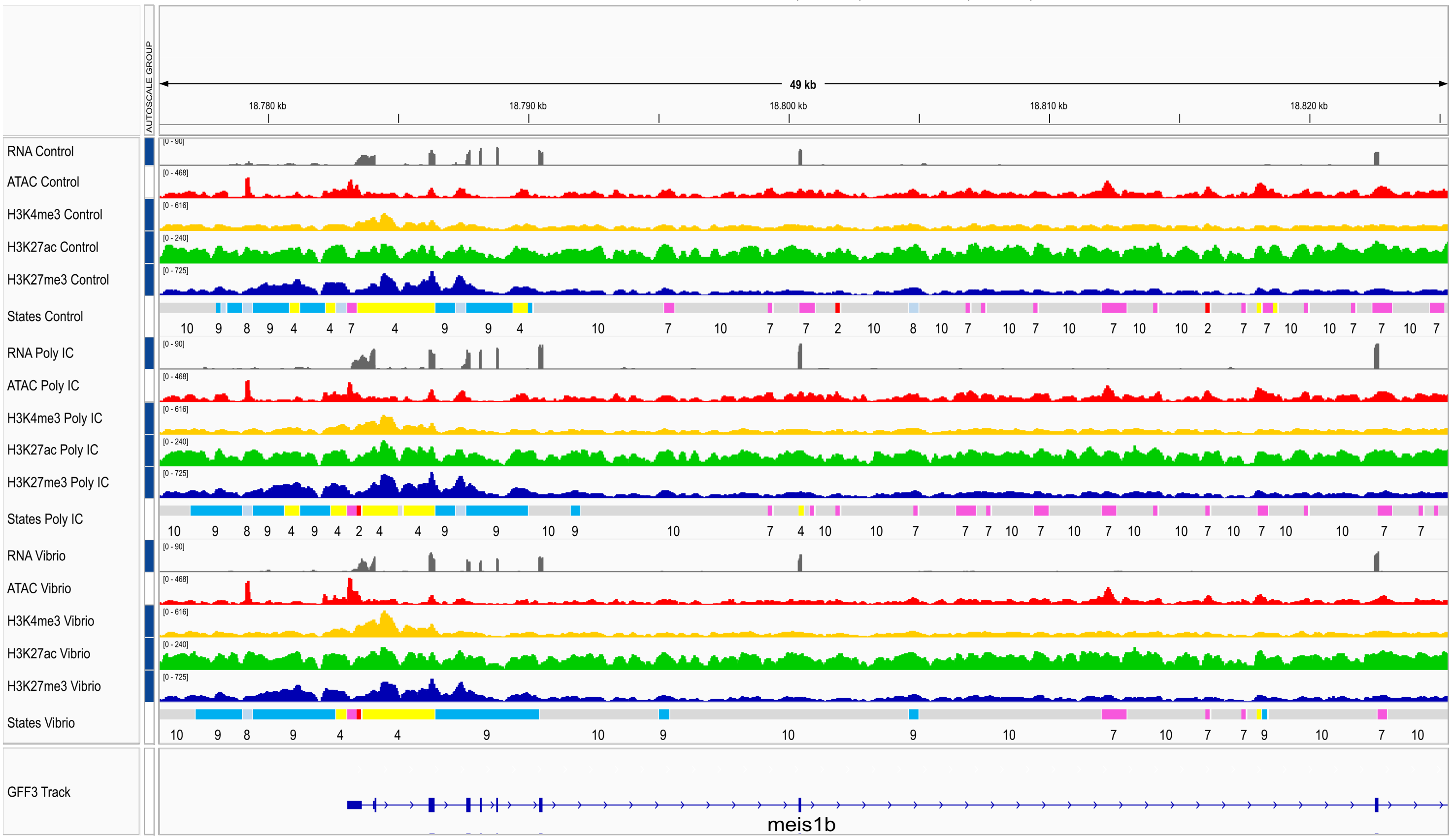

1	Strongly active promoter / transcript	3	Flanking active TSS without ATAC	5	Strong active enhancer	7	ATAC island	9	Repressed Polycomb
2	Weak active promoter / transcript	4	Bivalent/poised TSS	6	Weak active enhancer	8	Weak Repressed Polycomb	10	Low signal

Supplementary Figure 3E. (Cont.) *bcl11a* was DE in both Vibrio and Poly IC *in vivo*, but showed DAR in its promoter only in *Vibrio in vivo*, as well as being among the enriched TFBM is response to both *Vibrio in vivo* and *in vitro*. *meis1b* was DE and showed DAR its promoter in *Vibrio in vivo*, as well as being among the enriched TFBMs in response to both *Vibrio in vivo* and *in vitro*.

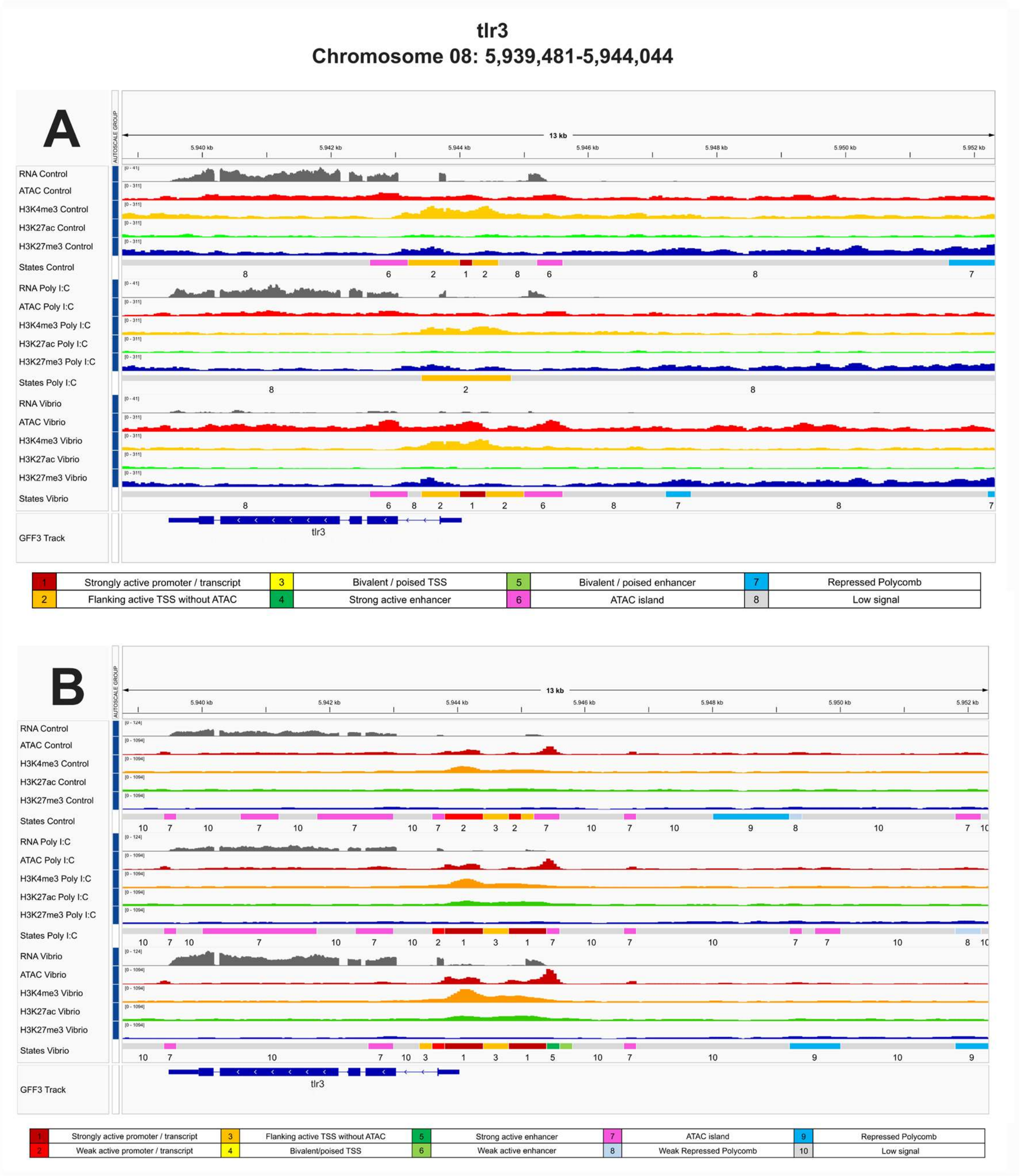

Supplementary figure 6. IGV screenshots of the chromatin structure and RNA-Seq, ATAC-Seq and ChIP-Seq tracks of the *tlr3* gene in the A) *in vitro* and B) *in vivo* challenges.
