# Supplementary material for: Multiomics uncovers the epigenomic and transcriptomic response to viral and bacterial stimulation in turbot
Source: Gigascience. 2025 Jul 15;14:giaf077. doi: 10.1093/gigascience/giaf077 (PMC12263217; doi:10.1093/gigascience/giaf077)
Supplement: giaf077_Supplemental_Files [file giaf077_supplemental_files.zip › Supplementary_table_7_NEW.docx]

**Supplementary table X.** Immune-related upregulated DEGs showing common responses following *Vibrio* and poly I:C stimulations.

| **Comparison** | **Differentially expressed genes (DEGs)** |
| --- | --- |
| *In vitro* – common upregulated | b2m-like, btk, c2-like, ccl19-like, cd226, cfb-like, cxcl11.5, cxcl19, cxcl1-like, **erap1b**, etv7, fas, faslg, gabrr1-like, hsp90ab1, **il15ra**, irf7, irf8, lyg-like, **nmi**, **nod2**, nol9, ppbp-like, psmb9a, psmb10, pycard, satb1b, smyd5, **socs1a**, **socs1b**, stat4, **tapbp.2**, **tapbpl**, tnfsf14, **traf2**, wdr43, zap70 |
| *In vivo* – common upregulated | *prmt1, prmt3, prmt5, prmt7* |
| Poly I:C – common upregulated | *ackr4a, b2m-like, c2-like, ccl19-like, cfb-like, cxcl11.5, cxcl19, dram1,* ***erap1b****, etv7, fas, faslg, hsp90ab1, ifih1,* ***il15ra****, irf8, lyg-like,* ***nmi****,* ***nod2****, nol9, psmb9a, psmb10, pycard, satb1b, smchd1, smyd5,* ***socs1a****,* ***socs1b****, sting1,* ***tapbp.2***, ***tapbpl****, tnfsf14, usp4, wdr43* |
| *Vibrio* – common upregulated | *prmt1, prmt3, prmt5, prmt7* |
