# Supplementary material for: Multiomics uncovers the epigenomic and transcriptomic response to viral and bacterial stimulation in turbot
Source: Gigascience. 2025 Jul 15;14:giaf077. doi: 10.1093/gigascience/giaf077 (PMC12263217; doi:10.1093/gigascience/giaf077)
Supplement: giaf077_GIGA-D-25-00052_original_submission [file giaf077_giga-d-25-00052_original_submission.pdf]

GigaScience

Multiomics uncovers the epigenomic and transcriptomic response to viral and bacterial stimulation in turbot

--Manuscript Draft--

**Manuscript Number:**	GIGA-D-25-00052
**Full Title:**	Multiomics uncovers the epigenomic and transcriptomic response to viral and bacterial stimulation in turbot
**Article Type:**	Research
**Funding Information:**	Horizon 2020 Framework Programme (817923) — Not applicable
	Consellería de Cultura, Educación e Ordenación Universitaria, Xunta de Galicia (ED431C 2022/33) — Not applicable
	Consellería de Cultura, Educación e Ordenación Universitaria, Xunta de Galicia (ED481A-2020/119) — Mr. Oscar Aramburu
	Consellería de Cultura, Educación e Ordenación Universitaria, Xunta de Galicia (ED481A-2020/491430) — Mrs Paula Rodríguez-Villamayor
	BBSRC Institutional Strategic Programme (BBS/E/D/10002070) — Not applicable
	BBSRC Institutional Strategic Programme (BBS/E/D/20002174) — Not applicable
	BBSRC Institutional Strategic Programme (BBS/E/RL/230001B) — Not applicable
	BBSRC Institutional Strategic Programme (BBS/E/RL/230002B) — Not applicable

Abstract:

Background

Uncovering the epigenomic regulation of immune response is essential for a comprehensive understanding of host defence mechanisms, though it remains poorly investigated in farmed fish.

Results

We report the first annotation of the innate immune regulatory response in the turbot genome (Scophthalmus maximus), integrating RNA-Seq with ATAC-Seq and ChIP-Seq (H3K4me3, H3K27ac, and H3K27me3) data from head kidney (in vivo) and primary leukocyte cultures (in vitro) 24 hours post-stimulation with viral (poly I:C) and bacterial (inactive Vibrio anguillarum) mimics. Among the 8,797 differentially expressed genes (DEGs), we observed enrichment of transcriptional activation pathways in response to Vibrio and immune pathways—including interferon-stimulated genes—for poly I:C. We identified notable differences in chromatin accessibility (20,617 in vitro, 59,892 in vivo) and H3K4me3-bound regions (11,454 in vitro, 10,275 in vivo) between stimulations and controls. Overlap of DEGs with promoters showing differential accessibility or histone mark binding revealed significant coupling of the transcriptome and chromatin state. DEGs with activation marks in their promoters were enriched for similar functions to the global DEG set, but not always, suggesting key regulatory genes being in a poised state. Active promoters and putative enhancers were enriched in specific transcription factor binding motifs, many common to viral and bacterial responses. An in-depth analysis of chromatin state surrounding key DEGs encoding transcription factors was also performed to understand turbot immune response.

Conclusions

This multi-omics investigation provides an improved understanding of the epigenomic basis of turbot immune response and offers novel functional genomic information,

Powered by Editorial Manager® and ProduXion Manager® from Aries Systems Corporation

	which can be leveraged for disease resistance selective breeding.
Corresponding Author:	Paulino Martinez<br>University of Santiago de Compostela: Universidade de Santiago de Compostela<br>Lugo, SPAIN
Corresponding Author Secondary Information:	
Corresponding Author's Institution:	University of Santiago de Compostela: Universidade de Santiago de Compostela
Corresponding Author's Secondary Institution:	
First Author:	Oscar Aramburu, Ph.D
First Author Secondary Information:	
Order of Authors:	Oscar Aramburu, Ph.D
	Belén Gómez-Pardo
	Paula Rodríguez-Villamayor
	Andrés Blanco-Hortas
	Jesús Lamas
	Pooran Dewari
	Diego Perojil-Morata
	Pierre Boudinot
	Daniel J. Macqueen
	Carmen Bouza
	Paulino Martinez
Order of Authors Secondary Information:	
Additional Information:	
Question	Response
Are you submitting this manuscript to a special series or article collection?	No
**Experimental design and statistics**<br><br>Full details of the experimental design and statistical methods used should be given in the Methods section, as detailed in our Minimum Standards Reporting Checklist. Information essential to interpreting the data presented should be made available in the figure legends.<br><br>Have you included all the information requested in your manuscript?	Yes
**Resources**	Yes

Powered by Editorial Manager® and ProduXion Manager® from Aries Systems Corporation

A description of all resources used, including antibodies, cell lines, animals and software tools, with enough information to allow them to be uniquely identified, should be included in the Methods section. Authors are strongly encouraged to cite Research Resource Identifiers (RRIDs) for antibodies, model organisms and tools, where possible.<br><br>Have you included the information requested as detailed in our Minimum Standards Reporting Checklist?	
**Availability of data and materials**<br><br>All datasets and code on which the conclusions of the paper rely must be either included in your submission or deposited in publicly available repositories (where available and ethically appropriate), referencing such data using a unique identifier in the references and in the "Availability of Data and Materials" section of your manuscript.<br><br>Have you have met the above requirement as detailed in our Minimum Standards Reporting Checklist?	No
If not, please give reasons for any omissions below.<br><br>as follow-up to "**Availability of data and materials**<br><br>All datasets and code on which the conclusions of the paper rely must be either included in your submission or deposited in publicly available repositories (where available and ethically appropriate), referencing such data using a unique identifier in the references and in the "Availability of Data and Materials" section of your manuscript.	All raw RNA-Seq, ATAC-Seq and ChIP-Seq datasets can be accessed through the ENA repository under accession numbers PRJEB47933, PRJEB47934 and PRJEB57784, respectively. Detailed metadata for the samples and prepared libraries are available in Supplementary tables 1 and 2, respectively. Detailed experimental protocols are publicly available in the FAANG repository (data.faang.org) and following the URLs facilitated in Supplementary tables 1 and 2. At this stage, some of the code and scripts associated with this study are not yet publicly available, as we are awaiting editorial feedback on the suitability of our manuscript for GigaScience. Upon confirmation of its fitness for revision for publication, we will make all relevant code and scripts publicly accessible in accordance with the journal's data and code sharing policies.

Powered by Editorial Manager® and ProduXion Manager® from Aries Systems Corporation

Have you have met the above requirement as detailed in our Minimum Standards Reporting Checklist?<br><br>"	
GigaScience has policies and guidelines in place for the use of generative AI-writing tools such as ChatGPT. If you have used such writing tools to assist with writing the manuscript this must be declared and cited in the text. Authors should not list AI-writing tools and other AI-assisted technologies as an author or co-author and should acknowledge that they are fully responsible for text generated or refined by AI-writing tools.<p><br><br>A summary of use (particularly in the introduction or among methods) needs to be included at the end of the paper, and the outputs should also be included as a supplementary file hosted in GigaDB or other open repositories. Please read our guidelines for more information. <p><br><br>By submitting to GigaScience, you are aware of the journal's AI-writing tools policy, and if you have declared use of such tools below, you have acknowledged this where appropriate in your manuscript and have made a summary of use and outputs available. <p><br><b>AI-assisted writing tools have been used in the preparation of this manuscript?	No

Powered by Editorial Manager® and ProduXion Manager® from Aries Systems Corporation

Manuscript

Click here to access/download;Manuscript;Manuscript_no_figures_tables.do

Multiomics uncovers the epigenomic and transcriptomic response to viral and bacterial stimulation in turbot

Oscar Aramburu [1]*, Belén Gómez-Pardo[1], Paula Rodríguez-Villamayor[1], Andrés Blanco-Hortas[1], Jesús Lamas[1], Pooran Dewari[2], Diego Perojil-Morata[2], Pierre Boudinot[3], Daniel J. Macqueen[2], Carmen Bouza[1], Paulino Martínez[1*]

[1] University of Santiago de Compostela, Spain

[2] The Roslin Institute and Royal (Dick) School of Veterinary Studies, University of Edinburgh, Easter Bush Campus, UK

[3] Université Paris-Saclay, INRAE, UVSQ, VIM, Jouy-en-Josas, 78350, France.

* Corresponding authors

ABSTRACT

Background:
Uncovering the epigenomic regulation of immune response is essential for a comprehensive understanding of host defence mechanisms, though it remains poorly investigated in farmed fish.

Results:
We report the first annotation of the innate immune regulatory response in the turbot genome (*Scophthalmus maximus*), integrating RNA-Seq with ATAC-Seq and ChIP-Seq (H3K4me3, H3K27ac, and H3K27me3) data from head kidney (in vivo) and primary leukocyte cultures (in vitro) 24 hours post-stimulation with viral (poly I:C) and bacterial (inactive *Vibrio anguillarum*) mimics. Among the 8,797 differentially expressed genes (DEGs), we observed enrichment of transcriptional activation pathways in response to *Vibrio* and immune pathways—including interferon-stimulated genes—for poly I:C. We identified notable differences in chromatin accessibility (20,617 in vitro, 59,892 in vivo) and H3K4me3-bound regions (11,454 in vitro, 10,275 in vivo) between stimulations and controls. Overlap of DEGs with promoters showing differential accessibility or histone mark binding revealed significant coupling of the transcriptome and chromatin state. DEGs with activation marks in their promoters were enriched for similar functions to the global DEG set, but not always, suggesting key regulatory genes being in a poised state. Active promoters and putative enhancers were enriched in specific transcription factor binding motifs, many common to viral and bacterial responses. An in-depth analysis of chromatin state surrounding key DEGs encoding transcription factors was also performed to understand turbot immune response.

Conclusions:
This multi-omics investigation provides an improved understanding of the epigenomic basis of turbot immune response and offers novel functional genomic information, which can be leveraged for disease resistance selective breeding.

Keywords: turbot, immune response, epigenomics, chromatin state, transcription factor.

BACKGROUND

The functional annotation of farm animal genomes is important for understanding traits with complex genetic architecture, such as disease resistance, growth, feed efficiency or reproduction [1,2,3,4]. Until recently, functional annotation mainly focused on protein-coding genes using transcriptomics. Transcriptome annotation is now consolidated with robust pipelines [5] and, throughout the years, transcriptome annotations for human [6], model species [7,8], terrestrial livestock [9,10,11] and some aquaculture species [12,13] have been published.

Non-coding regulatory elements, including promoters, enhancers, silencers and insulators, have been studied in several livestock species but are mostly unexplored in aquaculture species. These elements play essential roles in regulating gene expression and their state can change depending on tissue, cell type, sex, age, and health status [14]. Thus, annotation of regulatory elements in different contexts not only aids to address basic questions related to morphology and physiology [15,16], but also functional genomic responses to environmental variation [17,18,19]. Genetic variation at non-coding elements also underpins phenotypic variation [20,21] and can thus be leveraged to improve our ability to predict polygenic traits using genomic data [22]. In this respect, more than 90% of phenotype-associated SNPs identified in human GWAS are located in non-coding regions [23], with similar results reported for livestock [24].

In the past decade, human and livestock functional annotation initiatives have investigated epigenetic mechanisms involved in gene regulation through the study of chromatin state modifications across the genome [25]. Chromatin can switch dynamically between active and inactive states in minutes to hours, leaving epigenetic footprints that can be transmitted vertically following DNA replication [26,27]. Many different sequencing assays have been developed to infer chromatin epigenetic status, including chromatin accessibility (ATAC-Seq) [28], protein-DNA interactions (ChIP-Seq) [29] and long-range chromatin interactions (Hi-C)[30]. These and other assays are being applied by the FAANG Consortium [4,31,32,33]. Current annotations of chromatin state and regulatory elements remain limited to a few terrestrial farm animal species [9,16,34,35]. However, a catalogue of regulatory elements is being generated for several important fish species used as model species [36]and in global aquaculture [13], currently the fastest growing animal production sector [37,38].

Turbot (*Scophthalmus maximus*) is a valuable farmed fish in Europe and Asia (more than 100,000 tons), with the highest production in China [39] followed by Spain [40]. Turbot is in its 6th generation of selective breeding and infectious disease outbreaks constitute one of the main challenges this young industry faces [39,41]. This is a broader trend shared by global aquaculture, where infectious diseases cause losses of totalling more than 5,000 M€ per year [42]. Functional annotation of the turbot transcriptome has been performed against high-quality reference genomes [41,43,44,45], including for immune-organs stimulated with viruses [46], bacteria [47,48] and parasites [49,50,51,52]. Candidate genes for disease resistance have been further explored by mapping DEGs within QTL regions [53,54,55]. However, limited attention has been given to non-coding regulatory elements, beyond a recent analysis of chromatin accessibility focussed on early development [56]. How chromatin state and non-coding regulatory elements are regulated during immune responses remains undefined in turbot and scarcely explored in other farmed finfish.

The head kidney has been targeted in all previous functional genomics studies in turbot investigating pathogen responses [46,47,48,49,50,51,55], due to its central role in fish immunity [57]. Head kidney is a key lymphoid organ in most marine fishes and, analogous to the mammalian bone marrow, responsible for the production of multiple types of leukocytes, including B-lymphocytes, early-stage T-lymphocytes, as well myeloid cells such as granulocytes

and monocytes / macrophages [58,59,60]. Innate immunity provides the first line of defence against pathogens and acts following the binding of PAMPs to germline PRRs, leading to various effector cellular functions targeting pathogen destruction and clearance.

DATA DESCRIPTION

The aim of this study was to generate the first comprehensive functional annotation of the innate immune response of turbot using a chromosome-level reference genome sequence (ASM1334776v1) [41]. Live fish (18 individuals) and primary immune cell cultures (18 cultures) were stimulated using mimics of viral (poly I:C) and bacterial (killed *Vibrio anguillarum*) infections and compared to controls to capture changes in the transcriptome alongside chromatin accessibility and epigenetic state by integrating RNA-Seq, ATAC-Seq and ChIP-Seq data. The experimental design and assays followed the protocols established in the the European Commission Horizon 2020 AQUA-FAANG project (Grant Agreement 817923). We aimed to generate comparable datasets in response to the same bacterial and viral mimics in six commercially important farmed fish species: European seabass (*Dicentrarchus labrax*), gilthead seabream (*Sparus aurata*), rainbow trout (*Oncorhynchus mykiss*), Atlantic salmon (*Salmo salar*), common carp (*Cyprinus carpio*) and turbot (*Scophthalmus maximus*). Our results provide a deeper understanding of the epigenomic basis for innate immunity in turbot and a novel resource to prioritize genetic variation associated with non-coding elements regulating immune responses.

METHODS

Animals

Thirty 8-month-old immature turbot specimens provided by Stolt Sea Farm SA (Ribeira, Spain) were housed in indoor tanks with recirculating seawater at the facilities of the Aquarium of the University of Santiago de Compostela (Spain) for a period of acclimation of 15 d at 16 °C (**Supplementary table 1**). All fish were fasted for 24 h before stimulations were performed. Eighteen fish were stimulated *in vivo* by intraperitoneal injection, while the other 12 were used for leukocyte isolation for *in vitro* stimulation (see following sections). Fish were anesthetized by bath (MS-222; 100mg / L) and then euthanized by anaesthetic overdose (MS-222; 150 mg / L) before tissue sampling. All animal procedures were approved by the Bioethics Committee of the University of Santiago de Compostela (body authorized according to R.D. 53/2013) and with the authorization of the Xunta de Galicia Regional Government.

Protocols

Detailed protocols for the *in vivo* and *in vitro* stimulations, RNA isolation, ATAC-Seq and ChIP-Seq (including library preparation) followed for turbot are available in the FAANG repository (data.faang.org; URLs for protocols in **Supplementary tables 1 and 2**).

In vivo immunostimulation

Six fish were used per experimental condition for the *in vivo* stimulations: i) poly I:C for viral mimic immunostimulation; ii) killed *Vibrio anguillarum* for bacterial immunostimulation; and iii) phosphate buffered saline (PBS) for control. For poly I:C (Sigma P1530), we prepared a working stock at 5 mg / ml in PBS, preheated to 55 °C (15 min) and cooled at room temperature (20 min) before use. Fish were then injected intraperitoneally with 5 µg per g fish weight. For bacterial immunostimulation, an extract of *V. anguillarum* (strain P0382; INRA, France) was used. Bacteria were cultured in a tryptic soy broth medium to an OD600 (optical density at 600 nm) of 1.5. The bacterial pellet (derived from 100 ml of full-grown culture) was washed in an isotonic solution of NaCl (9 g / L) four times and resuspended in 1 ml of the same solution. Bacteria were killed by incubation for 30 sec at 100 °C, allowed to cool at room temperature and stored at -80 °C. The

bacteria extract was inoculated in each specimen, diluted in PBS (1:10) for a final volume of 100 µl. Control fish were injected with 100 µl of PBS. After 24 h, head kidney samples were extracted, washed with PBS and cut into at least three pieces (> 20 mg each); two were flash frozen on dry ice for ATAC-Seq and ChIP-Seq and the other was immersed in RNAlater (Thermofisher Scientific) for RNA extraction and RNA-Seq. All samples were then stored at -80°C (**Figure 7A**; **Supplementary table 2**).

In vitro stimulation: leukocyte isolation and culture

Leukocytes were isolated from 12 fish. The entire head kidney was aseptically isolated and placed in a Petri dish with 40 ml of cell isolation media (500 ml of Leibovitz L-15 medium (L-15), 10 ml FBS (2 %), 0.02 % EDTA). Samples were then cut into small pieces and passed through a 100 µm nylon mesh with constant flow of cell isolation media. Leukocytes were separated by centrifugation of 40 ml of the cell suspension gently layered in a 50 ml tube containing 51 % Percoll (400 x g, 30 min, 4 ºC, no brakes). The interface layer was collected by centrifugation (400 x g, 10 min, 4 ºC) and washed three times with L-15 medium containing 0.1 FBS, keeping the pelleted leukocytes (**Figure 7B**).

To ensure enough cells were available, samples were pooled by pairs after cell counting and cell viability was evaluated by the trypan blue exclusion test, totalling 6 pools (each 18 x 10^6 cells; **Supplementary table 1**). Then, each pool was divided into nine aliquots of 2 x 10^6 cells (in 2 ml) that were dispensed into wells, for a total of 54 wells (6 pools x 9 aliquots): 18 stimulated with 20 µl of poly I:C solution; 18 with 20 µl of inactivated *V. anguillarum*; and the remaining 18 wells used as controls (**Figure 7B**). All leukocyte cultures were incubated for 24 h at 16 °C. Cells were then collected in 2 ml Eppendorf tubes and pelleted at 500 x g for 5 mins at room temperature. Eighteen pellets were resuspended in RNAlater and stored at -80 °C for RNA-Seq, while the other 36 were flash frozen in dry ice and stored at -80 °C for ATAC-Seq and µChIPmentation, respectively (**Supplementary table 1**).

Genome reference

The genome (ASM1334776V1) used as reference was assembled at chromosome level and published by Martínez et al., 2021[41]. It consists of 145 contigs (contig N50: 20.4 Mb) and 127 scaffolds (scaffold N50: 22.9 Mb) capturing 22 chromosomes representing 98.6% of the genome.

RNA isolation and sequencing

After sample thawing, total RNA was extracted and purified using the miRNeasy Kit (QIAGEN) with specific modifications for: i) frozen head kidney (> 20 mg), following a protocol for “Total RNA extraction for tissues”, and ii) frozen leukocytes (2 x 10^6 cells) following a protocol for “Total RNA extraction for frozen cells” (**Supplementary table 2**). RNA integrity and quantity were evaluated in a Bioanalyzer (Bonsai Technologies, Madrid, Spain) and in a NanoDrop® ND-1000 spectrophotometer (NanoDrop® Technologies Inc., Wilmington, DE, USA). RNA integrity number (RIN) averaged 8.3 across all samples, always above 7.3. RNA samples were delivered to Novogene (UK) for library preparation using NEBNext Ultra Directional RNA Library Prep Kits for Illumina and sequenced using an Illumina NovaSeq S4 platform to generate 150 bp paired end reads.

RNA-Seq data processing

RNA-Seq data was processed using nf-core/rnaseq 3.10.1 [61] run with default parameters, using the turbot Ensembl genome ASM1334776v1 [41] as reference. In brief, the pipeline evaluated quality of raw reads using FASTQC (RRID:SCR_014583) [62] and trimmed adapters and low-quality bases using Trim Galore (RRID:SCR_011847) [63]. Reads were then mapped using STAR (RRID:SCR_004463) [64]. Normalized transcript read counts were obtained using RSEM

(RRID:SCR_000262) [65]. After nf-core processing, the resulting count tables were filtered to remove genes with expression below 5 transcripts per million (TPM < 5) and represented in only one sample across all conditions.

Differential gene expression and gene ontology analysis

Differentially expressed genes (DEGs) between stimulated and control samples were identified using the R/Bioconductor package DESeq2 v1.38.1 (RRID:SCR_015687) [67]. Genes with false discovery rate (FDR) adjusted $p < 0.05$ were considered DEGs. Functional enrichment of the DEG lists was performed using ShinyGO v0.77 (RRID:SCR_019213) [68]. Gene ontology (GO) terms for Biological Process of each list were ranked by statistical significance (FDR-adjusted $p < 0.05$). All expressed genes across conditions were used as the background for GO analyses.

ATAC-Seq: Library preparation and sequencing

Following a standard protocol for "Nuclei isolation for ATAC-Seq procedures" (**Supplementary table 2**), the frozen head kidney fragments (> 20 mg; *in vivo* assay) and cell pellets ($2x10^6$ cells; *in vitro* assay) were thawed and resuspended in 1 ml of TST buffer. Each tissue fragment was cut into smaller pieces with a scalpel, mashed with the rubber back of a syringe, and filtered through a 40 µm cell strainer, while cell pellets were resuspended by gentle pipetting. The number and integrity of nuclei was assessed with a haemocytometer (minimum of ~50,000 nuclei in a 16.5 µl suspension; ~3,000 nuclei / µl) before carrying out the Tn5 transposase reaction with Illumina Tagment DNA TDE1 enzyme (37°C, 30 min, 1000 RPM, Illumina) following the standard "OmniATAC protocol" (**Supplementary table 2**) [69]. The resulting DNA was purified with a MinElute PCR purification kit (Qiagen), and DNA concentration assessed with a Qubit using the dsDNA HS kit (ThermoFisher Scientific). Library amplification (10-12 PCR cycles) was carried out using the NEBNext Ultra II DNA Library Prep Kit (New England Biolabs), with IDT for Illumina UD Indexes (96x, Plate A, Set 1, Illumina). Library size selection was performed to remove fragments below 180 bp and above 700 bp using AMPure XP beads (Beckman coulter). Finally, DNA fragment size distribution was assessed with the Bioanalyzer High Sensitivity DNA Assay kit (Agilent Technologies). ATAC-Seq libraries were delivered to Novogene (UK) to be sequenced on an Illumina NovaSeq S4 platform generating 150 bp paired end reads.

ChIP-Seq and µChIPmentation: Library preparation and sequencing

The frozen head kidney fragments (> 20 mg) and leukocyte pellets ($2x10^6$ cells) were thawed on ice. Following a standard "ChIP-seq" protocol (**Supplementary table 2**), the tissue fragments were transferred into a Douncer homogenizer containing a protease inhibitor cocktail (PIC, Roche, 1 tablet in 50 ml of PBS) solution immersed in ice and homogenized using pestles A and B (from less to more plunger adjustment). The leukocyte pellets were resuspended in the PBS and PIC solution. The amount and quality of nuclei was assessed with a haemocytometer and trypan blue staining (> 10 million cells for head kidney; > 100,000 for leukocyte cultures). Due to the low number of nuclei recovered from the leukocyte cultures, a µChIPmentation protocol (Diagenode) was used, following a modified protocol (**Supplementary table 2**).

In both cases, chromatin crosslinking was done using a 1 % formaldehyde solution followed by quenching with glycine (0.125 M). Nuclei were pelleted and resuspended in complete sonication buffer, while leukocyte nuclei were resuspended in Hanks' Balanced Salt Solution (HBSS, Thermo Fisher Scientific) - tL1 buffer (**Supplementary table 2**). The chromatin was sheared using a Covaris S2 focused ultrasonicator with the following parameters: 2 % duty cycle, intensity 3, with 200 cycles per burst, at 4 °C for 8 min and 6 min for head kidney tissue and leukocytes, respectively.

The immunoprecipitation was performed using Diagenode antibodies for three marks: H3K4me3 (marking active promoter regions; cat. No. C15410003; 1.3 µg / µl), H3K27ac (marking active

enhancer and promoter regions; cat. No. C15410196; 2.8 µg / µl) and H3K27me3 (marking Polycomb repressed regions; cat. No. C15410195; 1.1 µg / µl). For head kidney samples, antibodies were coupled with pre-washed protein A and protein G beads, and the tubes left under rotation overnight (~16 h) at 4 °C, following the standard ChIP-Seq protocol. After washing the beads and decrosslinking, the samples were purified using the MinElute PCR purification kit (Qiagen) and later quantified with DNA HS Qubit (ThermoFisher Scientific). Immunoprecipitated chromatin was stored at -20 °C until library preparation, using a Microplex v3 kit (Diagenode). For the leukocyte samples, the µChipmentation kit for histones (Diagenode) was used for chromatin immunoprecipitation and ChIP-Seq library preparation (**Supplementary table 2**).

Before sequencing, the quantity and quality of purified libraries was assessed using the Qubit DNA HS kit (ThermoFisher Scientific) and the High Sensitivity DNA Assay kit (Agilent Technologies), respectively. A minimum of 60 % of the chromatin was required to have a size distribution between 200-700 bp (centered around 350-400 bp). ChIP-Seq libraries were delivered to Novogene (UK) for sequencing on an Illumina NovaSeq S4 platform generating 150 bp paired-end reads.

ATAC-Seq and ChIP-Seq data processing

ATAC-Seq and ChIP-Seq data were processed using the nf-core/atacseq v1.2.2. and nf-core/chipseq v1.2.2 pipelines [69], respectively, run with the narrow_peak option for ATAC-Seq and H3K4me3 and H3K27ac ChIP-Seq datasets, and with the broad_peak option for H3K27me3. The other parameters were kept by default. Quality assessment of the reads was carried out with FASTQC (RRID:SCR_014583) [62], and adapters and low-quality bases trimmed with Trim Galore (RRID:SCR_011847) [63]. Reads were mapped to the turbot genome using BWA (RRID:SCR_010910) [70]. Further filtering was done with SAMtools (RRID:SCR_002105) [71], BAMtools (RRID:SCR_015987) [72] and pysam (RRID:SCR_021017) [71]. Genome-wide immunoprecipitation (IP) enrichment relative to controls was done with deepTools (RRID:SCR_016366) [73] and broad / narrow peaks were called using MACS2 (RRID:SCR_013291) [74]. Once nf-core was finished, suboptimal replicates with very low peak numbers were excluded after visualizing their bigwig files on the Integrative Genomics Viewer (IGV; RRID:SCR_011793) [75] (**Supplementary table 3B**).

ChIP-Seq and µChIPmentation blacklist

To improve the signal-to-noise ratio of the ChIP-Seq and µChIPmentation data, a blacklist consisting of high signal and low mappability regions was constructed using ChIP-Seq and µChIPmentation inputs, including 21 control ChIPseq turbot samples (ENA accession PRJEB57784), following a publicly available pipeline [76]. The mappability of the turbot genome for read lengths of 100 bp and 150 bp (k-mers 100 and 150) was quantified using the umap software package (RRID:SCR_018217) [77]. The generated mappability files were fed into the ENCODE blacklist software [78] to generate the blacklist. Alignment and peak files derived from the nf-core pipeline were filtered with BAMtools to remove reads and peaks located in the blacklist regions.

Differential histone modification regions and differentially accessible regions

Significant DHMRs and DARs (adjusted $p < 0.05$) between stimulated samples and controls were identified using DiffBind (RRID:SCR_012918) [79] with default settings.

Integration of ChIP-Seq and ATAC-Seq data with RNA-Seq data

For each condition tested, the promoters of DEGs overlapping with regions tagged as DARs and / or DHMRs were identified. When applicable, a hypergeometric test was performed to check the significance of overlapping between each pair ($p < 0.05$, Bonferroni correction).

Chromatin state inferences

Genome-wide chromatin states for each condition were predicted using ChromHMM (RRID:SCR_018141) [80] integrating the ChIP-Seq data (µChIPmentation for *in vitro* samples) for the three histone marks (H3K4me3, H3K27ac and H3K27me3) and ATAC-Seq data. Chromatin state prediction was performed by testing ChromHMM models including from 8 to 15 states, keeping the one that returned the most biologically relevant chromatin states, for head kidney and leukocyte data separately [34,36,81]. The genome-wide distribution of resulting chromatin states was visualized on IGV (RRID:SCR_011793) [75]. Regions annotated as enhancer-related states by ChromHMM were retrieved, and each stimulation dataset was compared against its respective control to identify potential enhancer-related regions. Then, each list of regions including potential enhancers annotated as "intergenic" or "intron" were kept as differential enhancer-state regions for further analysis.

Transcription factor motif analysis

Enriched TFBM included in the HOMER software (RRID:SCR_010881) [82] were identified using the *findMotifsGenome.pl* function (settings: -size given -mask -mset vertebrates) in the different lists of promoter-associated DHMRs/DARs and putative enhancers for each condition. Random genomic regions with GC-content matching each input genomic list were used as the background for automatic motif analysis by HOMER. Following the recommended guidelines for the HOMER program, only TFBMs with $p < 0.05$ (Bonferroni correction) and percentage of target sequences > 10 % were considered enriched.

GO analysis of the TFs predicted to bind enriched TFBMs was performed with Metascape (RRID:SCR_016620) [83] using zebrafish *Danio rerio* as the reference. Finally, a selection of TF genes that were DEGs and contained promoter regions overlapping DARs or DHMRs were explored with IGV (RRID:SCR_011793) [75].

RESULTS

Raw sequencing data and sample metadata

A total of 186 multiomic datasets were produced in this study, including RNA-Seq (36), ATAC-Seq (36) and ChIP-Seq (108; 36 per histone mark, plus 6 ChIP-Seq input controls). Full information on samples and metadata is shared in **supplementary tables 1 and 2**.

RNA-Seq

On average, 69,580,246 raw reads per library were produced across the 36 RNA-Seq samples, with 97.2 % mapping to the turbot genome (**Supplementary table 3A**). Principal Component Analysis (PCA) showed that 77 % of the transcriptome variance was explained by PC1, separating the *in vivo* and *in vitro* stimulations (**Figure 2A**). Despite all three *in vivo* conditions (head kidney samples) grouping together, a suggestive spatial segregation was observed mainly across PC1, with the control at one end and the *Vibrio* stimulation at the other. For the *in vitro* stimulations (primary cell cultures of kidney-isolated leukocytes), PC2 clearly separated the *Vibrio* stimulation from poly I:C and control samples, the last two showing some overlap (**Figure 2B**).

Differential expression analysis was performed by comparing each stimulated condition to the respective controls, both for the *in vitro* and *in vivo* stimulations. In total, 8,797 DEGs were identified across all comparisons (**Table 1**, **Supplementary table 4**). For the *in vitro* stimulations, a stronger response was observed for *Vibrio* than poly I:C stimulation, both for up and downregulated genes. Meanwhile, poly I:C showed a higher number of DEGs than *Vibrio* for the *in vivo* stimulations both for up and downregulated genes (**Table 1, Supplementary table 4**). Overall, more DEGs were detected in the *in vitro* than in the *in vivo* stimulations (7,940 vs 5,758, respectively).

Functional enrichment among DEGs

GO analysis identified enriched biological processes for up and downregulated DEGs in all conditions (**Supplementary table 5**). Among the downregulated DEGs, metabolism, cell cycle and cytoskeleton organization terms were enriched for the *in vitro* stimulations, while a limited number of terms were detected for *in vivo* stimulations (**Supplementary table 5**).

More abundant and specific enriched GO terms were detected among the upregulated DEGs. Although RNA metabolism was enriched for most stimulations, poly I:C stimulation was linked to a more specific activation of key immune functions (particularly *in vitro*) such as interferon-stimulated genes and cytokine pathways, and regulation of toll-like receptors, besides more general immune terms. DEGs for the *Vibrio in vitro* stimulation were enriched for immune-related terms associated with cytokine and several transport pathways, whereas *Vibrio in vivo* upregulated genes displayed strong enrichment of terms related to cytoskeleton organization, tissue development and syncytium formation (**Supplementary table 5**).

Comparing upregulated DEGs between conditions, important immune-related GO terms were commonly enriched between both poly I:C and *Vibrio in vitro* stimulations, as well as the poly I:C *in vitro* and *in vivo* stimulations (**Figure 3; Supplementary table 6**; **Supplementary table 7**). These included interferon type I stimulated genes (*socs1a, socs1b, nod2, nmi*), cytokine signalling (the same genes plus *traf2* and *il15ra*) and MHC-I pathways (*erap1b*, *tapbpl*, *tapbp*.2). Activation of transcription, protein localization, and a small number of terms associated with "peptidyl-arginine modification" (*prmt1, prmt3, prmt5, prmt7*), critical to maintain self-antigenic integrity, were commonly overrepresented in poly I:C and *Vibrio in vivo*, as well as between the *in vitro* and *in vivo Vibrio* stimulations (**Figure 3**; **Supplementary table 6; Supplementary table 7**).

We detected several DEGs regulated in opposite directions for poly I:C and *Vibrio* stimulations (**Table 2; Figure 3; Supplementary table 6**), suggestive of divergent immune response to bacteria and virus. Among the *in vivo* upregulated DEGs for *Vibrio* and downregulated for poly I:C, we observed enrichment of general immune and inflammatory functions, including genes such as *il-1b*, *traf4a* and *f2r*. Conversely, immune-related genes including *nod2*, *sting1*, *irf1b* and *apaf1*, were downregulated for *Vibrio* and upregulated for poly I:C *in vivo*. Interestingly, many orthologs orthologs of human and mouse genes involved in the type-I IFN response [84,85] were upregulated in the poly I:C stimulations, while downregulated with *Vibrio* extracts, especially in the *in vivo* stimulation (**Table 2**).

Considering condition-specific DEGs (**Figure 3**; **Supplementary table 6**), those upregulated by poly I:C *in vitro* were enriched for terms associated with toll-like receptor signalling pathway (*usp4, tasl, irak3*), while transcriptional activation terms were found among upregulated DEGs for *Vibrio in vitro.* For the *in vivo* stimulations, transcriptional activation GO terms were enriched for upregulated DEGs after poly I:C stimulation, whereas response to stimulus (explained by *tlr3, hamp, tnip1, fxc1a* and *ccr12a*, among other genes) and syncytium formation (explained by *kirrel3l*, *jam2a*, *plekho1b*) were enriched for *Vibrio*.

ATAC-Seq and ChIP-Seq

Most libraries showed > 95% read mapping to the turbot genome, except four ATAC-Seq *Vibrio in vitro* libraries with lower mapping rates due to the presence of the bacterial DNA in the cell culture (average 54%) (**Supplementary table 3B**). On average, we identified 24,251 (*in vitro*) and 62,013 (*in vivo*) peaks per sample for ATAC-Seq; 26,199 (H3K4me3), 13,461 (H3K27ac) and 27,765 (H3K27me3) peaks for the *in vivo* ChIP-Seq data; and 15,870 (H3K4me3), 5,363 (H3K27ac) and 38,784 (H3K27me3) peaks for the *in vitro* ChIP-Seq data. Hierarchical clustering of the samples clustered them by technique and condition (**Figure 4**), excluding the H3K27ac mark, where *in vivo* and *in vitro* clusters for poli I:C were separated. This is concordant with the

behaviour observed for regulatory elements for this experimental condition, as discussed in future sections. In addition, the marks associated with repression (H3K27me3) and activation (H3K4me3) of regulatory elements showed, respectively, the expected negative and positive correlation with RNA-seq data. A similar pattern was observed in the PCA plot, where techniques were segregated mostly across PC1, unless for the repressive (H3K27me3), mostly explained by PC2, also responsible for differentiation across conditions (**Supplementary figure 1**). *Vibrio* showed the greatest differentiation among the stimulations, while poly I:C and controls were mostly intermingled, particularly for ATAC-Seq (*in vitro* and *in vivo*) and H3K4me3-ChIP-Seq (*in vitro*).

ChIP-Seq blacklist

Certain genomic regions obscure epigenetic analyses because of anomalous, unstructured, or high signal due to particular genomic features (Amemiya et al., 2019). Using the 21 ChIP-Seq and µChIPmentation input controls (ENA project PRJEB57784) we constructed a blacklist of low confidence genomic regions for turbot ChIP assays (**Supplementary table 8**). On average, 6.98 % (~39Mb) of the turbot genome was included in the blacklist, consisting of high input signal (5.58 %) and low mappability (1.40 %) regions (**Supplementary figure 2**).

Chromatin state annotation

Genome-wide chromatin state predictions were produced using ChromHMM employing the ChIP-Seq and ATAC-Seq data from stimulated and control samples from head kidney tissue (*in vivo*) and head kidney derived leukocytes (*in vitro*) (**Figure 4**, **Supplementary table 9**). For the *in vivo* samples (**Figure 5A**), a 10-state model was chosen including promoters / transcription start sites (TSS; States 1, 2, 3 and 4), potential enhancer regions (States 5 and 6), ATAC islands (State 7, i.e. ATAC-peaks lacking histone marks), repressed regions (States 8 and 9) and low signal regions (State 10). For the *in vitro* leukocytes (**Figure 5B**), an 8-state chromatin model was chosen including promoters / TSS (States 1, 2 and 3), potential enhancer regions (States 4 and 5), ATAC islands (State 6), repressed regions (State 7) and low signal regions (State 8). For each chromatin state map, ±2kb regions around the TSS showed signal specifically for chromatin states 1, 2, 3 and 4 for head kidney (*in vivo*) and states 1, 2 and 3 for leukocytes (*in vitro*), mostly corresponding to promoter regions and / or transcriptionally active regions.

Differential chromatin accessibility regions (DAR) and differential histone modification regions (DHMR) following stimulation

We next aimed to identify regulatory regions in the turbot genome affected by the immune stimulation. Significant DARs and DHMRs (FDR-adjusted $p < 0.05$) comparing *Vibrio* and poly I:C stimulations to control samples were identified (**Table 3, Supplementary table 10**). Globally, a higher number of DARs or DHMRs were detected for up- than for down-regulated regions, more *in vivo* than *in vitro*, and more for *Vibrio* than poly I:C comparisons. Additionally, histone marks

Association of ATAC-Seq and ChIP-Seq data with RNA-Seq data

We tested if DARs and DHMRs for H3K4me3 and H3K27ac marks annotated as promoter / TSS regions (up to -1kb upstream of TSSs) corresponded to DEGs under the same experimental conditions (Hypergeometric distribution test, $p < 0.05$). DARs and DHMRs were much more overrepresented at the promoter regions of up- rather than down-regulated DEGs (**Table 4, Supplementary table 11**), suggesting changes in chromatin state associated with the activation of genes. We performed GO enrichment analyses of those upregulated DEGs within each experimental condition t ($P < 0.05$, **Table 4**). Significant enrichment (FDR < 0.05) included several metabolic activities and particular immune functions, including antigen processing / presentation and apoptotic / cell death pathways (**Supplementary table 12**). Specifically, enriched terms were mostly associated with RNA processing (in particular tRNA, rRNA, ncRNA),

ribosome biogenesis, and translation for the *Vibrio in vitro* stimulation, whereas for protein localization to nucleus and organelle, DNA replication and carbohydrate metabolism for the *Vibrio in vivo* stimulation. The term 'peptidyl-arginine modification' was also enriched involving *prmt1, prmt3, prmt5* and *prmt7* genes, as outlined before for the whole DEG analysis (RNA-Seq). The poly I:C *in vivo* stimulation showed enrichment in immune-related processes, including 'antigen processing and presentation via MHC-I' (explained by *erap1b, tapbpl*) and 'regulation of programmed cell death' (explained by *apaf1, bida, casp8 and 10, socs3a, grinab, bcl2l10*; **Supplementary table 12**). No correlation was found between chromatin accessibility in promoter regions and differential expression among selected genes related with T-cell co-stimulation, pro- and anti-inflammatory cytokines and class IV TRIM genes typically involved in antiviral defence (**Supplementary figure 3**).

Transcription factor binding motif (TFBM) analysis

To establish TFs potentially associated with chromatin state regulation following immune stimulation, the enrichment of TFBMs within DAR / DHMRs annotated as promoter or enhancer regions were examined. Significant enrichment was detected for all stimulations. On average across stimulations, 56.5 (range: 0-109) significantly enriched TFBMs were detected for upregulated promoters, with only one significantly enriched TFBM was detected in downregulated promoters for *Vibrio in vivo.* Meanwhile 5 (range: 1-10) and 35.75 (range: 1-61) respective TFBMs were enriched for downregulated and upregulated enhancers (**Supplementary table 13**).

Most TFBMs and associated TFs predicted for active promoters and enhancers were enriched in multiple stimulation conditions: 46 TFs were shared by both *Vibrio* stimulations and *in vivo* poly I:C; 62 TFs were shared for both *Vibrio* stimulations; and 12 TFs were shared for both *in vivo* stimulations (**Figure 6**; **Supplementary table 13**).

To further investigate the functions of TFs with enriched motifs, we used the Molecular Complex Detection algorithm (MCODE) and protein-protein interaction data (PPI) to identify connected functions among differentially expressed TFs, and among TFs with DARs or DHMRs on their promoters, with a special focus on immune related functions (**Supplementary figures 4**, **Supplementary table 14**).

For the *Vibrio in vitro* stimulation, interconnected clusters of TFs associated with hemopoiesis and immune functions, particularly FGF signalling pathway, MAPK signalling pathway, lymphocyte activation, lymph vessel development and oxidative stress response were identified. For the *Vibrio in vivo* stimulation, we detected clusters of TFs associated with similar functions: hemopoiesis, MAPK signalling pathway and myeloid leukocyte differentiation and cellular senescence (**Supplementary figure 4**). Finally for the poly I:C *in vivo* stimulation, hemopoiesis, FGF signalling pathway, MAPK signalling pathway, myeloid cell development and differentiation, regulation of cell differentiation, showed connected TF subsets.

Using the Integrative Genomics Viewer (IGV), we visualized variation in chromatin state around the promoters of nine TF-DEGs selected from **Supplementary table 14**, as well as the toll-like receptor *tlr3* gene, to show differences following activation by the different stimulants (**Figure 7**; **Supplementary figures 5 and 6**).

DISCUSSION

This study represents the first epigenomic analysis of the turbot head kidney, the primary hematopoietic and lymphoid organ of teleosts, in response to viral (poly I:C) and bacterial (inactivated *V. anguillarum*) mimics. While head kidney transcriptomic responses to bacteria,

virus and parasites have been extensively investigated in turbot [46,48,50,51,52,86,87,88], the epigenetic regulation of chromatin states following immune stimulation has not been explored before. The head kidney response was explored through intraperitoneal injection, reflecting the response in the whole-body, including interactions among immune organs, and through *in vitro* stimulation of primary leukocyte cultures, reflecting their direct interaction with stimulants. The use of the same viral and bacterial mimics will enable further comparative evaluation among the six fish species included in the AQUA-FAANG project pertaining to five teleost orders (Salmoniformes, Cyprinidontiformes, Spariformes, Perciformes and Pleuronectiformes). We first discuss the transcriptional response to immune stimulations, which provide context to the chromatin assays performed to understand how transcription is regulated from transcription factors up to immune genes in the turbot.

Transcriptomic response to immune stimulation

Upregulated genes with key immune roles, such as interferon and cytokine pathways and toll-like receptor regulation, were found in both poly I:C stimulations and in *Vibrio in vitro* stimulation. Interferons (IFNs) are a subset of class II cytokines with crucial roles in antimicrobial defence especially against viruses, but also intracellular bacteria [89]. Interestingly, *tlr3*, encoding the toll-like receptor 3, which induces IFN production after interaction with poly I:C [90], was upregulated in the *Vibrio in vivo* stimulation but not differentially expressed in poly I:C *in vivo* and downregulated after both *in vitro* stimulations. We also checked the chromatin state distribution along the *tlr3* gene (**Supplementary figure 6**) in both *in vitro* and *in vivo* conditions. Overall, no significant changes were observed. Most of the chromatin states found within the promoter and the gene body were annotated as strongly active promoter / transcript, flanking active TSS without ATAC and ATAC islands (shared by both chromatin state models). Additionally, we observed a weak active promoter / transcript state annotated only in the *in vivo* condition. Overall, the lack of chromatin regulation suggests that promoter accessibility is not alone sufficient for *tlr3* expression. The asynchronism between chromatin accessibility of regulatory elements and gene expression has been reported previously, with promoter peaks preceding gene expression, and distal regulatory elements such as enhancers appearing accessible when gene expression starts [91,92,93]. In fact, we observed an active enhancer signal (both weak and strong states) close to the distal promoter region of *tlr3* in the *Vibrio in vivo* stimulation only, which may be linked to the expression of this or other genes in that condition.

Regardless, signal exhaustion 24-hour post-injection (hpi) might explain *tlr3* downregulation *in vitro*, this would not be expected for the *in vivo* Poly I:C stimulation where the peak of the *in vivo* Poly I:C response for this gene has been reported to occur later in turbot (3 dpi) [94]. Long term activation of *tlr3* by Poly I:C has been reported to cause long term physiological impairment in different mouse tissues [95,96]. The tissue expression profile of *tlr3* can however vary between tissues and cell types in different species; although similar expression was observed between zebrafish and rainbow trout tissues [97], this gene's expression was limited to liver and digestive organs in *Fugu* [98], while in human vs. mouse, *tlr3* was expressed in different myeloid cell types [99].

We cannot discard alternative hypotheses related to changes or diversification of the TLR3 signalling pathway in flatfish or specifically in turbot, as suggested here by *tlr3* activation in response to *Vibrio in vivo* stimulation and to the parasite *P. dicentrarchi* by Figueras et al. (2016) [43]. In fact, we identified other interferon-stimulated DEGs in poly I:C both *in vitro* and *in vivo*, including *socs1a* and b (suppressor of cytokine signalling 1), *nod2* (nucleotide binding oligomerization domain containing 2) and *nmi* (N-Myc interactor), typically participating in type I IFN responses [100,101]. *socs1* is a conserved, inducible negative regulator of IFN [100], which has been described before in other finfish species as being responsive to viral [102,103] and bacterial stimulation [104]. This gene is involved in tissue homeostasis following the IFN

response, marking a critical checkpoint in immune homeostasis as an expected player to be found in extended immune responses [105].

On the other hand, *nod2* which encodes for a highly conserved intracellular receptor triggering innate antibacterial and antiviral signalling pathways in fish, including IFN [106,107,108,109], and *nmi* , another highly conserved ISG which increases STAT-mediated transcription in response to IFN-gamma in mammals and fish [110,111,112,113], were also activated after poly I:C stimulation. The activation of conserved genes involved in positive and negative regulation of IFN- signalling suggests a fine adjustment to an intense IFN response to recover tissue homeostasis [114,115]. Also, the upregulation of these four key genes after *Vibrio in vitro* stimulation is consistent with past reports that place IFN regulation as a key point of the immune response [116,117]. In this regard, comparative analysis in the AQUAFAANG project will enlighten conserved and specific mechanisms of immune response regulation across different farmed fish lineages. Future studies in turbot should explore shorter periods following *in vitro* stimulation to provide further understanding of the head kidney response to viral and bacterial mimics, as done in other species [118,119].

Upregulated DEGs shared by both *in vivo* stimulations were involved in the activation of transcription and translation and protein modification / localization. This included four members of the conserved immune-related protein arginine methyltransferase family (*prmt1*, *prmt3*, *prmt5* and *prmt7*), enriched under the term "peptidyl-arginine modification". *prmt* family members regulate transcription and translation and are also involved in signal transduction during inflammation and responses against poly I:C and bacterial lipopolysaccharide (LPS), both in mammals and finfish species [120,121,122,123]. These four genes were also activated in both *Vibrio* stimulations, suggesting an important role in the response to *Vibrio*.

We also verified if immune genes could be regulated in opposite directions after stimulation with viral and bacterial mimics, which could reflect antagonistic immune responses. Increased susceptibility to bacterial superinfections induced by innate antiviral responses has been reported in several models in mammals [124] in type I and type II IFN responses [125,126]. Such mechanisms are likely present in teleosts due to the high conservation of these pathways, and in fact, different patterns of resistance to viral and bacterial infections were observed among isogenic rainbow trout lines [127]. However, no negative correlations for resistance to bacteria and viruses has been observed in the few studies addressing this issue in relation to selective breeding [128,129,130]. Indeed, understanding the cellular basis of antagonistic responses to viruses and bacteria in fish will require more research, but here we identified examples of opposite gene expression responses, including genes from the core type I IFN response, conserved between teleosts and humans [85,131], such as *sting1* (stimulator of interferon response CGAMP interactor 1) [93,132]*, irf1b* (interferon regulatory factor 1) [133,134] and *nod2* (mentioned above), downregulated by *Vibrio* and upregulated by poly I:C *in vivo*. Reciprocally, the critical pro-inflammatory cytokine *il1b* (interleukin 1 beta) [135,136] and the regulator of inflammation *traf4a* (TNF receptor associated factor 4) [137,138] were upregulated by *Vibrio* but downregulated by poly I: C. Interestingly, a majority of genes induced by poly I:C and repressed by *Vibrio* extracts had human / mouse ISG orthologs (22 / 42 *in vitro*, 18 / 34 *in vivo*); in contrast, many genes induced by *Vibrio* extracts and repressed by poly I:C, especially *in vivo,* had human / mouse orthologs downregulated by type I IFN. Overall, these observations indicate that a significant part of these contrasted responses is mediated by genes functionally conserved between fish and mammals.

Epigenetic assays and their association with transcriptomic response

Activation and binding of transcription factors (TFs) to regulatory regions lies at the top of specific transcriptome cascade responses [139,140]. In our study, the exploration of promoters of differentially expressed TFs, also involving differential accessibility regions (DARs) and differential

histone modification regions (DHMRs), barely showed changes in the chromatin state distribution between treatments (**Supplementary Figure 5**). This was the case for *egr1* (early growth response 1), which plays a key role in cell survival, macrophage proliferation and cell death in teleost and mammals [141,142] and regulates the expression of *il1b* and *cxcl2* (CXC motif chemokine ligand 2). The same was observed for *meis1* (myeloid ecotropic viral integration site homeobox 1), related to hematopoiesis in mammals and teleost [143,144]. In all these examples, the TF genes showed bivalent / poised promoters in the three *in vivo* samples (control, *Vibrio* and poly I:C). A similar situation was found *in vitro*, where promoters of genes, such as *mitf* (melanocyte inducing transcription factor), showed activation signals regardless of the experimental condition. In previous studies, these bivalent / poised states have been interpreted as a mechanism allowing rapid responses, e.g. for genes induced during the pro-inflammatory response [119,145] or during mammal embryonic development and in germ cells [146,147]. Thus, the expression of primary response genes, such as the TF genes exemplified above, may be allowed by permissive chromatin states.

The presence of differential activation signals at promoter regions, either in the form of open chromatin regions or the histone marks H3K4me3 and H3K27ac, are associated with differential gene expression [148]. H3K27ac is widely accepted as one of the most dynamic activation marks in eukaryotes [149]. Interestingly, we did not find important changes between experimental conditions in our study, where H3K4me3 was the most dynamic among the ChIP-Seq marks. We suspect that the increase in H3K4me3 could also be an effect of sampling being conducted 24 hours post inoculation (hpi), especially for the *in vivo* stimulations. Previous studies have reported that H3K4me3 may not have an active role in activating transcription, an effect that should be detectable early after stimulation, but instead in marking transcriptional activity itself, suggesting that H3K4me3 modification may have a role in maintaining transcriptional consistency or memory of previous states [150].

DEG promoters overlapped with DAR / DHMRs for upregulated genes in most conditions (**Table 4**). GO enrichment of DEGs with activation signals at promoters (**Supplementary table 12**) was similar to that observed with the whole DEG dataset (**Supplementary table 5**). *Vibrio* stimulations were particularly enriched in terms associated with transcription activation, but also with some immune functions, while poly I:C (especially *in vivo*) showed a more specific response, enriched in immune-related processes (**Supplementary table 6**). However, DEGs associated with some key immune functions in the transcriptomic analysis, such as response to cytokine stimulus, did not show DAR / DHMRs, suggesting that these gene promoters could be already accessible before stimulation. Cytokines are one of the core initiators of the inflammatory response, thus a bivalent state, ready for activation or inactivation of expression, might explain this observation (**Figure 6**).

Regardless of predicted chromatin states around gene promoters, we observed an increased expression of immune-related genes following poly I:C and Vibrio stimulation suggesting further chromatin unpacking (**Table 3**), even if chromatin state is not strongly affected. In fact, many differential regions were detected between stimulations, particularly DARs (i.e. ATAC-Seq) and DHMRs (for H3K4me3) for *Vibrio*. In comparison, fewer DARs and DHMRs were detected for the poly I:C stimulations. This could be interpreted as a return, especially *in vitro*, to the native chromatin state after 24 hpi, which may suggest the initial response at this point is exhausted, as suggested by the transcriptomic data.

The intersection between DEGs and DARs was specifically inspected for a selection of immune genes related to T-cell activation (as a checkpoint for the transition to cellular adaptative immunity), pro and anti-inflammatory cytokines (related with early and late immune response, respectively) and class IV TRIM genes (associated mainly with antiviral responses; **Supplementary figure 3**). Again, no clear correlation was found between differential promoter

accessibility and differential gene expression in any of the conditions. In fact, most promoters for the targeted genes were not even differentially accessible, adding to the suggestion that poised states are a common feature among immune-related genes. Both *cd28* (co-activator) and *ctla4* (co-inhibitor), conserved mediators in vertebrate T-cell activation [151,152], did not show significant changes in chromatin accessibility between comparisons, except for *Vibrio in vitro*, where the promoter was differentially accessible. The same was true at the level of gene expression, though, as both *Vibrio* assays showed significant downregulation of the *cd28* gene. Looking at anti-inflammatory cytokines, the conserved IL10 and a subunit of IL35 (encoded by *ebi3*) were significantly upregulated in *Vibrio in vitro* and Poly I:C *in vivo* in the case of *il10*, and in both Poly I:C assays for *il35* (*ebi3*). For pro-inflammatory cytokines, different sets of genes were regulated for *Vibrio in vitro* (*il17a/f1, il17a/f2*, upregulated), Poly I:C *in vivo* (*il1b*, downregulated) and *Vibrio in vivo* (*il1b*, upregulated). All conditions showed upregulation of both anti- and pro-inflammatory cytokines, except Poly I:C *in vivo*, which showed upregulation of all these genes. The mixture of anti- and pro-inflammation signals suggests an advanced response to the stimulants, while illustrating the complexity of their coordinated contributions to the regulation of inflammatory response. Finally, class IV TRIM genes, which participate in innate immunity [153] mostly showed significant upregulation in Poly I:C stimulations, consistent with their conserved role in antiviral responses [154]. Interestingly, little significant differential expression was found in the *Vibrio* stimulations (none *in vivo*).

The signals detected for the three histone marks and open chromatin regions were integrated to define chromatin states in *in vivo* and *in vitro* assays. Two chromatin state models differing on the number of states for the *in vitro* (8) and the *in vivo* (10) assays were annotated based on the emission parameters of each epigenetic mark. Due to the high conservation of the assayed histone marks among eukaryotes [155,156], we also compared our chromatin state models with other studies using the same histone marks [31,119,157,158]. The rationale for running two models arose from the differences in biological material and different methodologies applied for the ChIP-Seq assays (classic ChIP-Seq in the *in vivo* assays vs µChIPmentation in *in vitro*). In fact, the 9- and 10-state iterations of the *in vitro* assay resulted in 1 and 2 redundant chromatin states, respectively, reducing the quality of the models.

Indeed, even a 7-state model (Strongly active promoter / transcript, flanking active TSS without ATAC, bivalent / poised TSS, strong active enhancer, ATAC island, repressed polycomb and low signal states) showed nearly similar emission parameters and distribution in the TSS neighbourhood, suggesting a general conservation of chromatin states between the whole head kidney and the kidney leukocyte fraction. Only one *in vitro* exclusive chromatin state (bivalent / poised enhancer) and three *in vivo* exclusive chromatin states (weak active promoter / transcript, weak enhancer and weak repressed polycomb) were inferred from the two independent models. Although the absence of these three weak states in the *in vitro* assay can be explained due to the limited resolution of an 8-state model compared to a 10-state one, we hypothesize that the presence of a bivalent enhancer state in the *in vitro* but not the *in vivo* assay can be the result of the enrichment of immune cells and lack of contribution of non-leukocytic cells missing in the *in vitro* assay, where bivalent / poised states have been suggested to be relevant in the fast regulation of immune pathways [145,159,160]. Altogether, the integration of all histone marks in chromatin states allowed us to identify the co-occurrence of different histone marks, uncovering different combinations of activator signals (H3K4me3 + H3K27ac) and with repressor signals (H3K27me3).

TFBMs enriched within DARs / DHMRs in the promoters and enhancer-state regions predicted by ChromHMM of upregulated DEGs, provided a more detailed picture of regulatory elements changed by the immune stimulations (**Figure 6**, **Supplementary table 13**). Most of the enriched TFBMs were shared between at least two treatments, which supports their general regulatory function. Among those shared between most stimulations (excluding poly I:C *in vitro*), we found

several TFBMs of the ETS family, which are involved in cell proliferation, apoptosis and lymphocyte development [161]. We also found TFBM enrichment for PU.1, a master TF for the myeloid lineage, that promotes chromatin accessibility, also identified in a similar study in pig [119,162,163]. PU.1 is thought to promote binding of other TFs enriched in our study, including AP1. This protein is activated by TLRs during the immune response [164] and regulates gene expression in response to cytokines, stress, and bacterial or viral infections [165], and notably the IFN regulatory factors (IRFs, particularly IRF3, IRF4 and IRF8). The IRFs are key regulators of innate antiviral and antibacterial responses in vertebrates including fish [166,167]. The observed ontology enrichment clustering of these TFs reinforces the similarities between the conditions in our study (**Supplementary figure 4**).

To illustrate the functionality of our turbot epigenomic atlas, we explored the chromatin state changes in a selection of nine TF genes (**Figure 7, Supplementary figure 5**). These genes were differentially expressed and showed DARs / DHMRs in their promoters. Immune-responsive chromatin state remodelling was found, for instance, for *irf8* (interferon regulatory factor 8) in the *Vibrio in vitro* stimulation. Here, an extension of the “strongly active promoter” state around the TSS region was visible compared to the non-stimulated and poly I:C stimulated samples, alongside an overall expansion of the “strongly active transcript” and “ATAC island” states downstream. IRF8 is a key regulator of the NF-κB signalling pathway during inflammation, along with IRF3 [168]. Both IRF genes were differentially expressed in poly I:C and *Vibrio in vitro* stimulations (*irf8* also in poly I:C *in vivo*) and showed promoter DARs in *Vibrio* stimulations.

Another example was found in *Vibrio* and poly I:C *in vivo* stimulations for *bcl11a* (BCL11 transcription factor A), a key regulator of dendritic cell differentiation [169] and negative regulator of p53 [170]. This gene was differentially expressed in both stimulations but only showed a DAR promoter in response to *Vibrio* stimulation. The promoter / TSS region was annotated by ChromHMM as “weak repressed Polycomb” in the control, which changed in both the *Vibrio* and poly I:C stimulations, extending to “bivalent / poised” state, and in the case of *Vibrio* showing an extension to “Strongly active promoter / transcript” state. Weak and strong signals of active enhancers were also detected in the poly I:C and *Vibrio* stimulations within the second intron of the *bcl11a*, that were not present in the controls (**Supplementary figure 5**). A similar situation was found *in vitro* for *foxo1a* (Forkhead box O1), a TF-coding gene that participates in mucosal (innate) immune response regulating the expression of antimicrobial peptides and promoting phagocytosis during bacterial and parasitic infections [171,172]. This gene was differentially expressed in *Vibrio in vitro* and showed an extension of the “Strongly active promoter” state compared to the control and specially the poly I:C stimulation, while conserving the “bivalent / poised TSS” stretches downstream of the promoter. This was followed by many “ATAC islands” along the gene that were also present, although more scarcely, in the control, and almost absent in poly I:C. Finally, a “medium enhancer” state was detected within the first intron, present only in the *Vibrio* condition. These observations highlight the relevance of epigenetic chromatin marks at the first intron and other intronic regions associated with gene expression modulation [173,174], as target regulatory annotations to explore functional variants for disease resistance and selective breeding.

CONCLUSIONS

In summary, our study provides the first atlas of regulatory elements in turbot head kidney and leukocytes during the early response to viral and bacterial stimulation, as a contribution to the AQUA-FAANG project within the umbrella of the FAANG initiative. The integration of ATAC-Seq and ChIP-Seq data suggests that changes in chromatin state distribution were not as frequent between stimulations and controls as expected. However, the presence of DARs and DHMRs between the stimulations and the controls, which broadly overlapped with DEGs, provides clear

evidence for gene regulation at the epigenetic level that underpins changes in gene expression driving immune functions.

POTENTIAL IMPLICATIONS

Overall, this epigenomic atlas will help to decode the molecular mechanisms underlying turbot immune responses to viral and bacterial stimuli and offers a novel resource for developing selective breeding strategies for controlling diseases, one of the main concerns of turbot industry. Future work will benefit from linking the regulatory annotations generated in this study with genetic variants defined by whole genome re-sequencing [13], to help prioritize causal genetic variants for disease resistance traits underpinned by gene expression responses to pathogens.

LIST OF ABBREVIATIONS

ATAC – assay for transposase-accesible chromatin; ChIP – chromatin immunoprecipitation; DARs – differentially accessible regions; DEGs – differentially expressed genes; DHMRs – differential histone modification regions; FAANG – functional annotation of animal genomes; FDR – false discovery rate; FGF – fibrinogen growth factor; GWAS – genome wide association studies; GO – gene ontology; IFN – interferon; ISG – interferon stimulated genes; IGV – interactive genome browser; MHC – molecular histocompatibility complex; PAMPs – pathogen associated molecular patterns; PC – principal component; Poly I:C - polyinosinic:polycytidylic acid; PRRs – pattern recognition receptors; SNPs – single nucleotide polymorphisms; TF – transcription factor; TFBM – transcription factor binding motif; TLR – toll-like receptor; TSS -transcription starting site

DATA ACCESS

All raw RNA-Seq, ATAC-Seq and ChIP-Seq datasets can be accessed through the ENA repository under accession numbers PRJEB47933, PRJEB47934 and PRJEB57784, respectively. Detailed metadata for the samples and prepared libraries are available in **Supplementary tables 1 and 2**, respectively. Detailed experimental protocols are publicly available in the FAANG repository (data.faang.org) and following the URLs facilitated in **Supplementary tables 1 and 2**.

ETHICAL STATEMENT

All animal procedures were approved by the Bioethics Committee of the University of Santiago de Compostela (body authorized according to R.D. 53/2013) and with the authorization of the Xunta de Galicia Regional Government. All animal procedures were carried out in the Animalary of the Faculty of Biology of the University of Santiago de Compostela (Registry ID: ES150780263301) under the supervision of Prof. Jesús Lamas Fernández.

COMPETING INTEREST STATEMENT

The authors declare no competing interests.

ACKNOWLEDGMENTS

We acknowledge the technical support and informatic resources provided by the Centro de Supercomputación de Galicia (CESGA). We acknowledge Professor Carolina Tafalla (INIA-CSIC) for the support given in the revision of this article.

AUTHOR CONTRIBUTIONS

OA: Methodology, Software, Formal analysis, Investigation, Data Curation, Visualization, Writing – original draft, Writing – review & editing; **BGP:** Methodology, Investigation, Resources, Writing – review & editing; **PRV:** Investigation, Resources, Writing – review & editing; **ABH:** Software, Formal Analysis, Data Curation, Visualization, Writing – review & editing; **JL**: Investigation,

Resources, Writing – review & editing; **PD:** Software, Formal Analysis, Writing – review & editing; **DPM:** Software, Formal Analysis, Writing – review & editing; **PB**: Resources, Writing – review & editing; **DM**: Funding Acquisition, Methodology, Supervision, Resources, Writing – review & editing; **CB**: Conceptualization, Formal Analysis, Resources, Writing – review & editing, Project Administration, Supervision; **PM**: Conceptualization, Methodology, Formal Analysis, Resources, Writing – review & editing, Project Administration, Supervision.

FUNDING

This study was funded by the AQUA-FAANG project, which received funding from the European Union's Horizon 2020 research and innovation programme under grant agreement No 817923. Additional funding was provided by Xunta de Galicia local government (Spain) (ED431C 2022/33), which also supported the research fellowships of OA and PRV (refs. ED481A-2020/119 and ED481A-2020/491430, respectively). Contributions from the Roslin Institute were further supported by the BBSRC Institutional Strategic Programme grants BBS/E/D/10002070, BBS/E/D/20002174, BBS/E/RL/230001B and BBS/E/RL/230002B.

REFERENCES

1. Andersson L, Archibald AL, Bottema CD, Brauning R, Burgess SC, Burt DW, Casas E, Cheng HH, Clarke L, Couldrey C et al. 2015. Coordinated international action to accelerate genome-to-phenome with FAANG, the Functional Annotation of Animal Genomes project. *Genome Biology*, **16**(1), 57. doi: 10.1186/s13059-015-0622-4.
2. Tuggle CK, Giuffra E, White SN, Clarke L, Zhou H, Ross PJ, Acloque H, Reecy JM, Archibald A, Bellone RR et al. 2016. GO-FAANG meeting: a Gathering On Functional Annotation of Animal Genomes. *Animal Genetics*, **47**(5), 528–533. doi: 10.1111/age.12466.
3. Giuffra E, Tuggle CK. 2019. Functional Annotation of Animal Genomes (FAANG): Current Achievements and Roadmap. *Annual Review of Animal Biosciences*, **7**(1), 65–88. doi: 10.1146/annurev-animal-020518-114913.
4. Clark EL, Archibald AL, Daetwyler HD, Groenen MAM, Harrison PW, Houston RD, Kühn C, Lien S, Macqueen DJ, Reecy JM et al. 2020. From FAANG to fork: application of highly annotated genomes to improve farmed animal production. *Genome Biology*, **21**(1), 285. doi: 10.1186/s13059-020-02197-8.
5. Raghavan V, Kraft L, Mesny F, Rigerte L. 2022. A simple guide to *de novo* transcriptome assembly and annotation. *Briefings in Bioinformatics*, **23**(2). doi: 10.1093/bib/bbab563.
6. Frankish A, Carbonell-Sala S, Diekhans M, Jungreis I, Loveland JE, Mudge JM, Sisu C, Wright JC, Arnan C, Barnes I et al. 2023. GENCODE: reference annotation for the human and mouse genomes in 2023. *Nucleic Acids Research*, **51**(D1), D942–D949. doi: 10.1093/nar/gkac1071
7. He P, Williams BA, Trout D, Marinov GK, Amrhein H, Berghella L, Goh ST, Plajzer-Frick I, Afzal V, Pennacchio LA et al. 2020. The changing mouse embryo transcriptome at whole tissue and single-cell resolution. *Nature*, **583**(7818), 760–767. doi: 10.1038/s41586-020-2536-x.
8. Lawson ND, Li R, Shin M, Grosse A, Yukselen O, Stone OA, Kucukural A, Zhu L. 2020. An improved zebrafish transcriptome annotation for sensitive and comprehensive detection of cell type-specific genes. *ELife*, **9**. doi: 10.7554/eLife.55792.
9. Summers KM, Bush SJ, Wu C, Su AI, Muriuki C, Clark EL, Finlayson HA, Eory L, Waddell LA, Talbot R et al. 2020. Functional Annotation of the Transcriptome of the Pig, *Sus scrofa*, Based Upon Network Analysis of an RNAseq Transcriptional Atlas. *Frontiers in Genetics*, **10**. doi: 10.3389/fgene.2019.01355.
10. Halstead MM, Islas-Trejo A, Goszczynski DE, Medrano JF, Zhou H, Ross PJ. 2021. Large-Scale Multiplexing Permits Full-Length Transcriptome Annotation of 32 Bovine Tissues

from a Single Nanopore Flow Cell. *Frontiers in Genetics*, **12**. doi: 10.3389/fgene.2021.664260.

11. Overbey EG, Ng TT, Catini P, Griggs LM, StewartP, Tkalcic S, Hawkins RD, Drechsler Y. 2021. Transcriptomes of an Array of Chbiicken Ovary, Intestinal, and Immune Cells and Tissues. *Frontiers in Genetics*, **12**. doi: 10.3389/fgene.2021.664424.
12. Ramberg S, Høyheim B, Østbye TKK, Andreassen R. 2021. A *de novo* Full-Length mRNA Transcriptome Generated from Hybrid-Corrected PacBio Long-Reads Improves the Transcript Annotation and Identifies Thousands of Novel Splice Variants in Atlantic Salmon. *Frontiers in Genetics*, **12**. doi: 10.3389/fgene.2021.656334.
13. Johnston IA, Kent MP, Boudinot P, Looseley M, Bargelloni L, Faggion S, Merino GA, Ilsley GR, Bobe J, Tsigenopoulos CS et al. 2024. Advancing fish breeding in aquaculture through genome functional annotation. *Aquaculture*, **583**, 740589. doi: 10.1016/j.aquaculture.2024.740589
14. Kellis M, Wold B, Snyder MP, Bernstein BE, Kundaje A, Marinov GK, Ward LD, Birney E, Crawford GE, Dekker J et al. 2014. Defining functional DNA elements in the human genome. *Proceedings of the National Academy of Sciences*, **111**(17), 6131–6138. doi: 10.1073/pnas.1318948111.
15. Halstead MM, Ma X, Zhou C, Schultz RM, Ross PJ. 2020. Chromatin remodelling in bovine embryos indicates species-specific regulation of genome activation. *Nature Communications*, **11**(1), 4654. doi: 10.1038/s41467-020-18508-3.
16. Pan Z, Wang Y, Wang M, Wang Y, Zhu X, Gu S, Zhong C, An L, Shan M, Damas J et al. 2023. An atlas of regulatory elements in chicken: A resource for chicken genetics and genomics. *Science Advances*, **9**(18). doi: 10.1126/sciadv.ade1204.
17. Feinberg AP. 2007. Phenotypic plasticity and the epigenetics of human disease. *Nature*, **447**(7143), 433–440. doi: 10.1038/nature05919.
18. Ecker S, Pancaldi V, Valencia A, Beck S, Paul DS. 2018. Epigenetic and Transcriptional Variability Shape Phenotypic Plasticity. *BioEssays*, **40**(2), 1700148. doi: 10.1002/bies.201700148.
19. Hu J, Barrett RDH. 2017. Epigenetics in natural animal populations. *Journal of Evolutionary Biology*, **30**(9), 1612–1632. doi: 10.1111/jeb.13130.
20. Villar D, Frost S, Deloukas P, Tinker A. 2020. The contribution of non-coding regulatory elements to cardiovascular disease. *Open Biology*, **10**(7). doi: 10.1098/rsob.200088.
21. Boltsis I, Grosveld F, Giraud G, Kolovos P. 2021. Chromatin Conformation in Development and Disease. *Frontiers in Cell and Developmental Biology*, **9**. doi: 10.3389/fcell.2021.723859.
22. Zhu XN, Wang YZ, Li C, Wu HY, Zhang R, Hu XX. 2023. Chicken chromatin accessibility atlas accelerates epigenetic annotation of birds and gene fine-mapping associated with growth traits. *Zoological Research*, **44**(1), 53–62. doi: 10.24272/j.issn.2095-8137.2022.228.
23. Giral H, Landmesser U, Kratzer A. 2018. Into the Wild: GWAS Exploration of Non-coding RNAs. *Frontiers in Cardiovascular Medicine*, **5**. doi: 10.3389/fcvm.2018.00181.
24. Prowse-Wilkins CP, Wang J, Xiang R, Garner JB, Goddard ME, Chamberlain AJ. 2021. Putative Causal Variants Are Enriched in Annotated Functional Regions from Six Bovine Tissues. *Frontiers in Genetics*, **12**. doi: 10.3389/fgene.2021.664379.
25. Yan F, Powell DR, Curtis DJ, Wong NC. 2020a. From reads to insight: a hitchhiker's guide to ATAC-seq data analysis. *Genome Biology*, **21**(1), 22. doi: 10.1186/s13059-020-1929-3.
26. Moazed D. 2011. Mechanisms for the Inheritance of Chromatin States. *Cell*, **146**(4), 510–518. doi: 10.1016/j.cell.2011.07.013.
27. Cuvier O, Fierz B. 2017. Dynamic chromatin technologies: from individual molecules to epigenomic regulation in cells. *Nature Reviews Genetics*, **18**(8), 457–472. doi: 10.1038/nrg.2017.28.

28. Buenrostro JD, Wu B, Chang HY, Greenleaf WJ. 2015. ATAC-seq: A Method for Assaying Chromatin Accessibility Genome-Wide. *Current Protocols in Molecular Biology*, **109**(1). doi: 10.1002/0471142727.mb2129s109.
29. Park PJ. 2009. ChIP–seq: advantages and challenges of a maturing technology. *Nature Reviews Genetics*, **10**(10), 669–680. doi: 10.1038/nrg2641.
30. van Berkum NL, Lieberman-Aiden E, Williams L, Imakaev M, Gnirke A, Mirny LA, Dekker J, Lander ES. 2010. Hi-C: A Method to Study the Three-dimensional Architecture of Genomes. *Journal of Visualized Experiments*, **39**. doi: 10.3791/1869.
31. Fang L, Liu S, Liu M, Kang X, Lin S, Li B, Connor EE, Baldwin RL, Tenesa A, Ma L et al. 2019. Functional annotation of the cattle genome through systematic discovery and characterization of chromatin states and butyrate-induced variations. *BMC Biology*, **17**(1), 68. doi: 10.1186/s12915-019-0687-8.
32. Foissac S, Djebali S, Munyard K, Vialaneix N, Rau A, Muret K, Esquerré D, Zytnicki M, Derrien T, Bardou P et al. 2019. Multi-species annotation of transcriptome and chromatin structure in domesticated animals. *BMC Biology*, **17**(1), 108. doi: 10.1186/s12915-019-0726-5.
33. Liu S, Yu Y, Zhang S, Cole JB, Tenesa A, Wang T, McDaneld TG, Ma L, Liu GE, Fang L. 2020. Epigenomics and genotype-phenotype association analyses reveal conserved genetic architecture of complex traits in cattle and human. *BMC Biology*, **18**(1), 80. doi: 10.1186/s12915-020-00792-6.
34. Pan Z, Yao Y, Yin H, Cai Z, Wang Y, Bai L, Kern C, Halstead M, Chanthavixay G, Trakooljul N et al. 2021. Pig genome functional annotation enhances the biological interpretation of complex traits and human disease. *Nature Communications*, **12**(1), 5848. doi: 10.1038/s41467-021-26153-7.
35. Xiang R, MacLeod IM, Daetwyler HD, de Jong G, O'Connor E, Schrooten C, Chamberlain AJ, Goddard ME. 2021. Genome-wide fine-mapping identifies pleiotropic and functional variants that predict many traits across global cattle populations. *Nature Communications*, **12**(1), 860. doi: 10.1038/s41467-021-21001-0.
36. Baranasic D, Hörtenhuber M, Balwierz PJ, Zehnder T, Mukarram AK, Nepal C, Várnai C, Hadzhiev Y, Jimenez-Gonzalez A, Li N et al. 2022. Multiomic atlas with functional stratification and developmental dynamics of zebrafish cis-regulatory elements. *Nat Genet* **54**, 1037–1050 (2022). doi: 10.1038/s41588-022-01089-w.
37. Subasinghe R, Soto D, Jia J. 2009. Global aquaculture and its role in sustainable development. *Reviews in Aquaculture*, **1**(1), 2–9. doi: 10.1111/j.1753-5131.2008.01002.x.
38. Troell M, Costa-Pierce B, Stead S, Cottrell RS, Brugere C, Farmery AK, Little DC, Strand Å, Pullin R, Soto D et al. 2023. Perspectives on aquaculture's contribution to the Sustainable Development Goals for improved human and planetary health. *Journal of the World Aquaculture Society*, **54**(2), 251–342. doi: 10.1111/jwas.12946.
39. Gao Y, Wang Q, Liu Y, Ma Y, Jin H, Liu J, Wang H, Yan Y, Li J. 2023. Epidemiology of turbot bacterial diseases in China between October 2016 and December 2019. *Frontiers in Marine Science*, **10**. doi: 10.3389/fmars.2023.1145083.
40. APROMAR. 2022. La acuicultura en España 2022. *Asociación Empresarial de Acuicultura de España, Cádiz* (Online, accessed October 17, 2023 at www.apromar.es)
41. Martínez P, Robledo D, Taboada X, Blanco A, Moser M, Maroso F, Hermida M, Gómez-Tato A, Álvarez-Blázquez B, Cabaleiro S et al. 2021. A genome-wide association study, supported by a new chromosome-level genome assembly, suggests sox2 as a main driver of the undifferentiatiated ZZ/ZW sex determination of turbot (*Scophthalmus maximus*). *Genomics*, **113**(4), 1705–1718. doi: 10.1016/j.ygeno.2021.04.007.
42. Mishra S, Das R, Swain P. 2018. Status of Fish Diseases in Aquaculture and Assessment of Economic Loss Due to Disease; Today and Tomorrow's Printers and Publishers: New Delhi, India, Volume 1.

43. Figueras A, Robledo D, Corvelo A, Hermida M, Pereiro P, Rubiolo JA, Gómez-Garrido J, Carreté L, Bello X, Gut M et al. 2016. Whole genome sequencing of turbot (*Scophthalmus maximus*; *Pleuronectiformes*): a fish adapted to demersal life. *DNA Research*, **23**(3), 181–192. doi: 10.1093/dnares/dsw007.
44. Maroso F, Hermida M, Millán A, Blanco A, Saura M, Fernández A, Dalla-Rovere G, Bargelloni L, Cabaleiro S, Villanueva B et al. 2018. Highly dense linkage maps from 31 full-sibling families of turbot (*Scophthalmus maximus*) provide insights into recombination patterns and chromosome rearrangements throughout a newly refined genome assembly. *DNA Research*, **25**(4), 439–450. doi: 10.1093/dnares/dsy015.
45. Xu X, Shao C, Xu H, Zhou Q, You F, Wang N, Li W, Li M, Chen S. 2020. Draft genomes of female and male turbot *Scophthalmus maximus*. *Scientific Data*, **7**(1), 90. doi: 10.1038/s41597-020-0426-6.
46. Díaz-Rosales P, Romero A, Balseiro P, Dios S, Novoa B, Figueras A. 2012. Microarray-Based Identification of Differentially Expressed Genes in Families of Turbot (*Scophthalmus maximus*) After Infection with Viral Haemorrhagic Septicaemia Virus (VHSV). *Marine Biotechnology*, **14**(5), 515–529. doi:10.1007/s10126-012-9465-0.
47. Millán A, Gómez-Tato A, Pardo BG, Fernández C, Bouza C, Vera M, Alvarez-Dios JA, Cabaleiro S, Lamas J, Lemos ML et al. 2011. Gene Expression Profiles of the Spleen, Liver, and Head Kidney in Turbot (*Scophthalmus maximus*) Along the Infection Process with *Aeromonas salmonicida* Using an Immune-Enriched Oligo-microarray. *Marine Biotechnology*, **13**(6), 1099–1114. doi: 10.1007/s10126-011-9374-7.
48. Librán-Pérez M, Pereiro P, Figueras A, Novoa B. 2022. Transcriptome Analysis of Turbot (*Scophthalmus maximus*) Infected with *Aeromonas salmonicida* Reveals a Direct Effect on Leptin Synthesis as a Neuroendocrine Mediator of Inflammation and Metabolism Regulation. *Frontiers in Marine Science*, **9**. doi: 10.3389/fmars.2022.888115.
49. Pardo BG, Millán A, Gómez-Tato A, Fernández C, Bouza C, Alvarez-Dios JA, Cabaleiro S, Lamas J, Leiro JM, Martínez P. 2012. Gene Expression Profiles of Spleen, Liver, and Head Kidney in Turbot (*Scophthalmus maximus*) Along the Infection Process with *Philasterides dicentrarchi* Using an Immune-Enriched Oligo-Microarray. *Marine Biotechnology*, **14**(5), 570–582. doi: 10.1007/s10126-012-9440-9.
50. Robledo D, Ronza P, Harrison PW, Losada AP, Bermúdez R, Pardo BG, José-Redondo M, Sitjà-Bobadilla A, Quiroga MI, Martínez P. 2014. RNA-seq analysis reveals significant transcriptome changes in turbot (*Scophthalmus maximus*) suffering severe enteromyxosis. *BMC Genomics*, **15**(1), 1149. doi: 10.1186/1471-2164-15-1149.
51. Ronza P, Robledo D, Bermúdez R, Losada AP, Pardo BG, Sitjà-Bobadilla A, Quiroga MI, Martínez P. 2016. RNA-seq analysis of early enteromyxosis in turbot (*Scophthalmus maximus*): new insights into parasite invasion and immune evasion strategies. *International Journal for Parasitology*, **46**(8), 507–517. doi: 10.1016/j.ijpara.2016.03.007.
52. Valle A, Leiro JM, Pereiro P, Figueras A, Novoa B, Dirks RPH, Lamas J. 2020. Interactions between the Parasite Philasterides dicentrarchi and the Immune System of the Turbot *Scophthalmus maximus*. A Transcriptomic Analysis. *Biology*, **9**(10), 337. doi: 10.3390/biology9100337.
53. Martínez P. 2016. Genomics advances for boosting aquaculture breeding programs in Spain. *Aquaculture*, **464**, 117–120. doi: 10.1016/j.aquaculture.2016.06.021.
54. Saura M, Carabaño MJ, Fernández A, Cabaleiro S, Doeschl-Wilson AB, Anacleto O, Maroso F, Millán A, Hermida M, Fernández C et al. 2019. Disentangling Genetic Variation for Resistance and Endurance to Scuticociliatosis in Turbot Using Pedigree and Genomic Information. *Frontiers in Genetics*, **10**. doi: 10.3389/fgene.2019.00539.
55. Aramburu O, Blanco A, Bouza C, Martínez P. 2023. Integration of host-pathogen functional genomics data into the chromosome-level genome assembly of turbot

(*Scophthalmus maximus*). *Aquaculture*, **564**, 739067. doi: 10.1016/j.aquaculture.2022.739067.

56. Guerrero-Peña L, Suarez-Bregua P, Gil-Gálvez A, Naranjo S, Méndez-Martínez L, Tur R, García-Fernández P, Tena JJ, Rotllant J. 2023. Genome-wide chromatin accessibility and gene expression profiling during flatfish metamorphosis. *Scientific Data*, **10**(1), 196. doi: 10.1038/s41597-023-02111-4.
57. Mokhtar DM, Zaccone G, Alesci A, Kuciel M, Hussein MT, Sayed RKA. 2023. Main Components of Fish Immunity: An Overview of the Fish Immune System. *Fishes*, **8**(2), 93. doi: 10.3390/fishes8020093.
58. Klosterhoff MC, Pereira-Junio J, Rodrigues RV, Gusmão EP, Sampaio LA, Tesser MB, Romano LA. 2015. Ontogenic development of kidney, thymus and spleen and phenotypic expression of CD3 and CD4 receptors on the lymphocytes of cobia (*Rachycentroncanadum*). *Anais Da Academia Brasileira de Ciências*, **87**(4), 2111–2121. doi: 10.1590/0001-3765201520140623.
59. Geven EJW, Klaren PHM. 2017. The teleost head kidney: Integrating thyroid and immune signalling. *Developmental & Comparative Immunology*, 66, 73–83. doi: 10.1016/j.dci.2016.06.025.
60. Chen W, Huang J, Wang W, Wang Y, Chen H, Wang Q, Zhang Y, Liu Q, Yang D. 2022. Multi-tissue scRNA-seq reveals immune cell landscape of turbot (*Scophthalmus maximus*). *Fundamental Research*, **2**(4), 550–561. doi: 10.1016/j.fmre.2021.12.015.
61. Patel H, Ewels P, Peltzer A, Botvinnik O, Sturm G, Moreno D, Vemuri P, Garcia MU, Morins S, Pantano L et al. 2023. nf-core/rnaseq: nf-core/rnaseq v3.10.1 – Plastered Rhodium Rudolph. *Zenodo*. doi: 10.5281/zenodo.7505987.
62. Andrews S. 2010. FastQC: A Quality Control Tool for High Throughput Sequence Data. Available online at: http://www.bioinformatics.babraham.ac.uk/projects/fastqc/
63. Martin M. 2011. Cutadapt removes adapter sequences from high-throughput sequencing reads. *EMBnet.Journal*, **17**(1), 10. doi: 10.14806/ej.17.1.200.
64. Dobin A, Davis CA, Schlesinger F, Drenkow J, Zaleski C, Jha S, Batut P, Chaisson M, Gingeras TR. 2013. STAR: ultrafast universal RNA-seq aligner. *Bioinformatics*, **29**(1), 15–21. doi: 10.1093/bioinformatics/bts635.
65. Li B, Dewey CN. 2011. RSEM: accurate transcript quantification from RNA-Seq data with or without a reference genome. *BMC Bioinformatics*, **12**(1), 323. doi: 10.1186/1471-2105-12-323.
66. Love MI, Huber W, Anders S. 2014. Moderated estimation of fold change and dispersion for RNA-seq data with DESeq2. *Genome Biology*, **15**(12), 550. doi: 10.1186/s13059-014-0550-8.
67. Ge SX, Jung D, Yao R. 2020. ShinyGO: a graphical gene-set enrichment tool for animals and plants. *Bioinformatics*, **36**(8), 2628–2629. doi: 10.1093/bioinformatics/btz931.
68. Corces MR, Trevino AE, Hamilton EG, Greenside PG, Sinnott-Armstrong NA, Vesuna S, Satpathy AT, Rubin AJ, Montine KS, Wu B et al. 2017. An improved ATAC-seq protocol reduces background and enables interrogation of frozen tissues. *Nature Methods*, **14**(10), 959–962. doi: 10.1038/nmeth.4396.
69. Ewels P, Peltzer A, Fillinger S, Patel H, Alneberg J, Wilm A, Ulysse-Garcia M, Di Tommaso P, Nahnsen S. 2023. The nf-core framework for community-curated bioinformatics pipelines. *Zenodo*. doi: 10.5281/zenodo.3240506.
70. Li H, Durbin R. 2009. Fast and accurate short read alignment with Burrows–Wheeler transform. *Bioinformatics*, **25**(14), 1754–1760. doi: 10.1093/bioinformatics/btp324.
71. Danecek P, Bonfield JK, Liddle J, Marshall J, Ohan V, Pollard MO, Whitwham A, Keane T, McCarthy SA, Davies RM et al. 2021. Twelve years of SAMtools and BCFtools. *GigaScience*, **10**(2). doi: 10.1093/gigascience/giab008.

72. Barnett DW, Garrison EK, Quinlan AR, Strömberg MP, Marth GT. 2011. BamTools: a C++ API and toolkit for analyzing and managing BAM files. *Bioinformatics*, **27**(12), 1691–1692. doi: 10.1093/bioinformatics/btr174.
73. Ramírez F, Dündar F, Diehl S, Grüning BA, Manke T. 2014. deepTools: a flexible platform for exploring deep-sequencing data. *Nucleic Acids Research*, **42**(W1), W187–W191. doi: 10.1093/nar/gku365.
74. Zhang Y, Liu T, Meyer CA, Eeckhoute J, Johnson DS, Bernstein BE, Nusbaum C, Myers RM, Brown M, Li W et al. 2008. Model-based Analysis of ChIP-Seq (MACS). *Genome Biology*, **9**(9), R137. doi: 10.1186/gb-2008-9-9-r137.
75. Robinson JT, Thorvaldsdottir H, Turner D, Mesirov JP. 2023. igv.js: an embeddable JavaScript implementation of the Integrative Genomics Viewer (IGV). *Bioinformatics*, **39**(1). doi: 10.1093/bioinformatics/btac830.
76. Dewari, P. 2023. Create blacklist file for ChIP-seq analysis. GitHub. https://github.com/Pooran-Dewari/create-blacklist-fie-for-ChIP-seq-analysis.
77. Karimzadeh M, Ernst C, Kundaje A, Hoffman MM. 2018. Umap and Bismap: quantifying genome and methylome mappability. *Nucleic Acids Research*. doi: 10.1093/nar/gky677.
78. Amemiya HM, Kundaje A, Boyle AP. 2019. The ENCODE Blacklist: Identification of Problematic Regions of the Genome. *Sci Rep* **9**, 9354. doi: 10.1038/s41598-019-45839-z.
79. Stark R, Brown G. 2011. DiffBind: differential binding analysis of ChIP-Seq peak data. *Bioconductor*. doi: 10.18129/B9.bioc.DiffBind.
80. Ernst J, Kellis M. 2012. ChromHMM: automating chromatin-state discovery and characterization. *Nature Methods*, **9**(3), 215–216. doi: 10.1038/nmeth.1906.
81. Vu H, Ernst J. 2022. Universal annotation of the human genome through integration of over a thousand epigenomic datasets. *Genome Biology*, **23**(1), 9. doi: 10.1186/s13059-021-02572-z.
82. Heinz S, Benner C, Spann N, Bertolino E, Lin YC, Laslo P, Cheng JX, Murre C, Singh H, Glass CK. 2010. Simple Combinations of Lineage-Determining Transcription Factors Prime cis-Regulatory Elements Required for Macrophage and B Cell Identities. *Molecular Cell*, **38**(4), 576–589. doi: 10.1016/j.molcel.2010.05.004.
83. Zhou Y, Zhou B, Pache L, Chang M, Khodabakhshi AH, Tanaseichuk O, Benner C, Chanda SK. 2019. Metascape provides a biologist-oriented resource for the analysis of systems-level datasets. *Nature Communications*, **10**(1), 1523. doi: 10.1038/s41467-019-09234-6.
84. Rusinova I, Forster S, Yu S, Kannan A, Masse M, Cumming H, Chapman R, Hertzog PJ. 2012. INTERFEROME v2.0: an updated database of annotated interferon-regulated genes. *Nucleic Acids Research*, **41**(D1), D1040–D1046. doi: 10.1093/nar/gks1215.
85. Clark TC, Naseer S, Gundappa MK, Laurent A, Perquis A, Collet B, Macqueen DJ, Martin SAM, Boudinot P. 2023. Conserved and divergent arms of the antiviral response in the duplicated genomes of salmonid fishes. *Genomics*, **115**(4), 110663. doi: 10.1016/j.ygeno.2023.110663.
86. Pardo BG, Fernández C, Millán A, Bouza C, Vázquez-López A, Vera M, Alvarez-Dios JA, Calaza M, Gómez-Tato A, Vázquez M et al. 2008. Expressed sequence tags (ESTs) from immune tissues of turbot (*Scophthalmus maximus*) challenged with pathogens. *BMC Veterinary Research*, **4**(1), 37. doi: 10.1186/1746-6148-4-37.
87. Millán A, Gómez-Tato A, Fernández C, Pardo BG, Álvarez-Dios JA, Calaza M, Bouza C, Vázquez M, Cabaleiro S, Martínez P. 2010. Design and Performance of a Turbot (*Scophthalmus maximus*) Oligo-microarray Based on ESTs from Immune Tissues. *Marine Biotechnology*, **12**(4), 452–465. doi: 10.1007/s10126-009-9231-0.
88. Domínguez B, Pardo BG, Noia M, Millán A, Gómez-Tato A, Martínez P, Leiro J, Lamas J. 2013. Microarray analysis of the inflammatory and immune responses in head kidney turbot leucocytes treated with resveratrol. *International Immunopharmacology*, **15**(3), 588–596. doi: 10.1016/j.intimp.2013.01.024.

89. Gan Z, Chen SN, Huang B, Zou J, Nie P. 2020. Fish type I and type II interferons: composition, receptor usage, production and function. *Reviews in Aquaculture*, **12**(2), 773–804. doi: 10.1111/raq.12349.
90. Kumar A, Zhang J, Yu FSX. 2006. Toll-like receptor 3 agonist poly(I:C)-induced antiviral response in human corneal epithelial cells. *Immunology*, **117**(1), 11–21. doi: 10.1111/j.1365-2567.2005.02258.x.
91. Ma S, Zhang B, LaFave LM, Earl AS, Chiang Z, Hu Y, Ding J, Brack A, Kartha VK, Tay T, Law T, Lareau C, Hsu YC, Regev A, Buenrostro JD. 2020. Chromatin Potential Identified by Shared Single-Cell Profiling of RNA and Chromatin. *Cell*, 183(4), 1103-1116.e20. doi: 10.1016/j.cell.2020.09.056.
92. Wike CL, Guo Y, Tan M, Nakamura R, Shaw DK, Díaz N, Whittaker-Tademy AF, Durand NC, Aiden EL, Vaquerizas JM, Grunwald D, Takeda H, Cairns BR. 2021. Chromatin architecture transitions from zebrafish sperm through early embryogenesis. *Genome Research*, 31(6), 981–994. doi: 10.1101/gr.269860.120.
93. Liu R, Meng F, Li X, Li H, Yang G, Shan S. 2023. Characterization of STING from common carp (*Cyprinus carpio L.)* involved in spring viremia of carp virus infection. *Fish & Shellfish Immunology*, *142*, 109164. doi: 10.1016/j.fsi.2023.109164.
94. Pereiro P, Figueras A, Novoa B. 2025. Exploring common modulations induced by three fish RNA viruses in turbot (*Scophthalmus maximus*): Cholesterol, vitamin D3 and retinol metabolism as shared targets. *Aquaculture*, 595, 741525. doi: 10.1016/j.aquaculture.2024.741525.
95. Stowell NC, Seideman J, Raymond HA, Smalley KA, Lamb RJ, Egenolf DD, Bugelski PJ, Murray LA, Marsters PA, Bunting RA, Flavell RA, Alexopoulou L, San Mateo LR, Griswold DE, Sarisky RT, Mbow ML, Das AM (2009). Long-term activation of TLR3 by Poly(I:C) induces inflammation and impairs lung function in mice. *Respiratory Research*, *10*(1), 43. doi: 10.1186/1465-9921-10-43.
96. Gao T, Zhang S, Wang J, Liu L, Wang Y, Cao Z, Hu Q, Yuan W, Lin L. 2018. TLR3 contributes to persistent autophagy and heart failure in mice after myocardial infarction. *Journal of Cellular and Molecular Medicine*, *22*(1), 395–408. doi: 10.1111/jcmm.13328.
97. Rodriguez MF, Wiens GD, Purcell MK, Palti Y. 2005. Characterization of Toll-like receptor 3 gene in rainbow trout (*Oncorhynchus mykiss*). *Immunogenetics*, *57*(7), 510–519. doi: 10.1007/s00251-005-0013-1.
98. Oshiumi H, Tsujita T, Shida K, Matsumoto M, Ikeo K, Seya T. 2003. Prediction of the prototype of the human Toll-like receptor gene family from the pufferfish, Fugu rubripes, genome. *Immunogenetics*, *54*(11), 791–800. doi: 10.1007/s00251-002-0519-8.
99. Muzio M, Bosisio D, Polentarutti N, D'amico G, Stoppacciaro A, Mancinelli R, van't Veer C, Penton-Rol G, Ruco LP, Allavena P, Mantovani A. 2000. Differential Expression and Regulation of Toll-Like Receptors (TLR) in Human Leukocytes: Selective Expression of TLR3 in Dendritic Cells. *The Journal of Immunology*, *164*(11), 5998–6004. doi: 10.4049/jimmunol.164.11.5998.
100. Blumer T, Coto-Llerena M, Duong FHT, Heim MH. 2017. SOCS1 is an inducible negative regulator of interferon λ (IFN-λ)–induced gene expression in vivo. *Journal of Biological Chemistry*, **292**(43), 17928–17938. doi: 10.1074/jbc.M117.788877.
101. Ahn JH, Park JY, Kim DY, Lee TS, Jung DH, Kim YJ, Lee YJ, Lee YJ, Seo IS, Song EJ et al. 2021. Type I Interferons Are Involved in the Intracellular Growth Control of *Mycobacterium abscessus* by Mediating NOD2-Induced Production of Nitric Oxide in Macrophages. Frontiers in Immunology, **12**. doi: 10.3389/fimmu.2021.738070.
102. Jin HJ, Xiang LX, Shao JZ. 2007. Identification and characterization of suppressor of cytokine signaling 1 (SOCS-1) homologues in teleost fish. *Immunogenetics*, *59*(8), 673–686. doi: 10.1007/s00251-007-0232-8.
103. Sobhkhez M, Joensen LL, Tollersrud LG, Strandskog G, Thim HL, Jørgensen JB. 2017. A conserved inhibitory role of suppressor of cytokine signaling 1 (SOCS1) in salmon

antiviral immunity. *Developmental & Comparative Immunology*, *67*, 66–76. doi: 10.1016/j.dci.2016.11.001

104. Wang G, Liu W, Wang C, Wang J, Liu H, Hao D, Zhang M. 2022. Molecular characterization and immunoregulatory analysis of suppressors of cytokine signaling 1 (SOCS1) in black rockfish, *Sebastes schlegeli*. *Developmental & Comparative Immunology*, *130*, 104355. doi: 10.1016/j.dci.2022.104355.

105. Bidgood GM, Keating N, Doggett K, Nicholson SE. 2024. SOCS1 is a critical checkpoint in immune homeostasis, inflammation and tumor immunity. *Frontiers in Immunology*, *15*. doi: 10.3389/fimmu.2024.1419951.

106. Zou PF, Chang MX, Li Y, Xue NN, Li JH, Chen SN, Nie P. 2016. NOD2 in zebrafish functions in antibacterial and also antiviral responses via NF-κB, and also MDA5, RIG-I and MAVS. *Fish & Shellfish Immunology*, *55*, 173–185. doi: 10.1016/j.fsi.2016.05.031

107. Howe K, Schiffer PH, Zielinski J, Wiehe T, Laird GK, Marioni JC, Soylemez O, Kondrashov F, Leptin M. 2016. Structure and evolutionary history of a large family of NLR proteins in the zebrafish. *Open Biology*, *6*(4), 160009. doi: 10.1098/rsob.160009.

108. Nie L, Xu XX, Xiang LX, Shao JZ, Chen J. 2017. Mutual Regulation of NOD2 and RIG-I in Zebrafish Provides Insights into the Coordination between Innate Antibacterial and Antiviral Signaling Pathways. *International Journal of Molecular Sciences*, **18**(6), 1147. doi: 10.3390/ijms18061147.

109. Mojzesz M, Rakus K, Chadzinska M, Nakagami K, Biswas G, Sakai M Hikima J. 2020. Cytosolic Sensors for Pathogenic Viral and Bacterial Nucleic Acids in Fish. *International Journal of Molecular Sciences*, *21*(19), 7289. doi: 10.3390/ijms21197289.

110. Zhu M, John S, Berg M, Leonard WJ. 1999. Functional Association of Nmi with Stat5 and Stat1 in IL-2- and IFN γ-Mediated Signaling. *Cell*, **96**(1), 121–130. doi: 10.1016/S0092-8674(00)80965-4.

111. Levraud JP, Jouneau L, Briolat V, Laghi V, Boudinot P. 2019. IFN-Stimulated Genes in Zebrafish and Humans Define an Ancient Arsenal of Antiviral Immunity. *The Journal of Immunology*, **203**(12), 3361–3373. doi: 10.4049/jimmunol.1900804.

112. Li L, Chen SN, Li N, Nie P. 2022. Molecular characterization and transcriptional conservation of N-myc-interactor, Nmi, by type I and type II IFNs in mandarin fish Siniperca chuatsi. *Developmental & Comparative Immunology*, *130*, 104354. doi: 10.1016/j.dci.2022.104354.

113. Li L, Chen SN, Wang KL, Li N, Pang AN, Liu LH, Li B, Hou J, Wang S, Nie P. 2023. Interaction of Nmi and IFP35 Promotes Mutual Protein Stabilization and IRF3 and IRF7 Degradation to Suppress Type I IFN Production in Teleost Fish. *The Journal of Immunology*, *210*(10), 1494–1507. doi: 10.4049/jimmunol.2300012.

114. Ivashkiv LB, Donlin LT. 2014. Regulation of type I interferon responses. *Nature Reviews Immunology*, **14**(1), 36–49. doi:10.1038/nri3581.

115. Murira A, Lamarre A. 2016. Type-I Interferon Responses: From Friend to Foe in the Battle against Chronic Viral Infection. *Frontiers in Immunology*, **7.** doi: 10.3389/fimmu.2016.00609.

116. Kovarik P, Castiglia V, Ivin M, Ebner F (2016). Type I Interferons in Bacterial Infections: A Balancing Act. *Frontiers in Immunology*, *7*. doi: 10.3389/fimmu.2016.00652.

117. Kopitar-Jerala N. 2017. The Role of Interferons in Inflammation and Inflammasome Activation. *Frontiers in Immunology*, **8**. doi: 10.3389/fimmu.2017.00873.

118. Hasanuzzaman AFM, Cao A, Ronza P, Fernández-Boo S, Rubiolo JA, Robledo D, Gómez-Tato A, Alvarez-Dios JA, Pardo BG, Villalba A et al. 2020. New insights into the Manila clam – *Perkinsus olseni* interaction based on gene expression analysis of clam hemocytes and parasite trophozoites through in vitro challenges. *International Journal for Parasitology*, **50**(3), 195–208. doi: 10.1016/j.ijpara.2019.11.008.

119. Herrera-Uribe J, Liu H, Byrne KA, Bond ZF, Loving CL, Tuggle CK. 2020. Changes in H3K27ac at Gene Regulatory Regions in Porcine Alveolar Macrophages Following LPS or PolyIC Exposure. Frontiers in Genetics, **11**. doi: 10.3389/fgene.2020.00817.
120. Hung CM, Li C. 2004. Identification and phylogenetic analyses of the protein arginine methyltransferase gene family in fish and ascidians. *Gene*, *340*(2), 179–187. doi: 10.1016/j.gene.2004.07.039.
121. Dong CW, Zhang YB, Lu AJ, Zhu R, Zhang FT, Zhang QY, Gui JF. 2007. Molecular characterisation and inductive expression of a fish protein arginine methyltransferase 1 gene in response to virus infection. *Fish & Shellfish Immunology*, *22*(4), 380–393. doi: 10.1016/j.fsi.2006.06.010.
122. Wang Y, Li C. 2012. Evolutionarily conserved protein arginine methyltransferases in non-mammalian animal systems. *The FEBS Journal*, *279*(6), 932–945. doi: 10.1111/j.1742-4658.2012.08490.x.
123. Srour N, Khan S, Richard S. 2022. The Influence of Arginine Methylation in Immunity and Inflammation. *Journal of Inflammation Research*, *Volume* **15**, 2939–2958. doi: 10.2147/JIR.S364190.
124. Barman TK, Metzger DW. 2021. Disease Tolerance during Viral-Bacterial Co-Infections. *Viruses*, **13**(12), 2362. doi: 10.3390/v13122362.
125. Navarini AA, Recher M, Lang KS, Georgiev P, Meury S, Bergthaler A, Flatz L, Bille J, Landmann R, Odermatt B et al. 2006. Increased susceptibility to bacterial superinfection as a consequence of innate antiviral responses. *Proceedings of the National Academy of Sciences*, **103**(42), 15535–15539. doi: 10.1073/pnas.0607325103.
126. Sun K, Metzger DW. 2008. Inhibition of pulmonary antibacterial defense by interferon-γ during recovery from influenza infection. *Nature Medicine*, **14**(5), 558–564. doi: 10.1038/nm1765.
127. Fraslin C, Quillet E, Rochat T, Dechamp N, Bernardet JF, Collet B, Lallias D, Boudinot P. 2020. Combining Multiple Approaches and Models to Dissect the Genetic Architecture of Resistance to Infections in Fish. *Frontiers in Genetics*, **11**. doi: 10.3389/fgene.2020.00677.
128. Ødegård J, Olesen I, Gjerde B, Klemetsdal G. 2007. Positive genetic correlation between resistance to bacterial (furunculosis) and viral (infectious salmon anaemia) diseases in farmed Atlantic salmon (*Salmo salar*). *Aquaculture,* ***271***(1–4), 173–177. doi: 10.1016/j.aquaculture.2007.06.006.
129. Ødegård J, Baranski M, Gjerde B, Gjedrem T. 2011. Methodology for genetic evaluation of disease resistance in aquaculture species: challenges and future prospects. *Aquaculture Research*, **42**, 103–114. doi: 10.1111/j.1365-2109.2010.02669.x.
130. Bangera R, Ødegård J, Præbel AK, Mortensen A, Nielsen HM. 2011. Genetic correlations between growth rate and resistance to vibriosis and viral nervous necrosis in Atlantic cod (*Gadus morhua* L.). *Aquaculture*, **317**(1–4), 67–73. doi: 10.1016/j.aquaculture.2011.04.018.
131. Levraud JP, Jouneau L, Briolat V, Laghi V, Boudinot P. 2019. IFN-Stimulated Genes in Zebrafish and Humans Define an Ancient Arsenal of Antiviral Immunity. *The Journal of Immunology*, *203*(12), 3361–3373. doi: 10.4049/jimmunol.1900804.
132. Zhang R, Kang R, Tang D. 2021. The STING1 network regulates autophagy and cell death. *Signal Transduction and Targeted Therapy*, **6**(1), 208. doi: 10.1038/s41392-021-00613-4.
133. Feng H, Zhang YB, Zhang QM, Li Z, Zhang QY, Gui JF. 2015. Zebrafish IRF1 Regulates IFN Antiviral Response through Binding to IFNφ1 and IFNφ3 Promoters Downstream of MyD88 Signaling. *The Journal of Immunology*, *194*(3), 1225–1238. doi: 10.4049/jimmunol.1402415.

134. Sullivan C, Soos BL, Millard PJ, Kim CH, King BL. 2021. Modeling Virus-Induced Inflammation in Zebrafish: A Balance Between Infection Control and Excessive Inflammation. *Frontiers in Immunology*, **12**. doi: 10.3389/fimmu.2021.636623.

135. Zhou N, Chen LL, Chen J, Guo ZP. 2020. Molecular characterization and expression analysis of IL-1β and two types of IL-1 receptor in barbel steed (*Hemibarbus labeo*). *Comparative Biochemistry and Physiology Part B: Biochemistry and Molecular Biology*, *241*, 110393. doi: 10.1016/j.cbpb.2019.110393.

136. Joo MS, Choi KM, Kang G, Woo WS, Kim KH, Sohn MY, Son HJ, Han HJ, Choi HS, Kim DH, Park CI. 2022. Red sea bream interleukin (IL)-1β and IL-8 expression, subcellular localization, and antiviral activity against red sea bream iridovirus (RSIV). *Fish & Shellfish Immunology*, *128*, 360–370. doi: 10.1016/j.fsi.2022.07.040.

137. Marinis JM, Hutti JE, Homer CR, Cobb BA, Cantley LC, McDonald C, Abbott DW. 2012. IκB Kinase *α* Phosphorylation of TRAF4 Downregulates Innate Immune Signaling. *Molecular and Cellular Biology*, **32**(13), 2479–2489. doi: 10.1128/MCB.00106-12.

138. You SL, Jiang XX, Zhang GR, Ji W, Ma XF, Zhou X, Wei KJ. 2023. Molecular Characterization of Nine TRAF Genes in Yellow Catfish (*Pelteobagrus fulvidraco*) and Their Expression Profiling in Response to *Edwardsiella ictaluri* Infection. *International Journal of Molecular Sciences*, *24*(9), 8363. doi: 10.3390/ijms24098363.

139. Alvarez JM, Schinke AL, Brooks MD, Pasquino A, Leonelli L, Varala K, Safi A, Krouk G, Krapp A, Coruzzi GM. 2020. Transient genome-wide interactions of the master transcription factor NLP7 initiate a rapid nitrogen-response cascade. *Nature Communications*, **11**(1), 1157. doi: 10.1038/s41467-020-14979-6.

140. Weidemüller P, Kholmatov M, Petsalaki E, Zaugg JB. 2021.. Transcription factors: Bridge between cell signaling and gene regulation. *PROTEOMICS*, **21**(23–24). doi: 10.1002/pmic.202000034.

141. Katzenback, BA, Katakura F, Belosevic M. 2012. Regulation of Teleost Macrophage and Neutrophil Cell Development by Growth Factors and Transcription Factors. In *New Advances and Contributions to Fish Biology*. InTech. doi: 10.5772/53589.

142. Trizzino M, Zucco A, Deliard S, Wang F, Barbieri E, Veglia F, Gabrilovich D, Gardini A. 2021. EGR1 is a gatekeeper of inflammatory enhancers in human macrophages. *Science Advances*, *7*(3). doi: 10.1126/sciadv.aaz8836.

143. Azcoitia V, Aracil M, Martínez-A C, Torres M. 2005. The homeodomain protein Meis1 is essential for definitive hematopoiesis and vascular patterning in the mouse embryo. *Developmental Biology*, *280*(2), 307–320. doi: 10.1016/j.ydbio.2005.01.004.

144. Cvejic A, Serbanovic-Canic J, Stemple DL, Ouwehand WH. 2011. The role of meis1 in primitive and definitive hematopoiesis during zebrafish development. *Haematologica*, *96*(2), 190–198. doi: 10.3324/haematol.2010.027698.

145. Martínez de Paz A, Josefowicz SZ. 2021. Signaling-to-chromatin pathways in the immune system. *Immunological Reviews*, **300**(1), 37–53. doi: 10.1111/imr.12955.

146. Lesch BJ, Page DC. 2014. Poised chromatin in the mammalian germ line. *Development*, **141**(19), 3619–3626. doi: 10.1242/dev.113027.

147. Barbieri M, Xie SQ, Torlai-Triglia E, Chiariello AM, Bianco S, de Santiago I, Branco MR, Rueda D, Nicodemi M, Pombo A. 2017. Active and poised promoter states drive folding of the extended HoxB locus in mouse embryonic stem cells. *Nature Structural & Molecular Biology*, **24**(6), 515–524. doi: 10.1038/nsmb.3402.

148. Stępniak K, Machnicka MA, Mieczkowski J, Macioszek A, Wojtaś B, Gielniewski B, Poleszak K, Perycz M, Król SK, Guzik R et al. 2021. Mapping chromatin accessibility and active regulatory elements reveals pathological mechanisms in human gliomas. *Nature Communications*, **12**(1), 3621. doi: 10.1038/s41467-021-23922-2.

149. Saeed S, Quintin J, Kerstens HHD, Rao NA, Aghajanirefah A, Matarese F, Cheng SC, Ratter J, Berentsen K, van der Ent MA et al. 2011. Role of the Inflammasome, IL-1β,

and IL-18 in Bacterial Infections. *The Scientific World JOURNAL*, **11**, 2037–2050. doi: 10.1100/2011/212680.

150. Howe FS, Fischl H, Murray SC, Mellor J. 2017. Is H3K4me3 instructive for transcription activation? *BioEssays*, **39**(1), e201600095. doi: 10.1002/bies.201600095.

151. Bernard D, Riteau B, Hansen JD, Phillips RB, Michel F, Boudinot P, Benmansour A. 2006. Costimulatory Receptors in a Teleost Fish: Typical CD28, Elusive CTLA4. *The Journal of Immunology*, *176*(7), 4191–4200. doi: 10.4049/jimmunol.176.7.4191.

152. Zhang J, Wei X, Zhang Q, Jiao X, Li K, Geng M, Cao Y, Wang D, Cheng J, Yang J. 2024. Fish Uses CTLA-4 Immune Checkpoint to Suppress mTORC1-Controlled T-Cell Glycolysis and Immunity. *The Journal of Immunology*, *212*(7), 1113–1128. doi: 10.4049/jimmunol.2300599.

153. Ozato K, Shin DM, Chang TH, Morse HC. 2008. TRIM family proteins and their emerging roles in innate immunity. *Nature Reviews Immunology*, *8*(11), 849–860. doi: 10.1038/nri2413.

154. Langevin C, Levraud JP, Boudinot P. 2019. Fish antiviral tripartite motif (TRIM) proteins. *Fish & Shellfish Immunology*, *86*, 724–733. doi: 10.1016/j.fsi.2018.12.008.

155. Waterborg JH. 2012. Evolution of histone H3: emergence of variants and conservation of post-translational modification sites. *Biochemistry and Cell Biology*, *90*(1), 79–95. doi: 10.1139/o11-036.

156. Beacon TH, Delcuve GP, López C, Nardocci G, Kovalchuk I, van Wijnen AJ, Davie JR. 2021. The dynamic broad epigenetic (H3K4me3, H3K27ac) domain as a mark of essential genes. *Clinical Epigenetics*, *13*(1), 138. doi: 10.1186/s13148-021-01126-1.

157. Kundaje A, Meuleman W, Ernst J, Bilenky M, Yen A, Heravi-Moussavi A, Kheradpour P, Zhang Z, Wang J, Ziller MJ, Amin V, Whitaker JW, Schultz MD, Ward L. D, Sarkar A, Quon G, Sandstrom RS, Eaton ML, Wu YC, … Kellis M (2015). Integrative analysis of 111 reference human epigenomes. Nature, 518(7539), 317–330. doi: 10.1038/nature14248.

158. van der Velde A, Fan K, Tsuji J, Moore JE, Purcaro MJ, Pratt HE, Weng Z (2021). Annotation of chromatin states in 66 complete mouse epigenomes during development. Communications Biology, 4(1), 239. doi: 10.1038/s42003-021-01756-4.

159. Smale ST, Fisher AG. 2002. Chromatin Structure and Gene Regulation in the Immune System. *Annual Review of Immunology*, *20*(1), 427–462. doi: 10.1146/annurev.immunol.20.100301.064739.

160. Lim PS, Li J, Holloway AF, Rao S. 2013. Epigenetic regulation of inducible gene expression in the immune system. *Immunology*, *139*(3), 285–293. doi: 10.1111/imm.12100.

161. Watson DK, Li R, Sementchenko VI, Mavrothalassitis G, Seth A. 2002. ETS Family of Transcription Factors. In *Encyclopedia of Cancer* (pp. 189–196). Elsevier. doi: 10.1016/B0-12-227555-1/00078-2.

162. Turkistany SA, DeKoter RP. 2011. The Transcription Factor PU.1 is a Critical Regulator of Cellular Communication in the Immune System. *Archivum Immunologiae et Therapiae Experimentalis*, **59**(6), 431–440. doi: 10.1007/s00005-011-0147-9.

163. Chen S, Yang J, Wei Y, Wei X. 2020. Epigenetic regulation of macrophages: from homeostasis maintenance to host defense. *Cellular & Molecular Immunology*, **17**(1), 36–49. doi: 10.1038/s41423-019-0315-0.

164. Shan S, Liu R, Jiang L, Zhu Y, Li H, Xing W, Yang G. 2018. Carp Toll-like receptor 8 (Tlr8): An intracellular Tlr that recruits TIRAP as adaptor and activates AP-1 pathway in immune response. *Fish & Shellfish Immunology*, *82*, 41–49. doi: 10.1016/j.fsi.2018.08.001.

165. Kim J, Lee KW, Lee HJ. 2014. Polyphenols Suppress and Modulate Inflammation. In *Polyphenols in Human Health and Disease* (pp. 393–408). Elsevier. doi 10.1016/B978-0-12-398456-2.00029-3.

166. Clark TC, Boudinot P, Collet B. 2021. Evolution of the IRF Family in Salmonids. *Genes*, **12**(2), 238. doi: 10.3390/genes12020238.
167. Han C, Huang W, Peng S, Zhou J, Zhan H, Li W, Gong J, Li Q. 2023. Characterization and expression analysis of the interferon regulatory factor (IRF) gene family in zig-zag eel (*Mastacembelus armatus*) against *Aeromonas veronii* infection. *Developmental & Comparative Immunology*, **140**, 104622. doi: 10.1016/j.dci.2022.104622.
168. Yan X, Zhao X, Huo R, Xu T. 2020b. IRF3 and IRF8 Regulate NF-κB Signaling by Targeting MyD88 in Teleost Fish. *Frontiers in Immunology*, **11**. doi: 10.3389/fimmu.2020.00606.
169. Ippolito GC, Dekker JD, Wang YH, Lee BK, Shaffer AL, Lin J, Wall JK, Lee BS, Staudt LM, Liu YJ et al. 2014. Dendritic cell fate is determined by BCL11A. *Proceedings of the National Academy of Sciences*, **111**(11). doi: 10.1073/pnas.1319228111.
170. Yu Y, Wang J, Khaled W, Burke S, Li P, Chen X, Yang W, Jenkins NA, Copeland NG, Zhang S et al. 2012. Bcl11a is essential for lymphoid development and negatively regulates p53. *Journal of Experimental Medicine*, **209**(13), 2467–2483. doi: 10.1084/jem.20121846.
171. Cabrera-Ortega AA, Feinberg D, Liang Y, Rossa C, Graves DT. 2017. The Role of Forkhead Box 1 (FOXO1) in the Immune System: Dendritic Cells, T Cells, B Cells, and Hematopoietic Stem Cells. *Critical Reviews in Immunology*, **37**(1), 1–13. doi: 10.1615/CritRevImmunol.2017019636.
172. Graves DT, Milovanova TN. 2019. Mucosal Immunity and the FOXO1 Transcription Factors. *Frontiers in Immunology*, **10**. doi: 10.3389/fimmu.2019.02530.
173. Jo SS and Choi SS. 2019. Analysis of the Functional Relevance of Epigenetic Chromatin Marks in the First Intron Associated with Specific Gene Expression Patterns. Genome Biology and Evolution, 11(3), 786–797. doi: 10.1093/gbe/evz033.
174. Johnston AD, Simões-Pires CA, Thompson Tv, Suzuki M, Greally JM. 2019. Functional genetic variants can mediate their regulatory effects through alteration of transcription factor binding. Nature Communications, 10(1), 3472.

 Click here to access/download;Table;Main tables.docx

Table 1. Number of differentially expressed genes (DEGs) for the *in vitro* and *in vivo* stimulations with *Vibrio* and poly I:C

Stimulant	Stimulation	Downregulated DEGs	Upregulated DEGs	Total DEGs
Vibrio	*in vitro*	3,217	3,321	6,538
	in vivo	929	910	1,839
poly I:C	*in vitro*	544	858	1,402
	in vivo	1,918	2,001	3,919

Table 2. Immune-related DEGs showing opposite responses following *Vibrio* and poly I:C stimulations.

Comparison	Differentially expressed genes (DEGs)
In vitro - upregulated by poly I:C, downregulated by *Vibrio*	***aldh2-like***, ***arhgap22***, <u>*arrdc2*</u>, <u>*asb9-like*</u> ***ascc3***, ***atxn7l1***, *bpifcl*, *c1qa*, ***cep170b***, *chaf1b*, *cnnm4b*, ***dync1h1***, ***ece2b***, ***ermp1-like***, ***gna14***, ***gpr155a***, ***has1***, ***ip6k2-like***, ***mapk8a***, ***mycbp2***, *nfkb1*, ***notch2***, *pld4-like*, *psda*, *rilp*, *serinc1-like*, *sgk2b*, *sp100.1*, ***spsb4a***, ***sptbn1***, *stim2b*, *synpr*, ***tank***, *tent4b*, ***tlr8***, *tmem269-like*, *tnk2b*, *tpm4b*, ***trim25-like***, ***trim35-14***, ***usp1***, *vcanb*
In vitro - upregulated by *Vibrio*, downregulated by poly I:C	*bcat-like*, <u>*ctns*</u>, *gid8b-like*, *pcxb*, ***psat1***, *rab11b-like*, *rtf2*, *sfxn2*, ***slc25a43***, ***stat3***, *thoc7*, *tollip*, *vhl*, *vps26c*, <u>*vps41*</u>
In vivo - upregulated by poly I:C, downregulated by *Vibrio*	*adora4a*, ***ankrd10b***, ***apaf1***, <u>*arhgap12b*</u>, *atg9a*, ***atxn7l1***, *bpifcl*, *camkk1b*, ***casp1a-like***, *cylda*, *emilin1*, ***fem1c***, *gfral*, ***gna14***, *gnptab*, <u>*il22ra2*</u>, ***irf1b***, <u>*nlrc3-like*</u>, ***nod2***, <u>*nsmce4a*</u>, *otulina*, ***parp15-like***, ***pstpip1b***, ***pstpip2***, ***rasgef1b***, *rnf146*, ***rnf170***, ***sting1***, ***themis2***, ***txk***, *ubp15*, <u>*ucp2-like*</u>, ***xcr1-like***, ***xkr8-like***
In vivo - upregulated by *Vibrio*, downregulated by poly I:C	*aebp1*, *alpl*, *anxa13l*, <u>*anxa1a*</u>, <u>*cald1a*</u>, *capn2b*, *chchd6a*, *col17a1-like*, <u>*f2r*</u>, <u>*ggh-like*</u>, *icn-like*, *il-1b*, <u>*il8-like*</u>, <u>*il11-like*</u>, *kdm2aa*, <u>*lims2*</u>, *map1lc3b*, *mfsd10*, *msto1*, *olfm5-like*, *osbpl10*, *plpp1like*, *plxnb2b*, <u>*slc4a4-like*</u>, *tlr13-like*, <u>*traf4a*</u>, <u>*vat1*</u>, <u>*zan-like*</u>

Turbot orthologs of human / mouse type I IFN stimulated genes (ISG) are highlighted (in bold upregulated and underlined downregulated), based on the database Interferome (Rusinova et al., 2013) and Clark et al. (2023). Note that most of these genes are upregulated in response to Poly I:C stimulation, but downregulated in response to Vibrio stimulation.

Table 3. Differential Accessibility Regions (DARs) and Differential Histone Modification Regions (DHMRs) for the *in vitro* and *in vivo* immune stimulations with viral and bacterial PAMPs

PAMP	Stimulation	Assay	Downregulated DAR / DHMR	Upregulated DAR / DHMR	All DAR / DHMR
Vibrio	*in vitro*	ATAC	2,739	17,878	20,617
		H3K4me3	699	10,755	11,454
		H3K27ac	2	623	625
		H3K27me3	6	1,211	1,217
	in vivo	ATAC	43,159	16,733	59,892
		H3K4me3	287	9,988	10,275
		H3K27ac	0	3	3
		H3K27me3	4	0	4
poly I:C	*in vitro*	ATAC	0	0	0
		H3K4me3	0	0	0
		H3K27ac	0	20	20
		H3K27me3	0	0	0
	in vivo	ATAC	25	15	40
		H3K4me3	0	1,036	1,036
		H3K27ac	0	38	38
		H3K27me3	126	0	126

Table 4. Overlap of promoter DARs and DHMRs with DEG promoters (Hypergeometric test, $p < 0.05$). Significant results are underlined. Refer to **Supplementary table 11** for further details.

Experimental condition	Integration of DARs / DHMRs at promoter regions of DEGs

	DARs/DHMRs at promoter regions		Down		Up	
	**Down**	**Up**	**DEG**	**DAR/DHMR + DEG**	**DEG**	**DAR / DHMR + DEG**
ATAC poly I:C *in vitro*	-	-	544	-	858	-
ATAC *Vibrio in vitro*	55	4922	3217	<u>1</u>	3321	<u>1259</u>
ATAC poly I:C *in vivo*	4	1	1918	-	2001	-
ATAC *Vibrio in vivo*	1350	6115	929	34	910	<u>395</u>
H3K4me3 poly I:C *in vitro*	-	-	544	-	858	-
H3K4me3 *Vibrio in vitro*	53	4289	3217	<u>14</u>	3321	<u>1164</u>
H3K4me3 poly I:C *in vivo*	-	368	1918	-	2001	<u>151</u>
H3K4me3 *Vibrio in vivo*	22	4024	929	<u>3</u>	910	<u>242</u>
H3K27ac poly I:C *in vitro*	-	-	544	-	858	-
H3K27ac *Vibrio in vitro*	-	72	3217	-	3321	<u>49</u>
H3K27ac poly I:C *in vivo*	-	4	1918	-	2001	<u>3</u>
H3K27ac *Vibrio in vivo*	-	1	929	-	910	<u>1</u>
Active prom poly I:C *in vitro*	-	-	544	-	858	-
Active prom *Vibrio in vitro*	106	6744	3217	15	3321	<u>1782</u>
Active prom poly I:C *in vivo*	4	370	1918	-	2001	<u>151</u>
Active prom *Vibrio in vivo*	1263	7082	929	37	910	<u>463</u>

prom: promoter; DAR: differential accessibility regions; DHMR: differential histone modification regions

Figure 1

Click here to access/download;Figure;Figure_1_Experimental_design.tiff

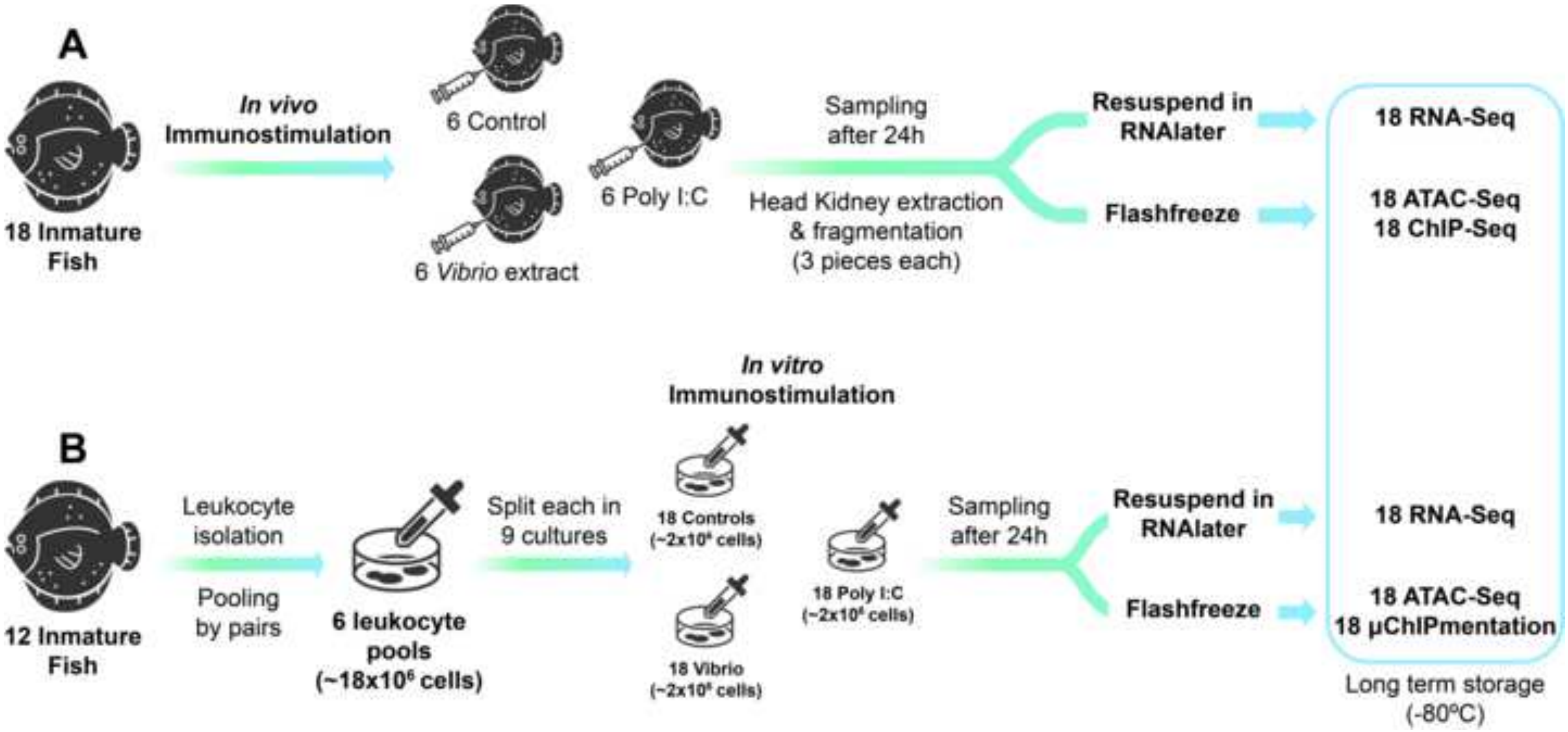

Figure 2

Click here to access/download;Figure;Figure_2_PCA_transcriptome.tiff

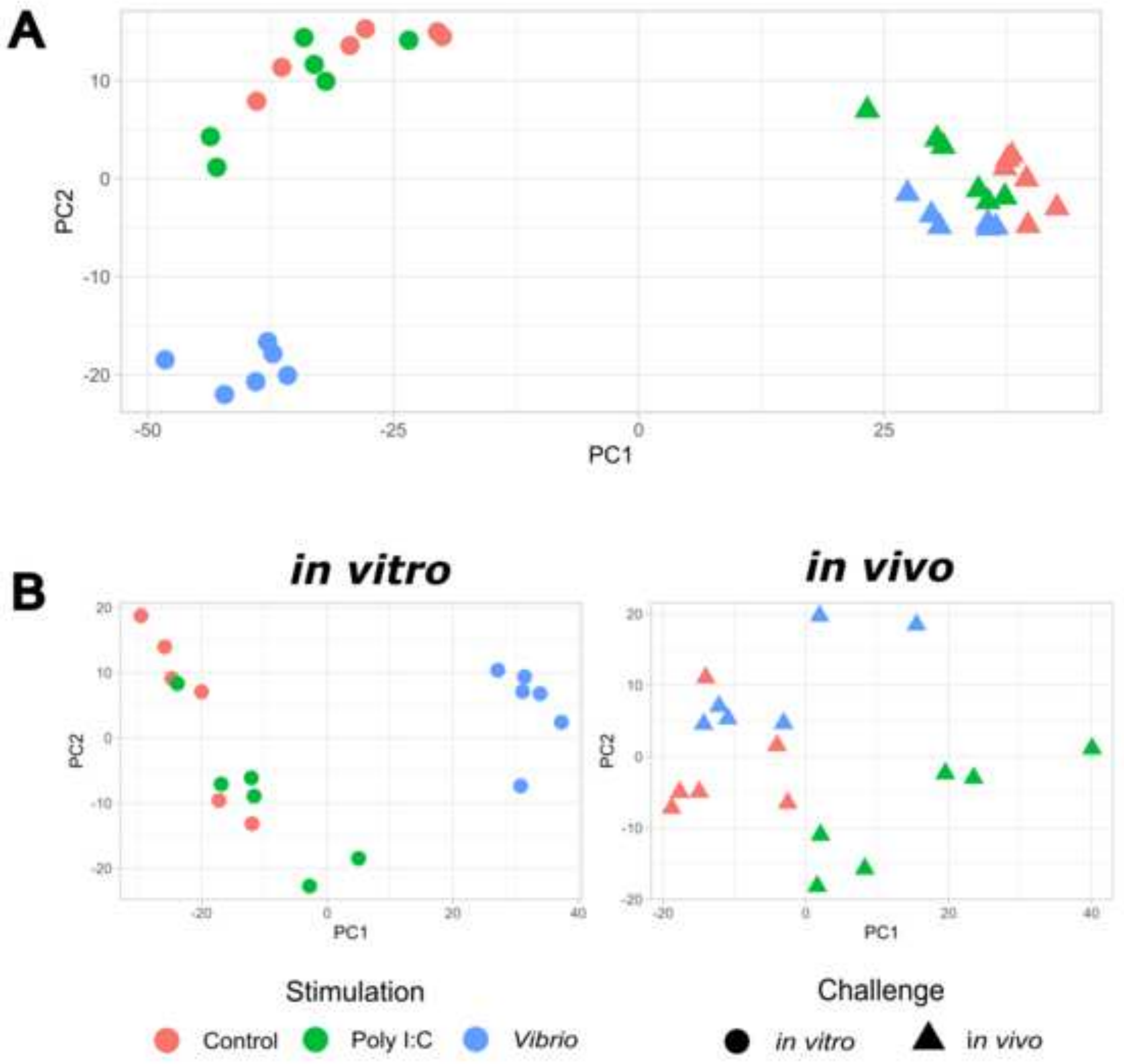

Figure 3

Click here to access/download;Figure;Figure_3_venn_comparissons.tiff

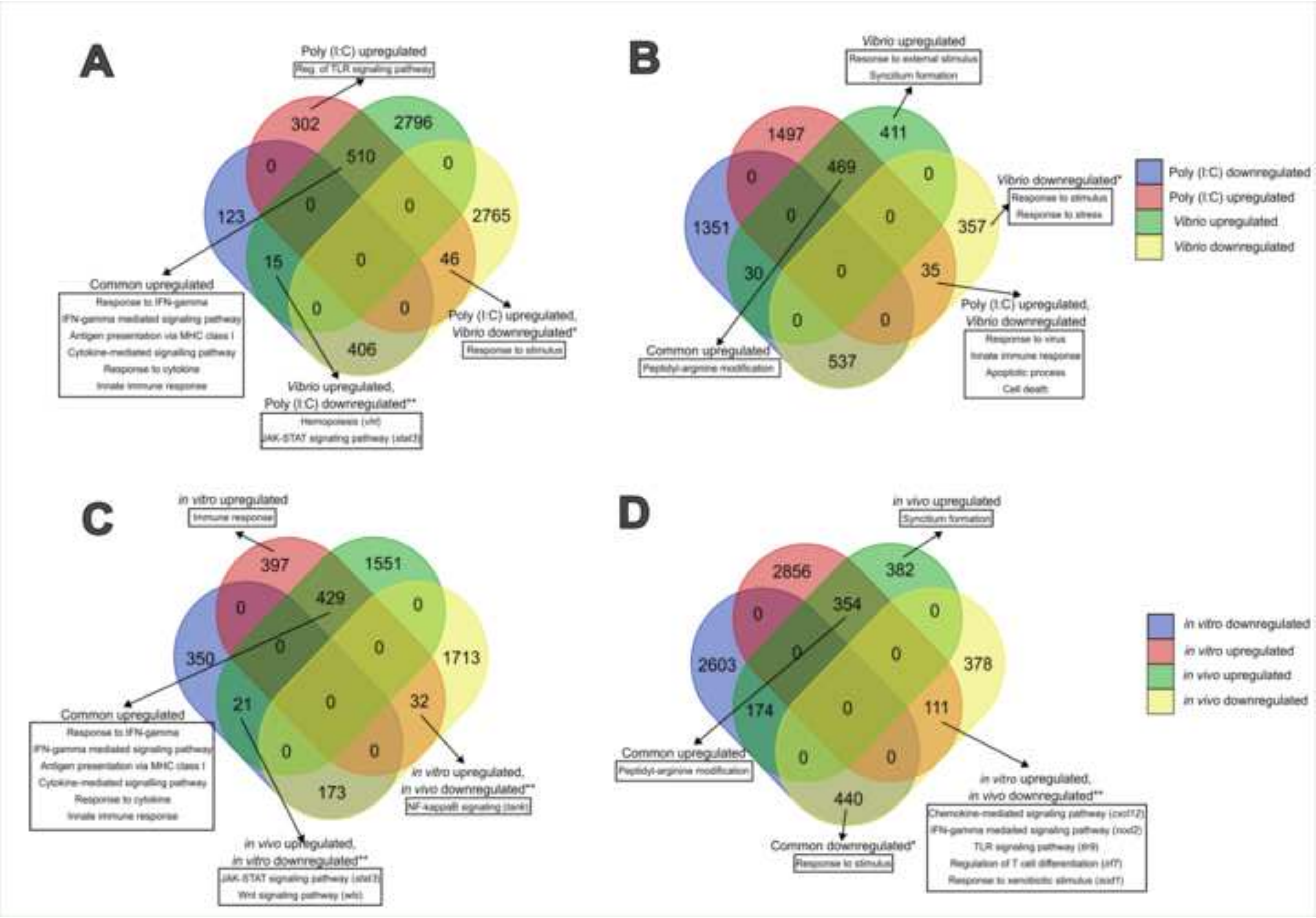

Figure 4

Click here to access/download;Figure;Figure_4_Heatmap_Spearman.tiff

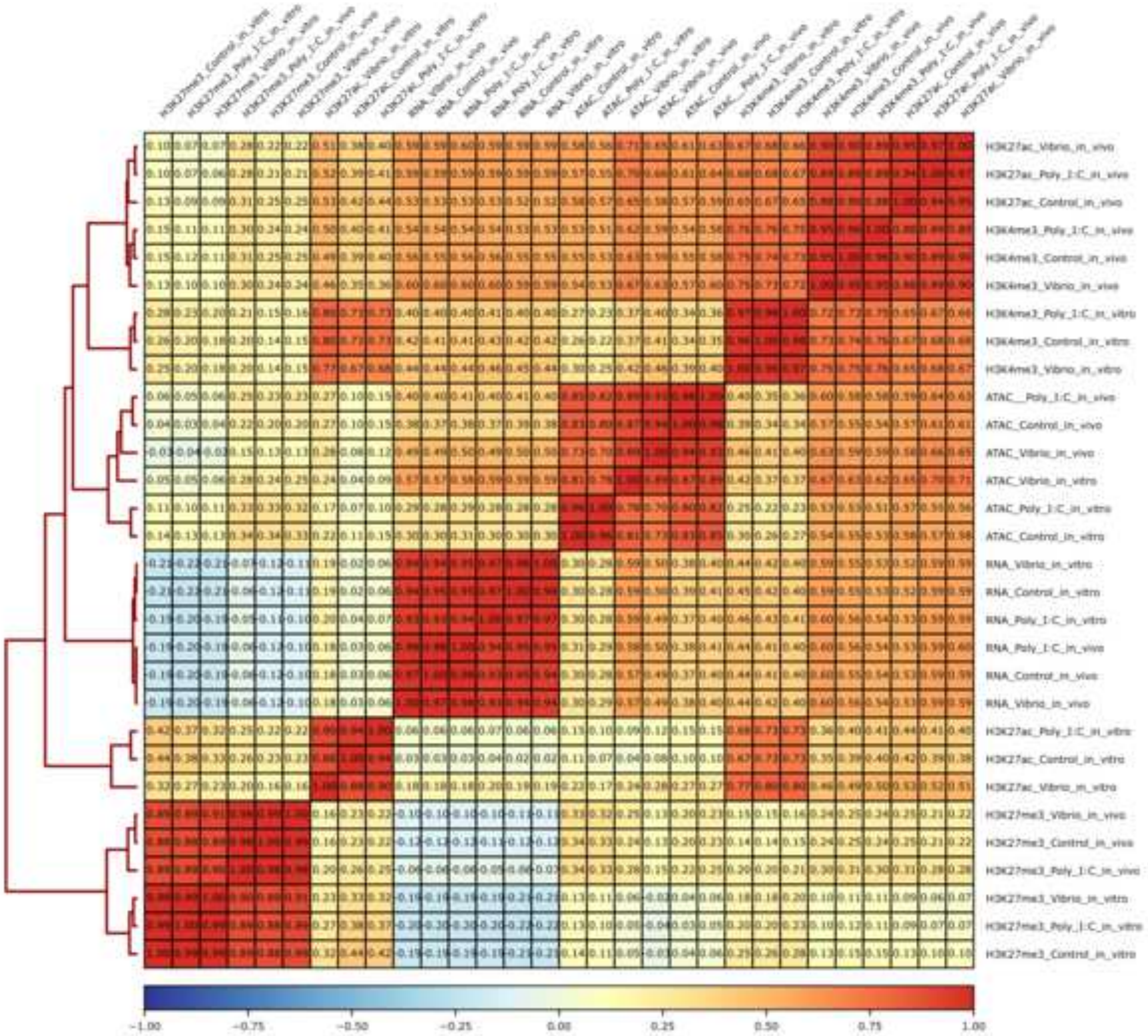

Figure 5

Click here to access/download;Figure;Figure_5_Chromatin_states.tiff

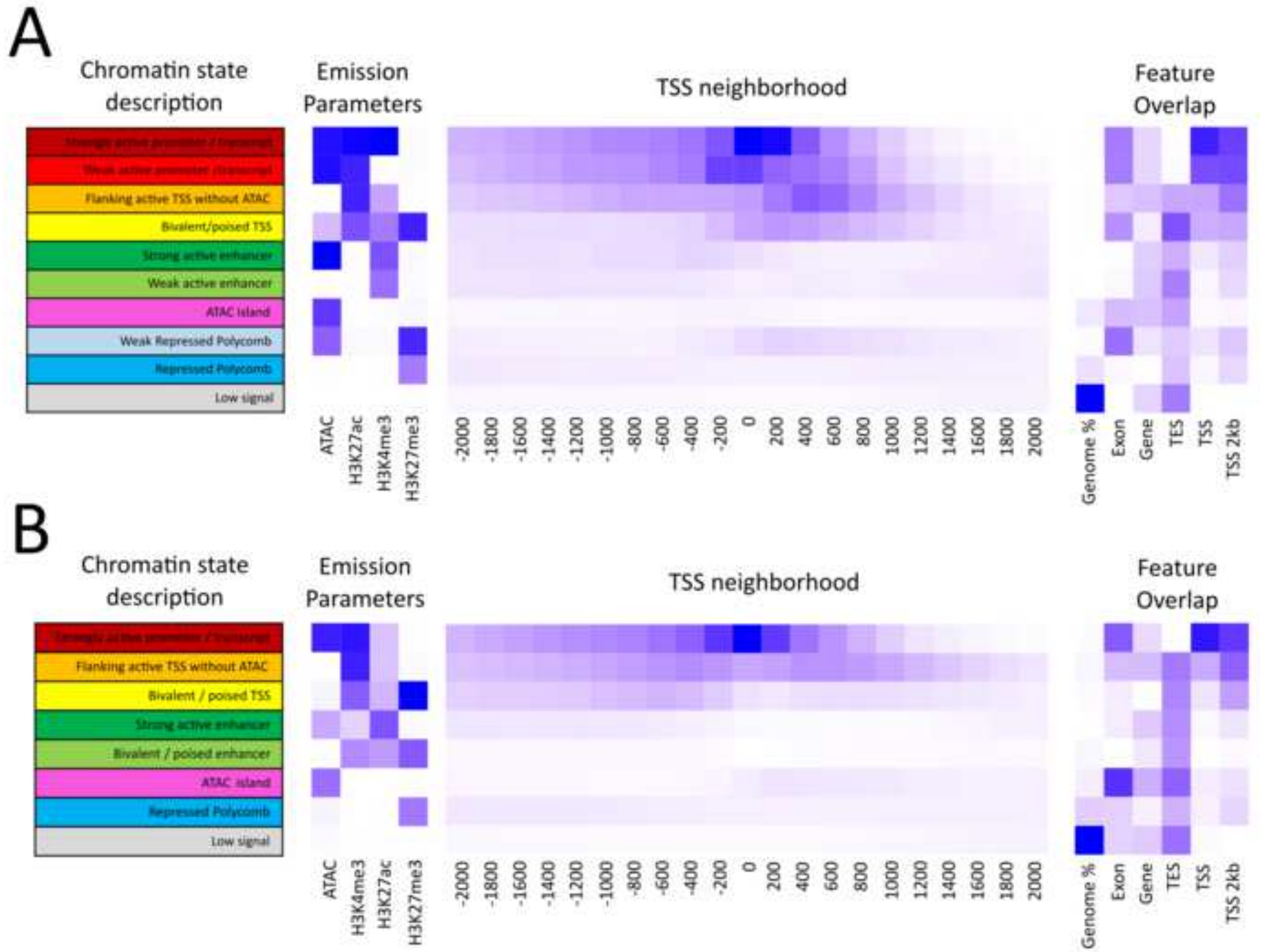

Figure 6

Click here to access/download;Figure;Figure_6_UpsetPlot_TFBM.tiff

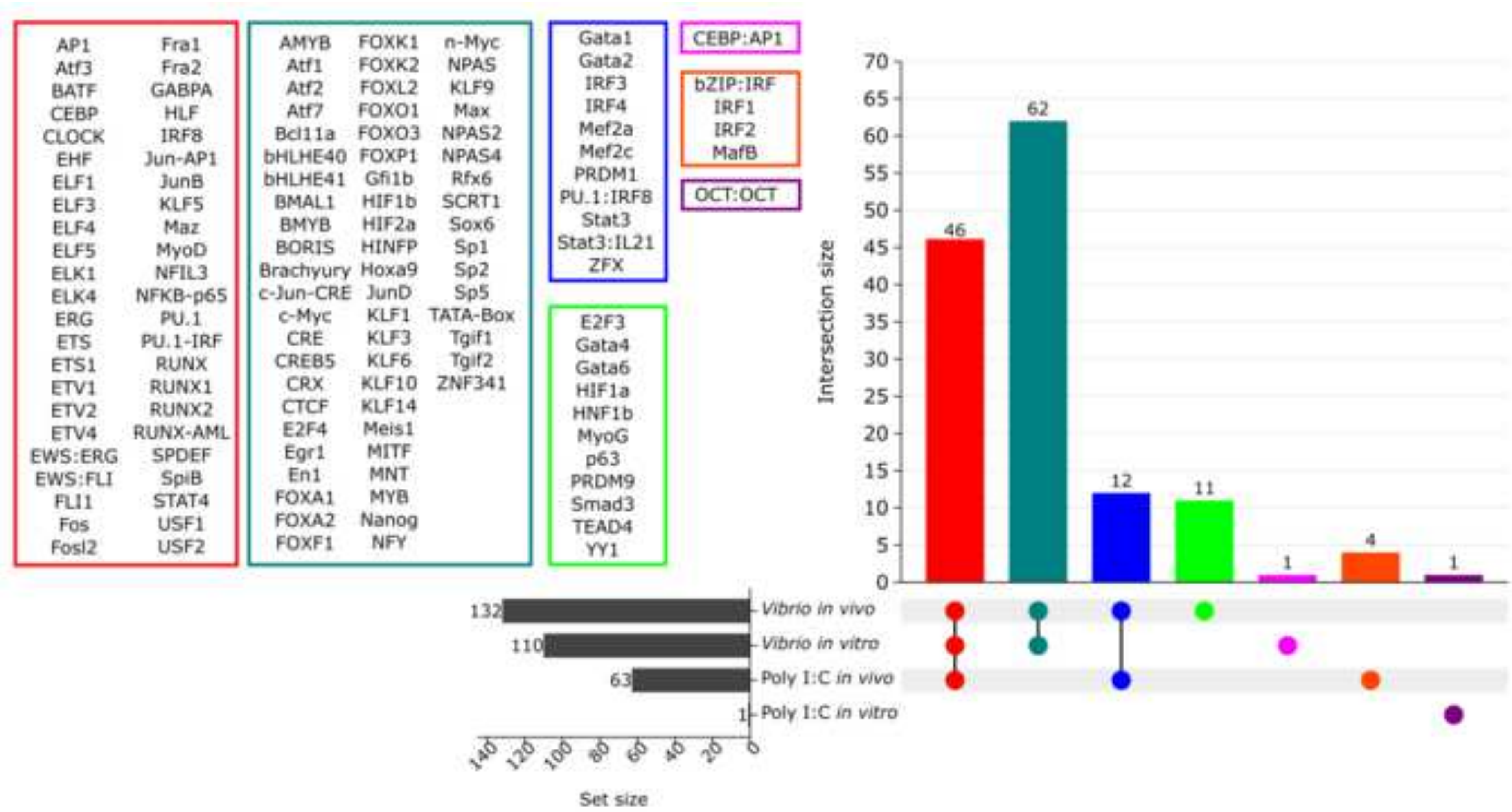

Figure 7

Click here to access/download;Figure;Figure_7_FOXO1A.tiff

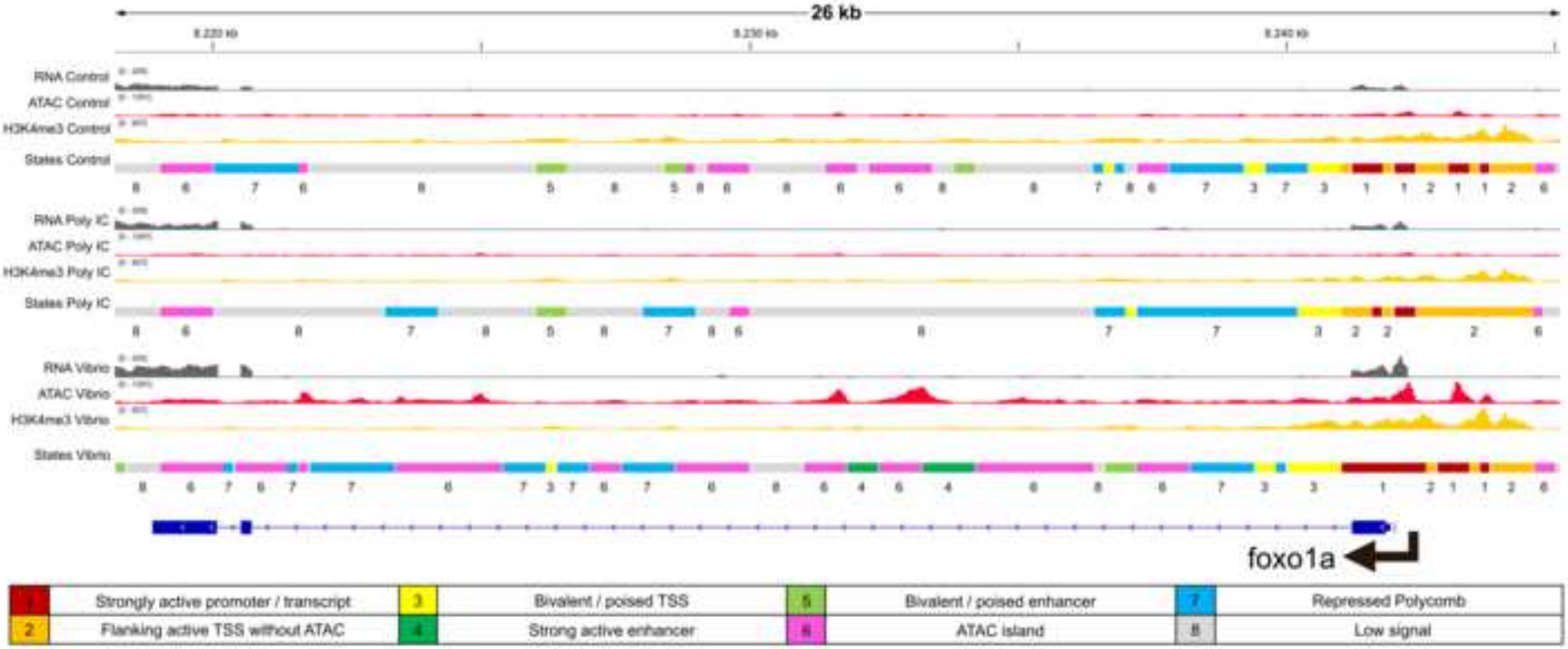

Main text figure descriptions

Click here to access/download
Supplementary Material
Main figure descriptions.docx

Supplementary Figures and descriptions

Click here to access/download
Supplementary Material
Supplementary Figures v2.pdf

Supplementary table 1

Click here to access/download
Supplementary Material
Supplementary_table_1_Sample_metadata_revised.xlsx

Supplementary table 2

Click here to access/download
Supplementary Material
Supplementary_table_2_Library_metadata_revised.xlsx

Supplementary table 3

Click here to access/download

Supplementary Material

Supplementary_table_3_QC_data_revised.xlsx

Supplementary table 4

Click here to access/download
Supplementary Material
Supplementary_table_4_DEG.xlsx

Supplementary table 5

Click here to access/download
Supplementary Material
Supplementary_table_5_GO_analysis.xlsx

Supplementary table 6

Click here to access/download
Supplementary Material
Supplementary_table_6_GO_analysis_comparissons.xls
x

Supplementary table 7

Click here to access/download
Supplementary Material
Supplementary_table_7_NEW.docx

Supplementary table 8

Click here to access/download
Supplementary Material
Supplementary_table_8_blacklist.xlsx

Supplementary table 9

Click here to access/download
Supplementary Material
Supplementary_table_9_Chromatin states.xlsb

Supplementary table 10

Click here to access/download
Supplementary Material
Supplementary_table_10_DiffBind_annotated.xlsx

Supplementary table 11

Click here to access/download
Supplementary Material
Supplementary_table_11_Summary_DEG_DAR_DHMR_revised.xlsx

Supplementary table 12

Click here to access/download
Supplementary Material
Supplementary_table_12_GO_DEG_DAR_DHMR.xlsx

Supplementary table 13

Click here to access/download
Supplementary Material
Supplementary_table_13_TF_motifs_revised.xlsx

Supplementary table 14

Click here to access/download
Supplementary Material
Supplementary_table_14_TF_genes_revised.xlsx
