# Supplementary material for: Multiomics uncovers the epigenomic and transcriptomic response to viral and bacterial stimulation in turbot
Source: Gigascience. 2025 Jul 15;14:giaf077. doi: 10.1093/gigascience/giaf077 (PMC12263217; doi:10.1093/gigascience/giaf077)
Supplement: giaf077_Authors_Response_To_Reviewer_Comments_original_submission [file giaf077_authors_response_to_reviewer_comments_original_submission.pdf]

Author's Response To Reviewer Comments

In the following section we answer the specific comments made by each of the reviewer. Each comment in the decision letter that required input by the authors is referenced by the numeration and / or the title of the section provided by the reviewer, with the answers by the authors signales as "Answer:". Thanks for your time.

Answers to reviewer #1:

Issue 1

Answer: We have noticed that we incorrectly annotated the population size (N) as the total list of genes that were DEG, DAR or DHMR. Apologies for the inconsistency that made the revision more difficult, this has been corrected now, as the population size (N) is the total list of genes in the annotated turbot genome (ensembl) that are located in chromosomes (this is, excluding mitochondrial genes and genes found in unassigned scaffolds). We have added this clarification to the "Pipeline_description_and_additional_codes.txt" file.

Issue 2

Answer: Yes, apologies for the confusion with the formatting of supplementary table 4. We have added the next lines on supplementary table 4 that makes this clear: "The simbol on column "log2FoldChange" denotes the direction of the differential expression, with negative values (-) refering to genes upregulated in the control condition, and positive values refering to genes upregulated in the tested condition (either Poly I:C or Vibrio)".

Issue 3

Answer: Apologies, we have revised this section and added some changes to supplementary table 10 to make the retrieval of that information more straightforward, as well as clarifying the steps in the "Pipeline_description_and_additional_codes.txt" file.
First, to fill the "all DAR/DHMR" section on columns B and C of supplementary table 11, we have to go to supplementary table 10, where we have sheets in the same format for each chromatin technique (ATAC-seq and ChIP-seq for H3K4me3 and H3K27ac; we ignore H3K27me3 for the hypergeometric test). To retrieve the number of DAR/DHMR, stablish filters for columns A and B: Column A designates the comparison that DAR/DHMR stems from (in vitro Vibrio, in vivo Vibrio, in vitro Poly (I:C) or in vivo Poly (I:C), while column B shows whether the DAR/DHMR is upregulated (positive in the tested condition, either Vibrio or Poly (I:C) or downregulated (positive in the control condition). Filtering by these two columns lets us select the specific dataset we want to retrieve for columns B and C in supplementary table 11.
Second, to fill the "DAR/DHMR at promoter regions", we must stablish first a filter in column T of supplementary table 10 and set it to "promoter-TSS". Then, replicate the filters for columns A and B described on the first paragraph to iterate through the different assays and tested conditions, filling the data for columns E and F in supplementary table 11 for the ATAC, H3K4me3 and H3K27ac assays. To ease the reproduction up to this step, we have added the results of this step in an additional subsection to supplementary table 10 (10E) that gathers all the DAR/DHMR at promoter regions in one single table.
The third step is to fill in the rows corresponding to promoters-vitro-vibrio, promoters-vivo-poly, promoters-vivo-vibrio on columns E and F, which gather information from all active regulatory mark differential peaks (ATAC, H3K4me3 and H3K27ac(that land on promoters. For this, deriving from the new supplementary table 10E, create six additional sheets, one for each combination of Group (column B) and Direction (column C): in vitro Vibrio Upregulated, in vitro Vibrio Downregulated, in vivo Poly (I:C) Upregulated, in vivo Poly (I:C) Downregulated, in vivo Vibrio Upregulated, and in vivo Vibrio Downregulated. Copy and paste the content on supplementary table 10E to each of them and remove all rows that don't belong to the corresponding Group and Direction. Finally, go to the Annotation column (column N) and remove duplicates for each sublist. This will give a list of promoters that have differential activation signals across conditions, allowing to fill the missing data of columns E and F on supplementary

table 11. For each of this sublists, the contents of columns A to M, and Q and R can be removed. To ease the reproduction up to this step, we have added six new sheets to supplementary table 10 (10F to 10K) with the list of promoters with activation signals.

Issue 4

Answer: Apologies, we have replicated this issue and it in fact reports a “Request Entity Too Large” error that was not happening at the time of running these analyses. Please consider using the following tool: https://www.interactivenn.net/
We have tested this tool and it can replicate the results we got from the original one. Therefore, we have added it as an alternative for retrieving the intersections of DAR and DEG in the “Pipeline_description_and_additional_codes.txt” file.
Following the same principle as the previous venn diagram tool, here we would paste different lists of DEGs and promoter-DAR/DHMRs, retrieve the number of intersections from the generated figure (Should be the intersection between “[Set A] and [Set B]”) and, if needed, export the list of genes in the intersection in .txt format.

Issue 5

Answer: The population size is the total number of genes annotated in ensembl that are found in chromosomes, therefore excluding mitochondrial and scaffold genes. These can be retrieved from ensembl BioMart, by selecting the “Ensembl Genes 113”, “Turbot genes (ASM1334776v1)” and, in the attributes section, mark only the “Gene stable ID” and “Chromosome/scaffold name” tags, then click on results and export in desired format Then, filter out all the genes where the “Chromosome/scaffold name“ is MT or JABGBL, to remove the mitochondrial and unassigned genes, and retain only the 28,602 genes in chromosomes. We have added this clarification to the “Pipeline_description_and_additional_codes.txt” file.
Regarding supplementary table 4, yes, the “Gene ID (ENSEMBL)” column should be used to retrieve the DEG for the intersection between DAR/DHMR + DEG. For the DAR/DHMR in supplementary table 10, that could be extracted from the “Annotation” column but limiting it to only the “promoter-TSS” peaks and keeping only the ensemblIDs. As per the previous comment, we have added some modifications to this table to ease the transition towards the hypergeometric test on supplementary table 11. Therefore, it is only needed to retrieve the content on column “Gene ID (ENSEMBL)” of supplementary tables 10F to 10K to do the intersection between DAR/DHMR + DEG. We have added this clarification to the “Pipeline_description_and_additional_codes.txt” file.

Issue 1: Comparison of p-values

Answer: This is correct, we have added a warning on this section in the “Pipeline_description_and_additional_codes.txt” document.

5.4 Statistical comparison Original vs Reproduced results

Comments: The full table could not be reproduced, particularly the data corresponding to DAR/DHMR, DAR/DHMR+DEG and population size values, due to missing information or unclear definitions in the supplementary files.

Answer: We hope that the issues with reproducibility have been solved with the modifications we reported above.

Errors detected: The enrichment value for the Up condition of promoters-vitro-vibrio was incorrectly reported in the manuscript/table. Based on the Excel formula and the online tool used, the correct value appears to be 2.28 instead of 2.82.

Answer: Apologies, this was incorrectly reported. We have modified supplementary table 11 with the correct value “2.28”.

Statistical Consistency: All the values that could be reproduced from the available data matched the

original results, except for the detected error.

Answer: Amended

Improve data documentation

Answer: Key variables on supplementary tables 4, 10 and 11 have been defined.

Provide all code and scripts

Answer: Although no specific excel formulas were used for supplementary tables 4, 10 and 11, we have added a detailed description of it in the “Pipeline_description_and_additional_codes.txt”.

Clarify statistical methodology

Answer: Amended, see the corresponding section in the “Pipeline_description_and_additional_codes.txt” document.

Enhance reproducibility workflow

Answer: We have improved the readability of our original “Pipeline_description_and_additional_codes.txt” file and extended the explanation on how to carry out the hypergeometric test of supplementary table 11.

Answers to reviewer #2:

1. Typo in Figure 1 (it's immature, not inmature)

Answer: Amended

2. In Fig 3 Upset plots could be a bit easier to parse

Answer: The venn diagrams have been substituted with UpSet plots. Therefore, changes have been made to the Figure 3 description and the "Pipeline_description_and_additional_codes.txt" file.

3. Fig 5 doesn't have a legend for the blue gradient (but it's pretty self-explanatory)

Answer: Amended

Answers to reviewer #3:

Line 16

Answer: We have made the sentence more generalistic by removing the mention to innate immunity.

Line 34-36

Answer: We have clarified that our study is based on mimics of viral and bacterial stimulation in the abstract, and removed the sentence specified by the reviewer.

Line 80-87

Answer: We have improved the final paragraph on the introduction to better reflect the functions of the head kidney in fish, while maintaining our focus on immune processes.

Cookie Preference Center

We use cookies which are necessary to make our site work. We may also use additional cookies to analyze, improve and personalize our content and your digital experience. For more information, see our Cookie Policy.

You may choose not to allow some types of cookies. However, blocking some types may impact your experience of our site and the services we are able to offer. See the different category headings below to find out more or change your settings.

Allow all

Manage Consent Preferences

Strictly Necessary Cookies

Always active

These cookies are necessary for the website to function and cannot be switched off in our systems. They are usually only set in response to actions made by you which amount to a request for services, such as setting your privacy preferences, logging in or filling in forms. You can set your browser to block or alert you about these cookies, but some parts of the site will not then work.

Functional Cookies

These cookies enable the website to provide enhanced functionality and personalisation. They may be set by us or by third party providers whose services we have added to our pages. If you do not allow these cookies then some or all of these services may not function properly.

Performance Cookies

These cookies allow us to count visits and traffic sources so we can measure and improve the performance of our site. They help us to know which pages are the most and least popular and see how visitors move around the site.

Targeting Cookies

These cookies may be set through our site by our advertising partners. They may be used by those companies to build a profile of your interests and show you relevant adverts on other sites. If you do not allow these cookies, you will experience less targeted advertising.

Cookie List

Clear

Apply Cancel

Consent Leg.Interest

Confirm my choices

Powered by onetrust
