# Supplementary material for: Multiomics uncovers the epigenomic and transcriptomic response to viral and bacterial stimulation in turbot
Source: Gigascience. 2025 Jul 15;14:giaf077. doi: 10.1093/gigascience/giaf077 (PMC12263217; doi:10.1093/gigascience/giaf077)
Supplement: giaf077_Reviewer_1_Report_original_submission — Laura Caquelin -- 3/12/2025 [file giaf077_reviewer_1_report_original_submission.pdf]

Reviewer Report

Title: Multiomics uncovers the epigenomic and transcriptomic response to viral and bacterial stimulation in turbot

Version: Original Submission **Date:** 3/12/2025

Reviewer name: Laura Caquelin

Reviewer Comments to Author:

Reproducibility report for: Multiomics uncovers the epigenomic and transcriptomic response to viral and bacterial stimulation in turbot
Journal: GigaScience
ID number/DOI: GIGA-D-25-00052
Reviewer(s): Laura Caquelin, Department of Clinical Neuroscience, Karolinska Institutet, Sweden
1. Summary of the Study
This study provides the first multi-omics investigation of the innate immune response in turbot (Scophthalmus maximus). By integrating RNA-Seq, ATAC-Seq, and ChIP-Seq data, researchers identified changes in gene expression, chromatin accessibility, and histone modifications after viral and bacterial stimulation. The findings reveal a significant coupling between the transcriptome and chromatin state, offering insights for the selection of disease resistance in aquaculture.
2. Scope of reproducibility
According to our assessment the primary objective is: Association of ATAC-Seq and ChIP-Seq data with RNA-Seq data
â—� Outcome: Overlap of promoter DARs and DHMRs with DEG promoters
â—� Analysis method outcome: Hypergeometric test
â—� Main result: "DARs and DHMRs were much more overrepresented at the promoter regions of upregulated rather than downregulated DEGs" (Table 4, Supplementary Table 11; Lines 403-405, Page 9)
3. Availability of Materials
a. Data
â—� Data availability: Raw data are available, but generated data from the study are shared with the journal and not yet publicly available
â—� Data completeness: Complete
â—� Access Method: Manuscript's supplementary files/Private journal dropbox
â—� Repository: -
â—� Data quality: Structured, but lacks variable definitions in supplementary files, making it difficult to interpret and use.
b. Code
â—� Code availability: Not available for the primary result
â—� Programming Language(s): Excel
â—� Repository link: -
â—� License: -

â— Repository status: -
â— Documentation: README lacks information on hypergeometric test.
4. Computational environment of reproduction analysis
â— Operating system for reproduction: MacOS 14.7.4
â— Programming Language(s): Excel
â— Code implementation approach: Excel formulas based on methodology description provided by authors
â— Version environment for reproduction: Excel version 16.94
5. Results
5.1 Original study results
â— Results 1: Table 4 and supplementary table 11
5.3 Steps for reproduction
ïƒ¨ Reproduce supplementary table 11 to perform hypergeometric test
* Issue 1: No code or instructions for constructing Table 4 in manuscript and README text.
â–ª Resolved: Authors shared methodology upon request
Authors' Clarification: The hypergeometric test wasn't carried out with any particular script but with the following public online tool, that can be replicated in excel:
https://systems.crump.ucla.edu/hypergeometric/
The tool basically runs the following excel formulas:
Cumulative distribution function (CDF) of the hypergeometric distribution in Excel
=IF(k>=expected,1-HYPGEOM.DIST(k-1,s,M,N,TRUE),HYPGEOM.DIST(k,s,M,N,TRUE))
=IF(k>=((s*M)/N),1-HYPGEOM.DIST(k-1,s,M,N,TRUE),HYPGEOM.DIST(k,s,M,N,TRUE))
expected
= (s*M)/N
direction
=IF(k=expected,"match",IF(k<expected,"de-enriched","enriched"))
fold change
=IF(k<expected,expected/k,k/expected)
where k is the number of successes (intersection of DAR/DHMR in promoters + DEG), s the sample size (DEG), M the number of successes in the population (DAR/DHMR in promoters) and N the population size (28.602 genes).
For each condition, the count of downregulated and upregulated DEG (s) was taken from supplementary table 4. Similarly, the count of downregulated and upregulated DAR/DHMR (M) was taken from supplementary table 10, considering only differential peaks that are annotated as "promoter-TSS" in the annotation column (column M).
The population size (N) was the total list of genes that were DEG, DAR or DHMR (combining the data on supplementary tables 4 and 11, eliminating duplicates).
Finally, the intersection of of DAR and DEG (k) for each condition was retrieved with the following venn diagram online tool: https://bioinformatics.psb.ugent.be/webtools/Venn/"
* Issue 2: Discrepancies in DEG counts from supplementary table 11
â–ª Resolved: Investigated variable definitions (using the wrong variable - strand), confirmed that log2FoldChange determines up/down-regulation

* Issue 3: Filling in DAR/DHMR values
â–ª Unresolved: Unclear correspondence between "promoters" rows and excel file sheets. Does H3K27me3 correspond to the promoters?
* Issue 4: Using the Venn diagram tool to find intersections
â–ª Unresolved: Worked for one condition (ATC vivo poly (down)) but failed for ATAC vitro-vibrio and ATAC-vivo-vibrio. Tool returns a "Request Entity Too Large" error.
* Issue 5: Define the population size
â–ª Unresolved: The instructions for defining the population size are not clear. In supplementary table 4, it seems that the variable "Gene ID (ENSEMBL)" should be used, but in supplementary table 10, should the variable "Nearest PromoterID" or "Gene symbol" be used?
ïƒ¨ Using supplementary table 11 values to perform hypergeometric test
Having failed to obtain the values required to reproduce supplementary table 11, the data already provided were used to obtain the "enrichment" and "p-value" values using the excel function provided.
* Issue 1: Comparison of p-values
â–ª Resolved: For Up condition, extremely small p-values are not displayed correctly due to Excel's limitations in scientific notation. Excel may either display them as zero or in an incomplete scientific format (e.g., 0.00E+00). Using the tool on the web.
5.4 Statistical comparison Original vs Reproduced results
â—? Results: Based on the available data in supplementary table 11, the "enrichment" and "p-value" values have been successfully reproduced in most cases.
â—? Comments: The full table could not be reproduced, particularly the data corresponding to DAR/DHMR, DAR/DHMR+DEG and population size values, due to missing information or unclear definitions in the supplementary files.
â—? Errors detected: The enrichment value for the Up condition of promoters-vitro-vibrio was incorrectly reported in the manuscript/table. Based on the Excel formula and the online tool used, the correct value appears to be 2.28 instead of 2.82.
â—? Statistical Consistency: All the values that could be reproduced from the available data matched the original results, except for the detected error.
7. Conclusion
* Summary of the computational reproducibility review
The study's results were partially reproduced. Key values such as enrichment and p-values were successfully replicated, but some dataset elements (DAR/DHMR, DAR/DHMR+DEG, and size population) could not be verified due to insufficient methodological details provided in the manuscript. An error in the enrichment value for the Up condition of promoters-vitro-vibrio was identified (2.28 instead of 2.82). The p values used for statistical inference were however successfully reproduced.
* Recommendations for authors
o Improve data documentation: Define variables in supplementary files.
o Provide all code and scripts: Share the excel formulas used for table 4/supplementary table 11.
o Clarify statistical methodology: Include detailed methods description for the hypergeometric test.
o Enhance reproducibility workflow: Provide a structured README with all necessary steps.

Methods

Are the methods appropriate to the aims of the study, are they well described, and are necessary controls included? Choose an item.

Conclusions

Are the conclusions adequately supported by the data shown? Choose an item.

Reporting Standards

Does the manuscript adhere to the journal's guidelines on minimum standards of reporting? Choose an item.

Choose an item.

Statistics

Are you able to assess all statistics in the manuscript, including the appropriateness of statistical tests used? Choose an item.

Quality of Written English

Please indicate the quality of language in the manuscript: Choose an item.

Declaration of Competing Interests

Please complete a declaration of competing interests, considering the following questions:

- Have you in the past five years received reimbursements, fees, funding, or salary from an organisation that may in any way gain or lose financially from the publication of this manuscript, either now or in the future?
- Do you hold any stocks or shares in an organisation that may in any way gain or lose financially from the publication of this manuscript, either now or in the future?
- Do you hold or are you currently applying for any patents relating to the content of the manuscript?
- Have you received reimbursements, fees, funding, or salary from an organization that holds or has applied for patents relating to the content of the manuscript?
- Do you have any other financial competing interests?
- Do you have any non-financial competing interests in relation to this paper?

If you can answer no to all of the above, write 'I declare that I have no competing interests' below. If your reply is yes to any, please give details below.

1. No 2. No 3. No 4. No 5. No 6. No

I agree to the open peer review policy of the journal. I understand that my name will be included on my report to the authors and, if the manuscript is accepted for publication, my named report including any attachments I upload will be posted on the website along with the authors' responses. I agree for my report to be made available under an Open Access Creative Commons CC-BY license (http://creativecommons.org/licenses/by/4.0/). I understand that any comments which I do not wish to be included in my named report can be included as confidential comments to the editors, which will not be published.

Choose an item.

To further support our reviewers, we have joined with Publons, where you can gain additional credit to further highlight your hard work (see: https://publons.com/journal/530/gigascience). On publication of this paper, your review will be automatically added to Publons, you can then choose whether or not to claim your Publons credit. I understand this statement.

Yes Choose an item.
